# A dynamic protein interactome drives energy conservation and electron flux in *Thermococcus kodakarensis*

**DOI:** 10.1128/aem.00293-25

**Published:** 2025-04-03

**Authors:** Sere A. Williams, Danielle M. Riley, Teagan P. Rockwood, David A. Crosby, Katherine D. Call, Jared J. LeCuyer, Thomas J. Santangelo

**Affiliations:** 1 Graduate Program in Cell and Molecular Biology, Colorado State University 3447, Fort Collins, Colorado, USA; 2 Department of Biochemistry and Molecular Biology, Colorado State University 548293 , Fort Collins, Colorado, USA; 3 Department of Chemistry, Colorado State University 224023, Fort Collins, Colorado, USA; University of Nebraska-Lincoln, Lincoln, Nebraska, USA

**Keywords:** archaea, oxidoreductase, redox metabolism, electron flow, ferredoxin, affinity purification coupled to mass spectrometry (AP-MS), protein-protein interaction

## Abstract

Life is supported by energy gains fueled by catabolism of a wide range of substrates, each reliant on the selective partitioning of electrons through redox (reduction and oxidation) reactions. Electron flux through tunable and regulated protein interactions provides dynamic routes for energy conservation, but how electron flux is regulated *in vivo*, particularly for archaeal metabolisms that support rapid growth at the thermodynamic limits of life, is poorly understood. Identification of *bona fide in vivo* protein assemblies and how such assemblies dictate the totality of electron flux is critical to our understanding of the regulation imposed on metabolism, energy production, and energy conservation. Here, 25 key proteins in central metabolic redox pathways in the model, genetically accessible, hyperthermophilic archaeon *Thermococcus kodakarensis*, were purified to reveal an extensive, dynamic, and tightly interconnected network of protein interactions that responds to environmental cues (such as the availability of various reductive sinks) to direct electron flux to maximize energetic gains. Interactions connecting disparate functions suggest many catabolic and anabolic activities occur in spatial proximity *in vivo*, and while protein complexes have been historically defined under optimal conditions, many of these complexes appear to maintain alternative partnerships in changing conditions. The totality of the results obtained redefines our understanding of *in vivo* assemblies driving ancient metabolic strategies supporting the growth of modern Archaea.

**IMPORTANCE:**

Given the potential for rational genetic manipulations of biofuel- and biotech-promising archaea to yield transformative results for major markets, it is a priority to define how the metabolisms of such species are controlled, at least in part, by *in vivo* protein assemblies, and from such, define routes of energy flux that can be most efficiently altered toward biofuel or biotechnological gains. Proteinaceous electron carriers (PECs, such as ferredoxins) offer the potential for specific protein–protein interactions to coordinate selective reductive flow. Employing the model, genetically accessible, hyperthermophilic archaeon, *Thermococcus kodakarensis*, we establish the metabolic protein interactome of 25 key redox proteins, revealing that each redox active protein has a dynamic partnership profile, suggesting catabolic and anabolic activities may occur in concert and in temporal and spatial proximity *in vivo*. These results reveal critical importance in evaluating the newly identified partnerships and their role and utility in providing regulated redox flux in *T. kodakarensis*.

## INTRODUCTION

Many biological energy gains are reliant on controlled electron transfers that ultimately generate a chemiosmotic membrane potential that can be exploited to generate ATP (1). Fermentation produces reduced small molecules [NAD(P)H] and reduced proteinaceous electron carriers (PECs; typically ferredoxins [Fds]) that transfer electrons to reductant sinks for energy and biomass production (2, 3). The mechanisms controlling *in vivo* electron transfers and high-confidence identification of the complexes that direct the bulk of electron transfers thus are of critical importance to establish. Electron movements are typically limited to a maximal distance of ~12–14 Å (4), requiring formation of complexes, at least transiently, for controlled electron transfers (5). Fds and small molecules [typically NAD(P)H and FADH_2_] often mediate electron transfers between larger complexes (6, 7). While nucleotide-based electron carriers offer limited specificity to such transfers, PEC-mediated transfers provide large interfaces that can be leveraged to drive selective electron transfer to maximize energy conservation and energy production under changing environmental conditions (8, 9). Electron flux through tunable and regulated systems based on selective protein–protein interactions offers flexibility in energy conservation strategies under energy-limiting conditions, providing fitness benefits to organisms that can exploit different electron disposal pathways. Limited information, however, exists on the *in vivo* compositional dynamics of assemblages and the specificities of the electron transfers that underlie energy production and conservation strategies.

*Thermococcus kodakarensis* is a fast-growing, anaerobic, hyperthermophilic archaeon that encodes a modified Embden-Meyerhof Parnas (EMP) glycolytic pathway and catabolizes amino acids (Fig. S1) (10–12). Breakdown of amino acids, chitin, and carbohydrates yields minimal energetic gains via substrate-level phosphorylation (13, 14), and rapid growth is thus dependent on the activities of two alternative membrane-bound reductases that couple terminal electron transfers to production of a membrane gradient that can be exploited for ATP production (15–17). The membrane-bound sulfane reductase (MBS) uses elemental sulfur (S˚) as the terminal electron acceptor (16, 18), but when S˚ is unavailable, *T. kodakarensis* couples the generation of a chemiosmotic gradient to hydrogen production via the reduction of protons via a membrane-bound hydrogenase (MBH) complex (15).

The bulk of electron flux within *T. kodakarensis* is coordinated by PECs, and the selective production of specific Fd isoforms permits regulated transport of electrons from specific electron donors to specific electron acceptors that are reliant on distinct protein partnerships with Fds (6, 8), but the totality of redox protein dynamics and the potential for Fds to mediate or participate in the formation of large complexes of redox donors and acceptors have not been explored. To define the redox protein interactome in *T. kodakarensis,* we repeatedly and reproducibly purified natively assembled redox protein complexes formed *in vivo* from 25 unique *T. kodakarensis* strains to establish the metabolic networks controlling electron flux. Our results define a dynamic and highly interconnected series of complexes composed of PECs, electron donors, and electron acceptors that redefines the composition of the major metabolic pathways in *T. kodakarensis,* adumbrating that compositional alterations may be linked to fitness-relevant activity changes of central redox chemistries. More broadly, our results imply that the selectivity of protein associations offers an underappreciated and understudied mechanism to control electron flux to maximize energy conservation strategies under distinct environmental conditions.

## RESULTS

### Purification of *in vivo* protein assemblies

To establish the *in vivo* protein networks that support the major redox pathways that shuttle electrons through central metabolism, we took advantage of the facile genetic system for *T. kodakarensis* to markerlessly modify the genome, such that the genes encoding individual components of known redox-relevant protein complexes were extended with sequences that encode a nine amino acid hemagglutinin (HA) epitope and six histidine (His_6_) affinity sequence on the mature polypeptide (Fig. S2A) (19–22). Starting with a common parental strain (TS559) (23), we constructed 25 otherwise isogenic strains of *T. kodakarensis* (Table 1), whose genomes were modified, such that the resultant strain generated an HA and His_6_-tagged redox protein from the native locus. It was critical to generate strains through markerless genomic modifications to ensure retention of the native genomic context, inclusive of promoters and regulatory elements that control RNA levels and protein expression, as any mechanism that alters the steady-state level of the proteins *in vivo* introduces the possibility of altering the native complexes that support electron shuttling. Each strain was produced with a now-routine markerless genomic modification strategy (19, 20).

**TABLE 1:** *T. kodakarensis* genes targeted for generating the metabolic protein interactome

Gene	Annotated protein	Acronym and function	Composition, no. of tagged subunits	Source(s)
TK0135	Indolepyruvate-Fd oxidoreductase, β subunit	IOR; breakdown of aromatic amino acids	2α2β, 2/2	(24)
TK0136	Indolepyruvate-Fd oxidoreductase, α subunit			
TK0650	Rubrerythrin-related protein	RBR1; Fe-containing protein involved in oxidative protection; peroxidase function	Likely homodimer, 1/1	(25)
TK0826	Rubrerythrin Mn-catalase	RBR2; Fe-containing protein involved in oxidative protection; peroxidase function	Likely homodimer, 1/1	(25)
TK1088	Geranylgeranyl reductase	GGR; reduces isoprenoid chains producing fully saturated lipids	Monomer, 1/1	(26)
TK1123	2-Oxoacid:ferredoxin oxidoreductase, γ_1_ subunit	OGOR; breakdown of glutamate into acyl-CoA and CO_2_; oxidative decarboxylation of deaminated glutamate	Tetramer (αβδγ) and octamer (2α2β2δ2γ), 3/7^*a*^	(13, 27)
TK1129	2-Oxoacid:ferredoxin oxidoreductases, β_2_ subunit			
TK1130	2-Oxoacid:ferredoxin oxidoreductase, α_2_ subunit			
TK1215	Membrane-bound sulfane reductase, NiFe-hydrogenase large subunit 2, MBS-L	MBS; membrane-bound respiratory complex generates a H^+^/Na^+^ gradient and evolves sulfane sulfur in the presence of S˚	13-subunit membrane-bound complex, 1/13	(16)
TK1325	Ferredoxin:NADP oxidoreductase, α subunit	FNOR1; oxidizes Fd to generate NAD(P)H; more abundant in +S˚	αβ, 2/2	(28)
TK1326	Ferredoxin:NADP oxidoreductase, β subunit			
TK1684	Ferredoxin:NADP oxidoreductase, α subunit	FNOR2; oxidizes Fd to generate NAD(P)H; more abundant in –S˚	αβ, 2/2	(28)
TK1685	Ferredoxin:NADP oxidoreductase, β subunit			
TK1978	2-Oxoisovalerate-ferredoxin oxidoreductase, γ subunit	VOR and POR	αβδγ, 4/4	(29)
TK1979	2-Oxoisovalerate-ferredoxin oxidoreductase, δ subunit	VOR; breakdown of branched-chain amino acids into acyl-CoA and CO_2_		
TK1980	2-Oxoisovalerate-ferredoxin oxidoreductase, α subunit			
TK1981	2-Oxoisovalerate-ferredoxin oxidoreductase, β subunit			
TK1982	Pyruvate:ferredoxin oxidoreductase, δ subunit	POR; breakdown of pyruvate into acetyl-CoA and CO_2_	αβδγ, 4/4	(13, 30)
TK1983	Pyruvate:ferredoxin oxidoreductase, α subunit			
TK1984	Pyruvate:ferredoxin oxidoreductase, β subunit			
TK2075	[4Fe-4S] cluster-binding protein associated with FDH, FDHγ_2_	FDH; reversible conversion of formate into CO_2_	Unknown, 4/~6	(31)
TK2076	Formate dehydrogenase, FDHα			
TK2077	Formate dehydrogenase, [4Fe-4S] cluster-binding protein, FDHγ_3_			
TK2078	[4Fe-4S] cluster-binding protein associated with FDH, FDHγ_4_			
TK2163	Glyceraldehyde-3-phosphate:ferredoxin oxidoreductase	GAPOR; converts glyceraldehyde-3-phosphate to 3-phosphoclycerate in glycolysis	Monomer, 1/1	(32)

^*a*^ While the OGOR functional protein complex is predicted to be formed from eight subunits (2α2β2δ2γ), only one obvious δ subunit is encoded within *T. kodakarensis*, and thus a total of seven genes are annotated as OGOR-encoding genes, three of which were tagged in this study.

Modified strains were confirmed via PCR, and then the entire ~2.08 Mbp genome of each strain was sequenced at >100-fold coverage to eliminate any concerns of second site mutations (Fig. S2D through F). Strains expressing tagged proteins were grown anaerobically at 85°C in rich media to mid-exponential phase before chilling, harvesting, and lysing biomass, and gently purifying the target proteins in complex with their native protein partners (Fig. S3A) (20, 21). Given the large impact of the availability of the preferred terminal electron acceptor, elemental S˚ (33, 34), we grew each strain in triplicate in the absence and presence of S˚ to establish if the known metabolic changes resultant from S˚ availability resulted in significant changes to the redox protein network *in vivo*. While *T. kodakarensis* can utilize glycolysis and gluconeogenic pathways, all strains were grown in the presence of pyruvate, with the exception of strains wherein glyceraldehyde-3-phosphate oxidoreductase (GAPOR, TK2163)—which is primarily active in glycolytic conditions—was tagged; protein interactions within the GAPOR-tagged strain were also evaluated in the absence of pyruvate (–P).

**Fig 1:**
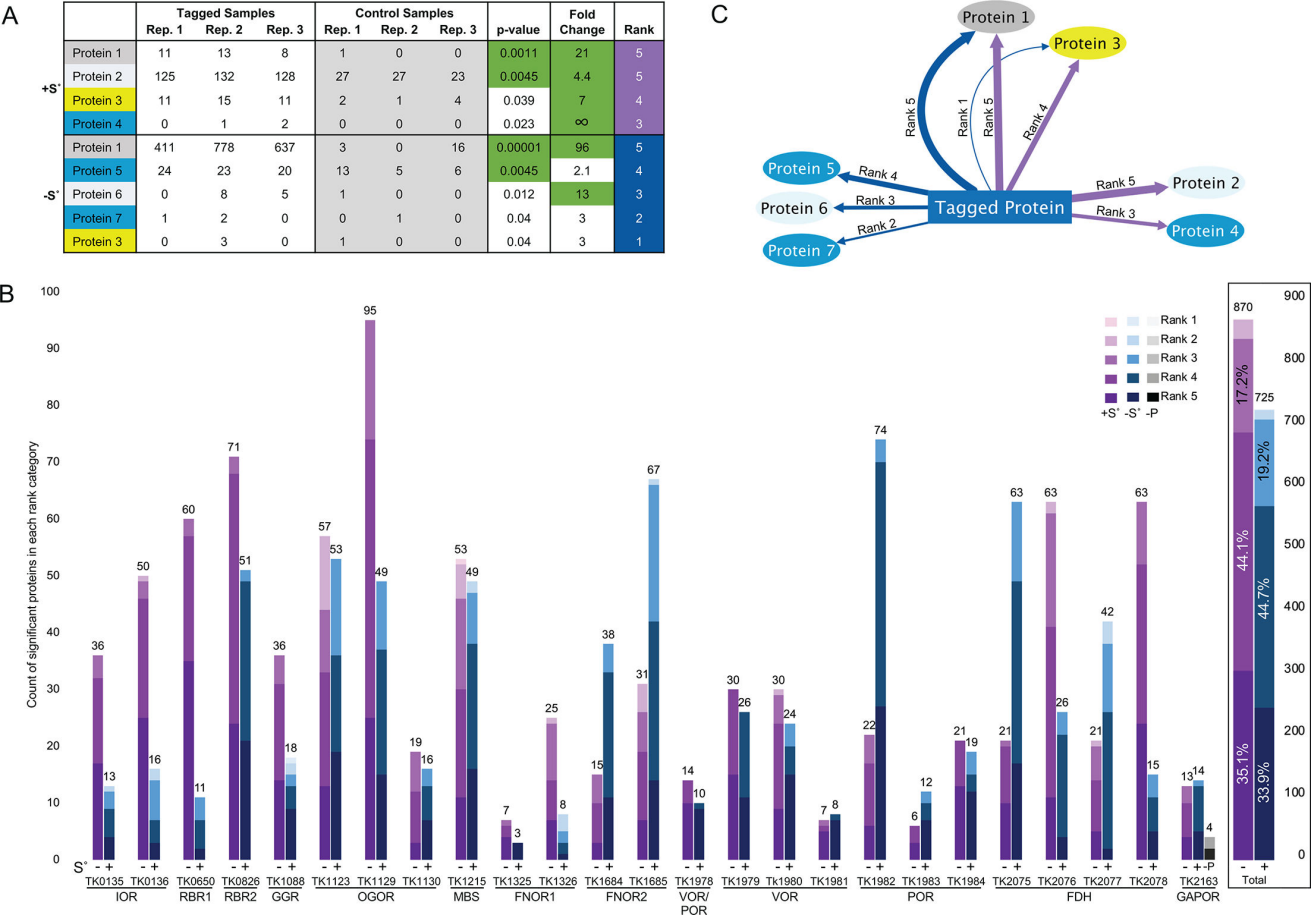
AP-MS identifies *in vivo* partnerships of tagged proteins at native expression levels. (**A**) Spectral counts of peptides are mapped to the *T. kodakarensis* proteome. Proteins identified in tagged samples are compared to those identified in the parent strain (a near isogenic control, TS559). A Fisher’s exact test is used to determine statistically significant, meaningful differences between tagged and control samples (*P* value < 0.05, log_2_ fold change ≥ 2). Proteins that pass all statistical thresholds are ranked (column at far right), such that proteins that appear at a *P* value < 0.01 and a log_2_ fold change >4, with spectral counts in all tagged replicate samples, earn a rank of 5. The rank score decreases by one point for each category not met. (**B**) Tagged proteins retained, on average, ~30 (range of 3–95) highly reproducible protein partnerships with changes introduced under different metabolic strategies induced by the absence (blue) or presence (purple) of the preferred terminal electron acceptor S˚. Protein partnerships are ranked and tallied for each target protein identified by the encoding gene number (e.g., TK0135) and complex acronym (e.g., IOR). The total protein associations copurified for each tagged protein are noted at the top of each bar, and the rank score of each partner is indicated by increasing shade color. At far right, a total of 870 and 725 proteins were identified as significant in +S˚ and –S˚ conditions, respectively, from these 25 tagged proteins. The majority of proteins (~44%) were identified with a rank score of 4, while ~34% were identified with a rank score of 5, followed by ~18% identified with a rank score of 3. Associations observed for GAPOR in S˚ conditions lacking pyruvate (–P) are depicted using a gray scale. (**C**) Proteins (ellipses) copurified with the tagged target protein (rectangle at center) in sulfur (+S˚, purple connecting lines) and non-sulfur (–S˚, blue connecting lines) conditions are visualized, such that associations with higher rank are shown with thicker lines and associations with lower rank scores are shown with thinner lines. Significant proteins are shown in colored ellipses (identified by gene number: TKxxxx) and generally arranged from lower gene numbers on the left to higher gene numbers moving right. While the majority of copurifying proteins are shown in blue, redox-associated proteins of special interest are displayed in varying colors, which match for proteins of similar annotation or within the same operon.

Affinity purification of the target protein via immobilized metal affinity chromatography (IMAC) allowed the retention of protein partnerships established *in vivo* (Fig. S3A) (21, 35–37). We relied on Western blotting with anti-HA antibodies to identify fractions from IMAC purifications that contained the HA-tagged target protein (Fig. S3B through E). IMAC fractions containing the target protein were pooled, and the total proteins contained within were identified by multi-dimensional protein identification technology (MuDPIT) (38, 39). Our use of affinity purification-mass spectrometry (AP-MS) techniques does not rely on artificial crosslinking nor altered protein expression levels and thus reports the naturally occurring protein assemblages. To limit the identification of false positives, the total proteins present in fractions from three biological replicates containing HA-tagged target proteins were compared to the total proteins identified in identical IMAC fractions generated from up to four biological replicates of the parental strain (TS559) to remove native *T. kodakarensis* proteins that have a natural, albeit modest, affinity for the Ni^2+^-chelating resin (Fig. S3A). Finally, we applied stringent statistical parameters (Fig. 1A) to ensure that copurifications were reproducible and to establish whether the individual signal for each significant protein in experimental samples was sufficiently greater than the signal returned by the respective protein from control (parental) strains. A minimum *P* value of 0.05 was required to provide confidence that a protein could be uniquely identified by tandem MS, and for proteins of average size, this typically resulted in repeated recovery of one or more unique tryptic fragments. We established a minimal cutoff of a log_2_ ≥ 2-fold change to identify proteins as enriched in IMAC fractions from purifications from experimental over control strains (Fig. 1A).

### Redox protein assemblies are reproducibly purified and reveal a highly interconnected redox network

The confidence and reproducibility of copurifying any uniquely identified protein in high abundance within experimental samples were quantized within a 1–5 ranking system (5 = extreme confidence and reproducibility in all replicates) that details nuances in the dynamics and stability of *in vivo* redox protein associations (Fig. 1A). The assignment of a rank score to every interaction provides a confidence metric for the protein association without data loss, as would occur upon imposing increasingly stringent thresholds. Proteins that were identified in each of three replicates at a *P* value of <0.01 and with a log_2_ fold change ≥4 received a rank of 5 (Fig. 1A). About one-third (~35%) of all proteins were identified with a rank score of 5 (Fig. 1B). Proteins that were identified in fewer than three replicates at a *P* value of 0.01–0.05 or with a log_2_ fold change >2 (but less than 4) earned a rank score, which was reduced by one point for each missing parameter. Almost half of the remaining interactions (~45%) had a rank score of 4, ~18% scored a 3, and just ~2% scored a 2 or 1 (Fig. 1B). Thus, while all reported proteins pass standard significance thresholds, many passed more stringent thresholds (e.g.*,* >95% of interactions were identified with a rank score ≥3). To visually depict the rank score of each interaction, we vary the line thickness connecting copurifying proteins (5 = thick, 1 = thin) (Fig. 1C).

**Fig 2:**
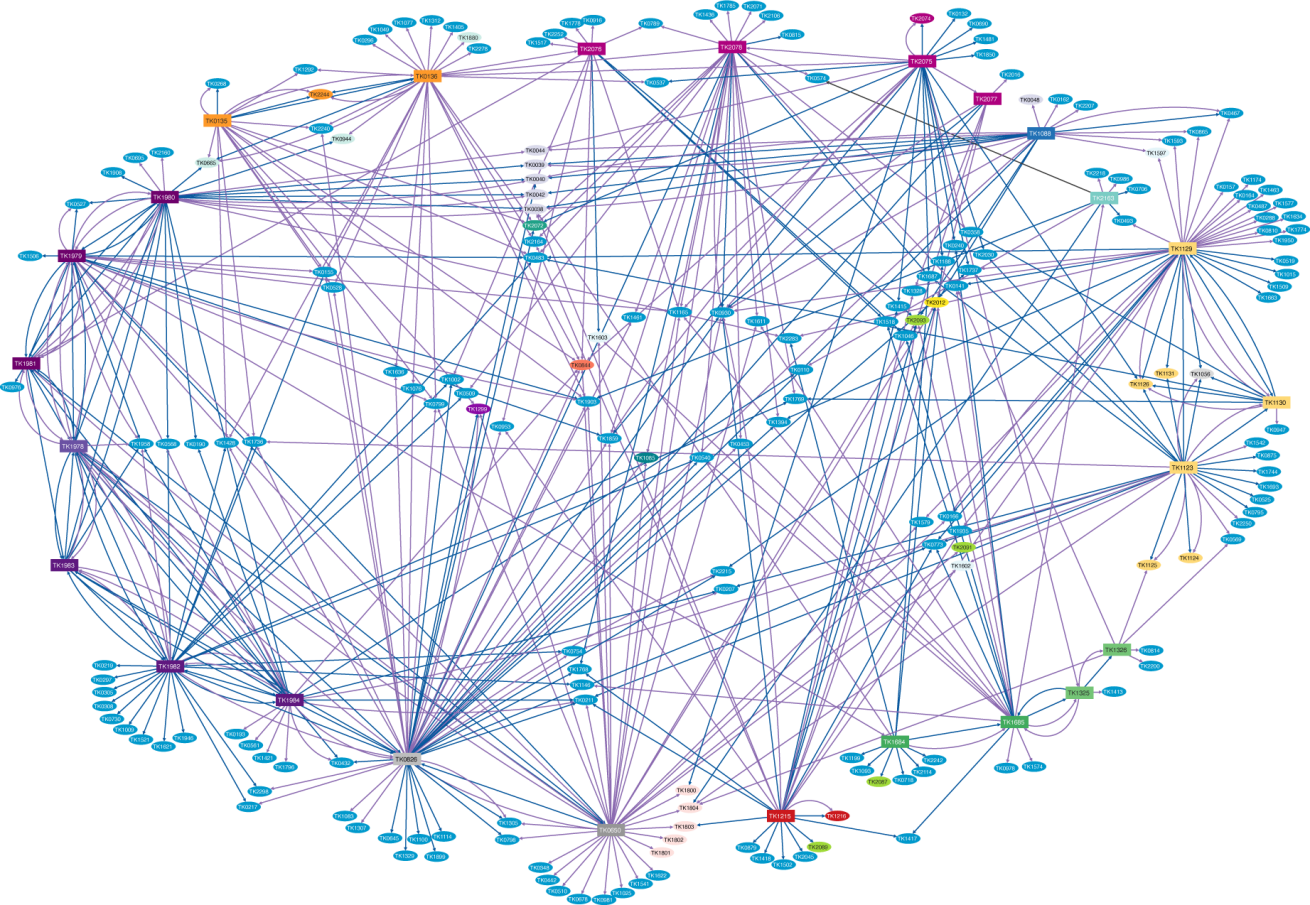
The redox protein metabolic network of *T. kodakarensis* is highly interconnected. The global redox protein network of rank 5 protein associations is shown. Tagged proteins in colored rectangles copurified proteins in colored ellipses in +S˚ (purple lines) or –S˚ (blue lines) conditions. Tagged proteins are arranged on the periphery, and subunits that are known or predicted to form complexes are colored to match: beginning at the top and moving clockwise, FDHs (fuchsia), GGR (dark blue), GAPOR (seafoam green), OGORs (mustard), FNORs (green), MBS (red), RBRs (gray), PORs (dark purple), VORs (plum), and IORs (orange). The majority of copurified proteins are displayed as bright blue ellipses, but subunits of some metabolically relevant complexes have been colored differently to stand out (e.g.*,* MBH, TK2080-2093, lime green). Proteins that were copurified with only a single tagged protein are placed radiating outward from the network, while proteins that were independently copurified with multiple tagged proteins are located in the interior of the matrix (Cytoscape v3.10.1.).

The ensemble redox protein network (restricted to rank 5 associations, Fig. 2) reveals that ~10% (221) of the ~2,306 predicted proteins encoded within *T. kodakarensis* are identified with extreme confidence within complexes formed by the collectively purified 25 proteins. AP-MS is exceptionally sensitive and specific, allowing an average of ~515 proteins (range 112–822) identified from each of our >177 purifications. Our definition of *bona fide* partnerships resulted in just ~6% (on average; range 0.5%–20%) of the total proteins identified to exceed the minimum thresholds (*P* value < 0.05, log_2_ fold change ≥2) for a confident association to be defined within our experimental samples. Despite such stringent statistical thresholds, each tagged protein revealed ~30 interaction partners on average, with the availability of S˚ often altering the total number of high-confidence interactions for different tagged proteins (Fig. 1B). While confidence rankings are visualized via line thickness, line color defines whether interactions were identified in the absence (blue) or presence (purple) of S˚ (Fig. 1C and 2).

### Complexes maintain redox-preferred associations with larger assemblies generally forming in the absence of S˚

**Fig 3:**
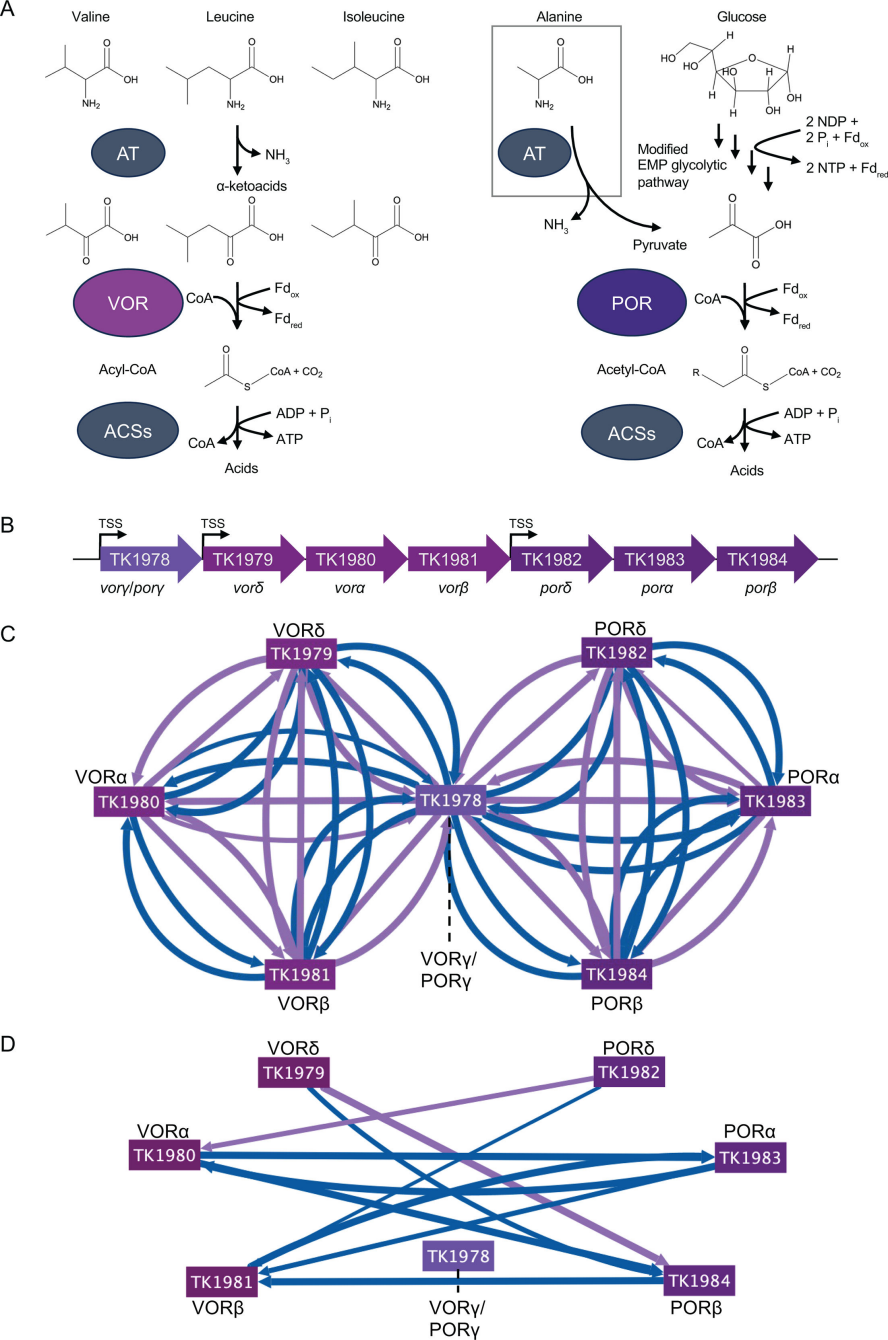
POR and VOR complexes dynamically associate *in vivo*. (**A**) At the left, branched-chain amino acids are deaminated by an amino transferase (AT) to produce α-keto acids (or 2-oxoacids). VOR (plum ellipse) preferentially catalyzes the oxidation of α-keto acids into acyl-CoA while generating a reduced Fd. Acyl-CoA synthase (ACS) generates ATP, recycles CoA, and releases excreted acids. At the right, *T. kodakarensis* utilizes a modified EMP pathway in glycolysis, generating a net gain of just two ATP and two reduced Fds for the conversion of glucose into pyruvate. POR (dark purple) catalyzes the conversion of pyruvate into acetyl-CoA and CO_2_ coupled to reduction of a Fd. Note that pyruvate is homologous to an α-keto acid from a deaminated alanine (box at the top), thus POR can additionally act on substrates from amino acid fermentation pathways. (**B**) POR and VOR share a γ subunit encoded by the first gene (purple, TK1978) in the *vor/por* operon, TK1978-1984. The remaining three subunits that make up the POR heterotetramer (dark purple, TK1982-TK1984) are encoded directly downstream of *vor* (plum, TK1978-TK1981). Three primary transcription start sites (TSS) appear upstream of TK1978, TK1979, and TK1984. (**C**) All seven POR/VOR subunits were tagged, and most tagged subunits identified all three of the other subunits of their respective complexes in both +S˚ (purple lines) and –S˚ (blue lines) conditions. (**D**) Intra-complex associations have been removed, and instead, associations between complexes (excluding VORg, TK1978) are shown. Eight inter-complex associations are observed in –S˚ while just two inter-complex associations are observed in +S˚.

Ketoisovalerate oxidoreductase (VOR; also known as 2-oxo-isovalerate oxidoreductase) catalyzes the conversion of 2-oxoacids (also known as 2-ketoacids) with branched side chains into acyl-CoA and CO_2_ while reducing a Fd (Fig. 3A, left; Fig. S1) (13, 29). *In vitro* purifications and characterizations of VOR revealed an αβδγ heterotetramer structure that appears highly conserved within the Thermococcales (40). VOR is encoded within an operon (TK1978-TK1981) in *T. kodakarensis* (Fig. 3B) (33), and we tagged and purified each VOR subunit. As was anticipated from a tightly associated and stable complex, purifications of VOR—regardless of the subunit that was tagged—revealed the reciprocal presence of all subunits in high confidence in every combination (Fig. 3C).

While each subunit of VOR identified each of the other subunits of VOR, individual VOR subunits also revealed more unique partnerships with non-VOR subunits (Fig. S4, Tables S1 to S4). While the protein product of TK1981 (VORb) retained just three high-confidence interactions beyond the VOR complex itself, TK1979 (VORd) and TK1980 (VORa) each revealed an additional ~20–25 high-confidence *in vivo* protein associations that suggest important interactions with VOR that should be investigated further. Given that most of the proteins identified as auxiliary interactions with individual VOR subunits were not identified with each VOR subunit, there is little evidence to support that most of these proteins are stable components of VOR. In contrast, and quite unexpectedly, the high-confidence, repetitive, and reproducible recovery of every subunit of the pyruvate oxidoreductase (POR) complex with multiple VOR subunits, and purification of VOR subunits in assemblies recovered from POR-based purifications, support that POR and VOR are regularly complexed *in vivo* (Fig. 3C and D).

POR, which converts pyruvate to acetyl-CoA and CO_2_ while reducing a Fd (Fig. 3A, right; Fig. S1), has been biochemically characterized as an octamer composed of four subunits (2α2β2γ2δ) encoded by TK1978 and TK1982-TK1984, and all subunits were tagged for AP-MS analysis (Fig. S5; Tables S1, S5 to S7) (27, 30, 40, 41). *Vor* and *por* are thus encoded immediately adjacent in the *T. kodakarensis* genome (Fig. 3B), and while algorithms that predict microbial operons consider the entire genomic region encoding *vor* and *por* (TK1978-TK1984) within a single operon (42, 43), analysis of the *T. kodakarensis* transcriptome identified three primary transcription start sites preceding TK1978, TK1979, and TK1982, respectively (Fig. 3B) (44). The expression of the γ subunit (TK1978) thus appears uncoupled from the expression of the remaining three genes encoding either *vor* (TK1979-TK1981) or *por* (TK1982-TK1984).

While VOR and POR function as independent multimeric complexes, our data define that multiple subunits of each respective complex tightly associate with the other complex, predominantly when S˚ is not available (Fig. 3D). The abundant and reciprocal associations observed between POR and VOR argue that the two complexes exist *in vivo* as a large assemblage. Surprisingly, the tight associations of POR and VOR are essentially lost under physiological conditions containing S˚, which is not explained by differences in the expression of the protein complexes in varying redox states (44). The presence of the POR–VOR hetero-complex in –S˚ implies that the POR–VOR partnership is not obligatory but rather a potential strategy in sulfur-limited conditions that requires further investigation to reveal the putative fitness advantages and regulation of electron flux that likely depends on the co-association of POR and VOR.

**Fig 4:**
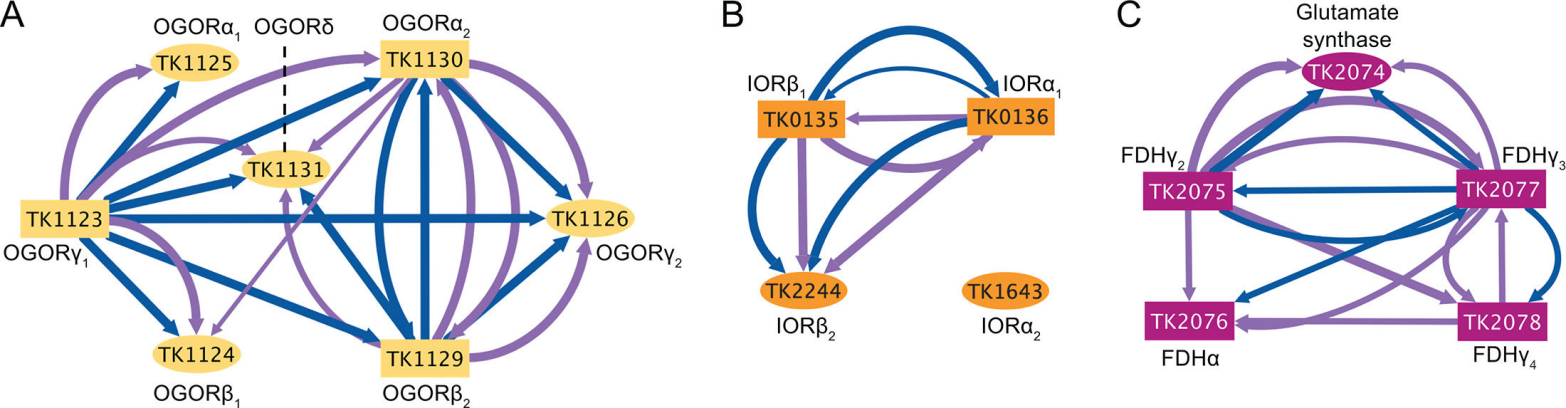
Presumptive *in vivo* redox complex assemblages are redefined by AP-MS. (**A**) Seven genes encode the proposed OGOR heterooctamer: TK1123–TK1126 and TK1129–TK1131. When the OGORα_2_ (TK1130), OGORβ_2_ (TK1129), and OGORγ_1_ (TK1123) subunits were tagged (mustard rectangles), AP-MS identified all seven subunits of the proposed 2α2β2δ2γ heterooctamer. While OGORα_1_ was only copurified with OGORγ_1_, all other subunits were copurified by multiple OGOR-tagged subunits, suggesting that while there could be 2α_1_2β_1_2δ2γ_1_ or 2α_2_2β_2_2δ2γ_2_ (an octamer composed of homodimers) complexes, the full heterodimeric (α_1_α_2_β_1_β_2_2δγ_1_γ_2_) complex is likely abundant. While OGORa_2_ may associate with OGOR_1_ subunits more than the other OGOR_2_ subunits, OGORγ_1_ identified OGORβ_2_ and γ_2_ only in –S˚ conditions, suggesting that there may be a preference for the heterooctameric confirmation in –S˚ conditions. (**B**) IOR subunits α_1_ and β_1_ (TK0136 and TK0135, respectively) reproducibly and reciprocally copurify, supporting regular association of protein products of these operonic encoded genes. Two non-operonic genes encoding an additional IOR α and β protein are annotated in the genome (TK1643, IORα_2_ and TK2244, IORβ_2_), and while both IORα_1_ and IORβ_1_ identified IORβ_2_, no tagged IOR proteins identified IORα_2_, suggesting that the functional *in vivo* IOR tetramer(s) may be composed as a 2α_1_2β_1_, 2α_1_2β_2_, or 2α_1_β_1_β_2_ complex and that IORα_2_ does not associate with the other IOR subunits. (**C**) TK2075, TK2077, and TK2078 are annotated as [4Fe-4S] cluster-binding proteins associated with FDH and are considered FDHγ_2-4_, while TK2076 is annotated as FDHα. FDHγ_2-4_ reciprocally copurified with one another, and FDHγ_2_ and FDHγ_3_ also copurified with TK2074, which is annotated as a glutamate synthase but homologous to *To*-FDH3B (31). While the FDHγ subunits copurify FDHα (predominantly in +S˚ conditions), FDHα did not copurify any FDHγ subunits, suggesting that FDHα may function independently, especially in –S˚ conditions. Associations seen in +S˚ are shown in purple, while associations seen in –S˚ are shown in blue (Cytoscape v3.10.1). Line thickness corresponds to the rank observed for each association, with a thicker line representing a higher rank score.

Of the known 2-oxoacid catabolizing complexes, 2-oxoacid-ferredoxin oxidoreductase (or 2-ketoglutarate-ferredoxin oxidoreductase, OGOR) displays the greatest substrate specificity by preferentially acting on deaminated glutamate (Fig. S1 and S6A). OGOR has been reported to function as a heterotetramer (13) and a heterooctamer (27) with at least seven putative OGOR subunits (TK1123-TK1126 and TK1129-TK1131) encoding two α (TK1125 and TK1130), two β (TK1124 and TK1129), two γ (TK1123 and TK1126), and one δ (TK1131) subunits (Fig. S6B) (45). We tagged and purified three putative subunits of OGOR (OGORγ_1_ [TK1123], OGORβ_2_ [TK1129], and OGORα_2_ [TK1130]), and high-confidence recovery of all seven putative OGOR subunits provides support for complexes formed from OGOR_1_, OGOR_2_, or OGOR_1_ and OGOR_2_ subunits *in vivo* (Fig. S6C and D, Tables S8 to S10). Similar to the VOR–POR complex(es), while the two OGOR_2_ subunits (α_2_ and β_2_) primarily copurified with other OGOR_2_ subunits in support of a tetrameric complex, OGORγ_1_ demonstrated more dynamic associations (to all six OGOR subunits) when S˚ was lacking (Fig. 4A). These data combined suggest that OGOR_1_ and OGOR_2_ may function as tetramers or OGOR_1_- or OGOR_2_-octamers, with a slight preference for heterooctameric activity in –S˚ conditions (Fig. 4A). It will be important to establish if OGOR complexes of different compositions retain unique biochemical properties and what putative advantages are gained by forming multiple OGOR complexes *in vivo*.

### Heterogeneous and multiple redox-active *in vivo* assemblages are likely

Like OGOR, the composition(s) of complexes defined as indolepyruvate oxidoreductases (IORs) was revealed through our AP-MS techniques to be heterogeneous and not fixed *in vivo*. IOR catabolizes the oxidative decarboxylation of aryl pyruvates following transamination of aromatic amino acids and likely functions as a 2α2β tetramer (Fig. S1 and S7A) (13, 24). Putative *ior*α_1_ (TK0136) and *ior*β_1_ (TK0135) subunits are encoded adjacent, and their protein products reciprocally identified one another repeatedly in copurifications in both +S˚ and –S˚ conditions (Fig. 4B; Fig. S7B and C; Tables S11 and S12). We identified a second, non-operonic IORβ_2_ (TK2244), which was found to associate with both IORα_1_ and IORβ_1_ in +S˚ and –S˚, suggesting that the IOR complex may consist of hetero β (β_1_β_2_) subunits (Fig. 4B). A second, non-operonic IORα_2_, TK1643, is also annotated in the genome (45); however, TK1643 was not identified as a significant protein partner of either IORα_1_ or IORβ_1_. Our results suggest that the IOR complex in *T. kodakarensis* is composed of α_1_β_1_β_2_ subunits (possibly as 2α_1_β_1_β_2_), leaving an unknown role for the protein product of TK1643. It is also plausible that two IOR complexes, one composed of TK0135-TK0136 (2α_1_2β_1_) and another composed of TK0136-TK2244 (2α_1_2β_2_) exist *in vivo*. Thus, three tetrameric IORs are plausible (2α_1_β_1_β_2_, 2α_1_2β_1_, 2α_1_2β_2_), and it will be necessary to define any distinctions in activities between IOR complexes composed with different subunits.

A final example of heterologous complex formation is provided by our analysis of the formate dehydrogenase complex (FDH) that can recycle the CoA moiety by conversion of acyl-CoAs and formate to CO_2_, generating a reduced Fd (Fig. S1 and S8A) (31, 46, 47). While FDH is well studied in the closely related *Thermococcus onnurineus* (31)*,* the specific composition of the likely tungsten-containing enzymatic complex in *T. kodakarensis* has yet to be determined. *T. kodakarensis* encodes two genomically disparate putative *fdh*α subunits (TK0214 and TK2076), and while the genomic region of TK0214 is bracketed by two hypothetical proteins, the genomic region of TK2076 is adjacent to both the *mbh* operon and genes encoding multiple putative [4Fe-4S] cluster-binding proteins that are plausibly FDH-related proteins, TK2073-TK2079 (Fig. S8B) (31, 45). Thus, FDHα (TK2076) and the adjacent genes encoding FDHγ_2-4_ (TK2075-TK2078) proteins were tagged and analyzed via AP-MS (Fig. S8C and D; Tables S13 and S16). In the presence of S˚, FDHγ_2_ and FDHγ_3_ identified all other tagged FDH subunits with high confidence, thus supporting a stable FDH assemblage containing FDHγ_2-3_ proteins (Fig. 4C). In contrast, FDHα did not identify any other tagged FDH-related proteins, suggesting that it is excluded from the FDH complex *in vivo* and may function independently of such. FDHγ_2-3_ identified one another, along with FDHγ_4_ and glutamate synthase (TK2074) in ±S˚; however, FDHγ_4_ only identified FDHγ_3_ and FDHα in +S˚ and no other FDH subunits in –S˚. Our data suggest that FDHγ_2-3_ regularly associate, while FDHγ_4_ is only an occasional partner, and that FDHα is not part of the FDH complex (Fig. 4C). Additionally, it appears that FDHγ_2-3_ maintain more than double the associations in the absence of S˚, while FDHα and FDHγ_4_ display double the associations when S˚ is present, again suggesting that the *in vivo* metabolic network responds to the availability of the preferred terminal electron acceptor (Fig. 1B; Fig. S8C and D).

### Catabolic and anabolic redox chemistries may be orchestrated in concert at the membrane

The MBS respiratory complex generates a chemiosmotic gradient at the membrane by catalyzing electron transfer to the conversion of polysulfides into reduced sulfanes, which is thought to spontaneously convert to hydrogen sulfide gas (H_2_S) as the terminal reductive sink (Fig. S1) (1, 16). The 13-subunit complex (TK1226-TK1214, MBS-A - N) consists of a membrane-bound arm (MBS-A - I, M), which includes both sodium and proton translocation modules, and a cytoplasmic arm (MBS-J, K, L, and N), which maintains three [4Fe-4S] centers (16). While MBS is lowly expressed in –S˚ conditions (44), AP-MS is sufficiently sensitive to detect ~50 MBS-L associations ±S˚ (Fig. 1B; Fig. S9; Table S17).

**Fig 5:**
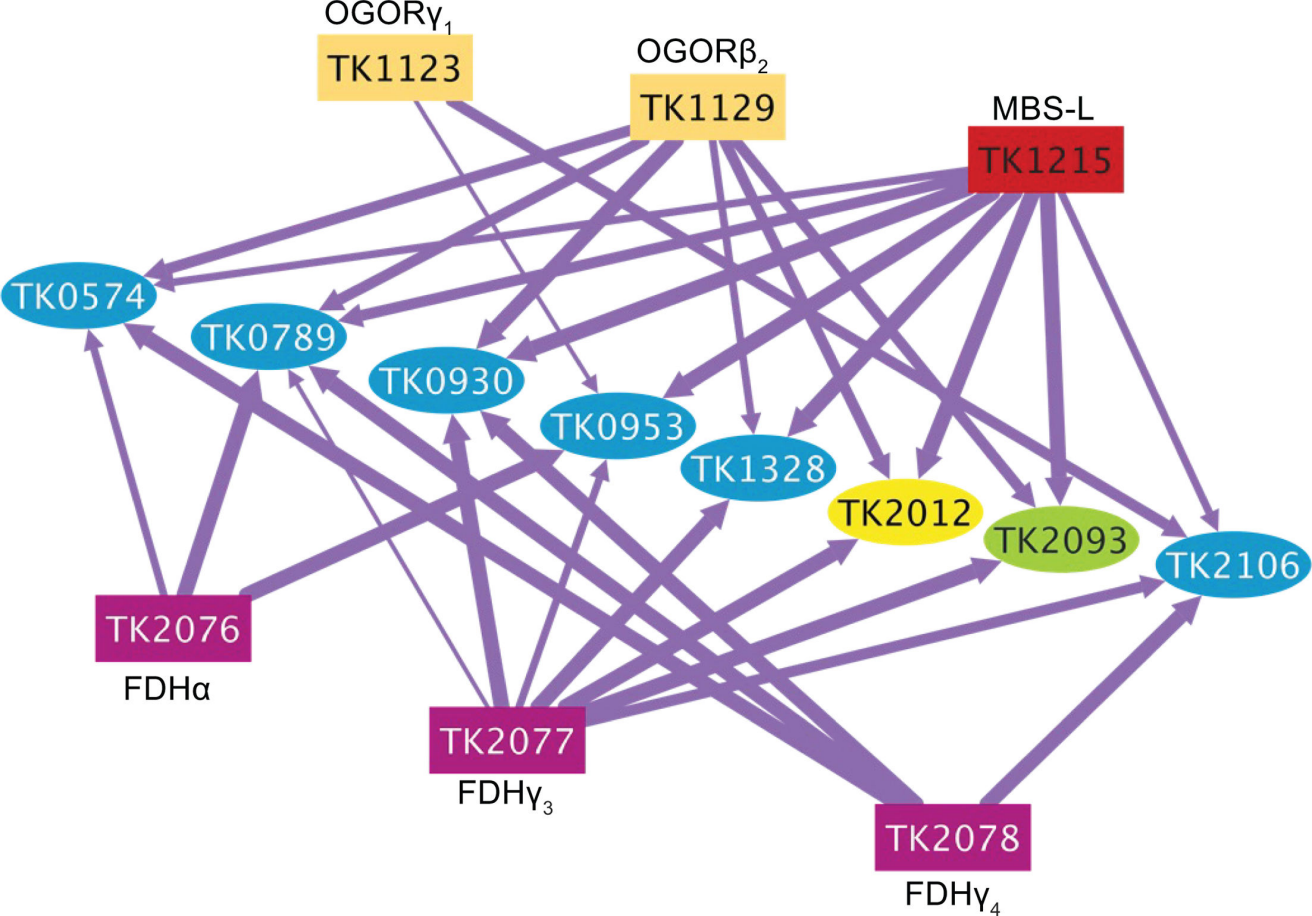
OGOR, FDH, and MBS form a highly interconnected network, linking disparate metabolic functions in sulfur conditions. OGOR (mustard rectangles), MBS (red rectangle), and FDH (fuchsia rectangles) copurify the same eight proteins (bright blue ellipses), including Fd3 (TK2012, yellow ellipse) and MBH-N (TK2093, lime green ellipse) in sulfur conditions. Line thickness corresponds to the rank observed for each association, with a thicker line representing a higher rank score. Gene annotations for associated proteins are as follows: TK0574, metallophosphoesterase, calcineurin superfamily; TK0789, glycerol-1-phosphate dehydrogenase [NAD(P)^+^]; TK0930, uncharacterized protein; TK0953, predicted ATPase, AAA superfamily, containing PIN and KH nucleic acid-binding domains; TK1328, tRNA (1-methyladenosine) methyltransferase; TK2012, ferredoxin 3; TK2093, membrane-bound hydrogenase, [4Fe-4S] cluster-binding subunit, MBH-N; TK2106, enolase. Line thickness corresponds to the rank observed for each association, with a thicker line representing a higher rank score (Cytoscape v3.10.1).

Like POR and VOR associations, tagged MBS and tagged OGOR subunits reciprocally identified multiple subunits of one another in +S˚ conditions, suggesting that these two complexes form a *bona fide* large complex *in vivo* (Fig. 5; Fig. S6C and D, S9; Tables S8 to S10, S17). OGOR oxidizes 2-oxoglutarate (Fig. S1 and S6A) (27), releasing an electron that could be directly transferred to MBS; while MBS can use Fd-mediated electron transfers, it remains plausible that OGOR and MBS may directly transfer electrons or be linked by a Fd that serves as a molecular conduit for electron transfer (8). Twenty-three proteins were reciprocally identified by OGOR and MBS in +S˚ conditions, including Fd3 (Tables S8 to S10, S17). Fd3 may be reduced by OGOR and subsequently provide MBS with an electron, linking amino acid fermentation and the MBS respiratory system at the membrane.

Likewise, tagged MBS and tagged FDH subunits reciprocally identified multiple subunits of one another in +S˚ conditions, suggesting that additional electrons garnered from the conversion of acyl-CoA and formate to CO_2_ by FDH may be donated directly to MBS or via a PEC. Considering that FDH and MBS also both associate with Fd3 (TK2012, Fig. 5), it is theorized that Fd3 aids in transferring electrons not only from OGOR but also from FDH to MBS as well. Twenty-nine additional proteins were identified by at least one subunit of MBS and FDH, suggesting considerable metabolic network connectivity (Fig. S8C and D, S9; Tables S13 to S17; Data Set S1).

Of the 23 similar proteins identified by MBS and OGOR, and the 29 similar proteins identified by MBS and FDH, eight proteins were identified by at least one subunit of FDH, OGOR, and MBS, suggesting that these complexes are linked *in vivo* (Fig. 5). Given that MBS is membrane embedded, OGOR, FDH, and Fd3 may be transiently membrane associated. The potential regulation due to selective intracellular positioning of redox-active components in Archaea has not been investigated, but our results suggest that fitness-relevant regulation is likely imposed through membrane associations and higher-order complex formations.

### Small PECs may mediate or protect higher-order redox complex formations

**Fig 6:**
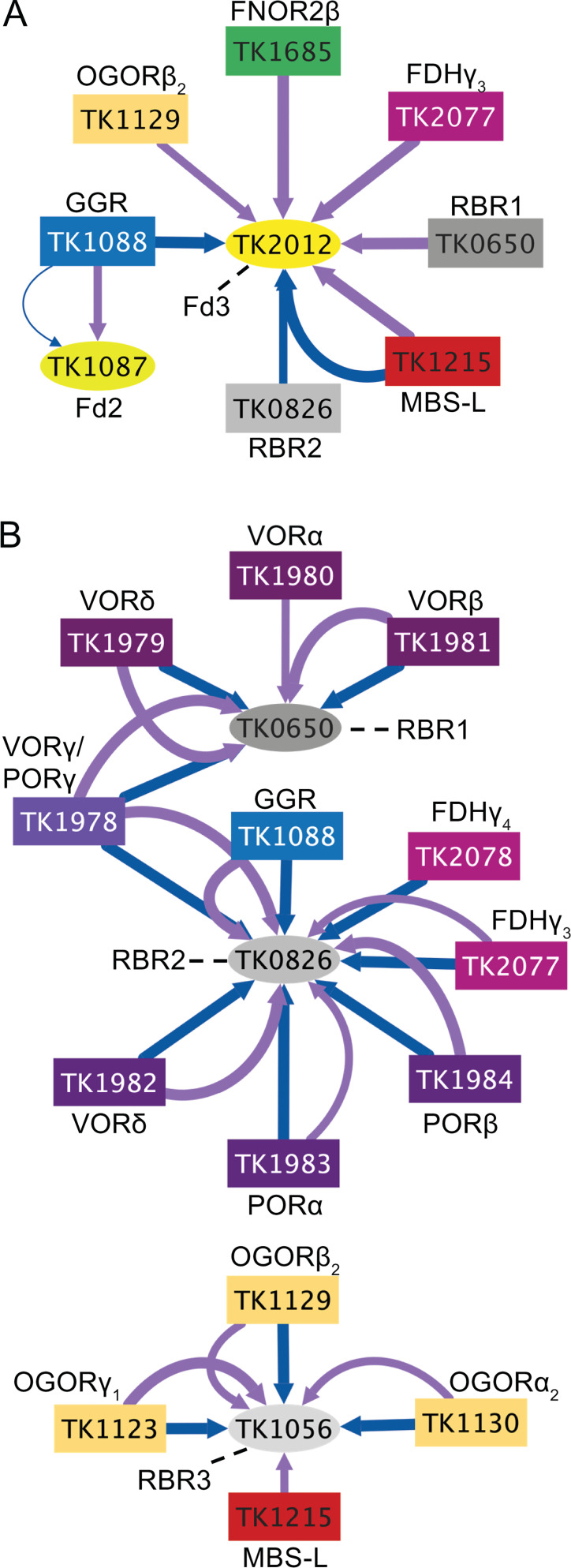
PECs are copurified with interacting partners and likely help maintain redox balance. (**A**) Tagged proteins (rectangles) copurify Fd2 (TK1087, bright yellow ellipse) and Fd3 (TK2012, yellow ellipse). Fd2 (TK1087) was copurified by GGR (TK1088, blue rectangle) in both +S˚ (purple line) and –S˚ (blue line) conditions. GGR also copurified Fd3 in –S˚ conditions. Both RBR1 (TK0650, gray rectangle) and RBR2 (TK0826, light gray rectangle) copurified Fd3, although the associations were observed in different redox conditions: RBR1-Fd3 was observed in +S˚, and RBR2-Fd3 was observed in –S˚. OGORβ (TK1129, mustard rectangle), FNOR2β (TK1685, green rectangle), and FDHγ_3_ (TK2077, fuchsia rectangle) copurified Fd3 in +S˚ conditions, while MBH-L (TK1215) was the only protein to copurify Fd3 in both +S˚ and –S˚ conditions. (**B**) *T. kodakarensis* encodes three rubrerythrins, RBR1 (TK0650, dark gray ellipse), RBR2 (TK0826, gray ellipse), and RBR3 (TK1056, light gray ellipse), which were found to be significant interacting partners in the AP-MS data by 14 different tagged proteins (colored rectangles). All four subunits of VOR (TK1978-TK1981, purple rectangles) copurified RBR1. All four subunits of POR (TK1978, TK1982-TK1984, dark purple rectangles) copurified RBR2. Additionally, FDHγ_3_, FDHγ_4_ (TK2077 and TK2078, respectively, fuchsia rectangles), and GGR (TK1088, blue rectangle) copurified RBR2. Three subunits of OGOR (TK1123-TK1129, mustard rectangles) and MBS-L (TK1215, red rectangle) copurified RBR3. Associations seen in +S˚ are shown in purple, while associations seen in –S˚ are shown in blue (Cytoscape v3.10.1). Line thickness corresponds to the rank observed for each association, with a thicker line representing a higher rank score.

Many of the 25 proteins we tagged and purified were identified as interacting partners of the three unique Fd-isoforms encoded in *T. kodakarensis* (8). Many *in vitro* studies demonstrate that oxidoreductases [such as GAPOR, IOR, OGOR, VOR, POR, and the Fd:NAD(P)^+^ oxidoreductases (FNORs)] perform efficient catalysis when paired with Fds (13, 27, 28, 30, 32, 48–50), and thus we hypothesized that Fd proteins might form molecular bridges between select electron donors and electron acceptors in higher-order molecular assemblages *in vivo*. Unfortunately, LC-MS/MS does not identify all peptides with equal confidences, especially highly charged, very small, or very large tryptic peptides that yield a weak signal or no signal in conventional MuDPIT analyses (51). Thus, while Fd1 (TK1694) is known to be essential and among the most highly expressed transcripts (44), the tryptic fragments of Fd1 are largely not amenable to MuDPIT-based identifications and were not identified in this study. Similarly, the tryptic peptides of Fd2 lie at the detection limit (with one identifiable peptide) and Fd2 could only be confidently identified in a partnership with geranylgeranyl reductase (GGR) (Fig. 6A; Fig. S10; Table S18), confirming an established and well-documented relationship (26, 52–54). Thus, while Fd1 and Fd2 are undoubtedly critical components of *in vivo* redox active complexes, we are unable to detail most of their roles in electron transfers due to technical limitations. Fortunately, the largest Fd isoform, Fd3, yielded tryptic fragments that allowed confident placement of Fd3 within a mega-complex at the membrane composed minimally of OGOR, MBS, and FDH (Fig. 5 and 6A).

Rubrerythrins (RBRs) are non-heme, iron-containing proteins known to alleviate oxidative stress through peroxidase activity (25, 55, 56). *T. kodakarensis* encodes 12 annotated RBRs (45), three of which (TK0650, TK0826, and TK1056; RBR1-3, respectively) appeared significant in the AP-MS data, 7, 15, and 7 times, respectively (Data Set S2). While the three RBRs were not initially predicted to form stable complexes with many of the redox-active complexes *in vivo*, the repeated and reproducible identification of RBRs in our AP-MS analyses warranted the construction of strains wherein the encoding loci of RBR1 and RBR2 were modified to generate strains encoding tagged RBRs. Both tagged RBRs identified Fd3, although in varying redox states (Fig. 6A; Fig. S11; Tables S19 and S20), suggesting that RBRs may play a role in maintaining the redox environment of Fd3. RBR1 was identified by every tagged VOR subunit, RBR2 was identified by every tagged POR subunit, and RBR3 was identified by each of the three tagged OGOR subunits (Fig. 6B), suggesting that RBRs are important in amino acid catabolism and that specific catabolic complexes associate preferentially with specific RBRs. While the functional role(s) of RBRs is not defined, it is plausible that RBRs are incorporated into key redox complexes as a means of oxidative stress protection.

## DISCUSSION

While *in vitro* investigations of enzyme activities reveal key parameters under defined conditions, *in vitro* studies alone often fail to reveal dynamic, regulatory, and key protein–protein interactions. Efforts to define large networks of protein–protein partnerships have been completed for some model species (35–37, 57); however, efforts to establish protein–protein partnerships in Archaea—and particularly hyperthermophilic archaea with biotechnological and biofuel potential—are rare (8, 21). Establishing the protein networks that coordinate the bulk of redox metabolism in the model archaeon, *T. kodakarensis*, via reproducible and statistically supported AP-MS analyses, provides insights into the dynamic and complex *in vivo* protein landscape that ultimately determines energy production and energy conservation. The 25 genes included in this study encode metabolic enzymes composing ~10 functional complexes performing catabolic and anabolic activities (Fig. S1). GAPOR (Fig. S12; Table S21) and POR are active in glycolysis; VOR, IOR, and OGOR function in amino acid fermentation pathways; and FDH further harnesses electrons from acid oxidation. Anabolic activities include the generation of reduced small molecules by FNOR1 and FNOR2 (Fig. S13 and S14; Tables S22 to S25), lipid maturation catalyzed by GGR, and the MBS respiratory complex. All 25 target genes were modified at their native genomic loci to generate tagged proteins at native expression levels under two redox states, allowing for identification of novel partnerships, refining subunit associations for previously *in vitro*-characterized protein complexes, and revealing an interconnected and dynamic landscape controlling electron transport and energy conservation. The resulting data suggest that each tagged protein maintains an average of ~30 interacting partners, which is highly interconnected compared to a model mesophilic eukaryote that has been shown to display about half of the number of interactions (57). Of the 436 proteins identified as significant interacting partners within the metabolic interactome of *T. kodakarensis*, 151 (~1/3) proteins were only observed within a single complex, whereas the bulk of proteins identified (284; ~2/3) were observed in at least two, and often many more assemblies, supporting a mixed and likely dynamic population of *in vivo* assemblies controlling energy generation and conservation.

Identification of reproducibly and reciprocally identified *in vivo* protein assemblies provides a contrasting, yet complementary view to the bulk of *in vitro* protein characterization studies. Multiple proteins and protein complexes were identified with greater or fewer reproducibly recovered *in vivo* interaction partners in one redox state over another: IOR, RBR1, FNOR1, FDHα, and FDHγ_4_ all maintained more than double the number of protein associations when S˚ was present, while FNOR2, FDHγ_2_, and FDHγ_3_ maintained more than double the number of protein associations in the absence of S˚ (Fig. 1B), which support previously identified redox trends (49). Our *in vivo* AP-MS data support an overarching profile of dynamic and subunit-interchangeable assemblies for many of the major redox pathways in the central metabolism of *T. kodakarensis* that may subtly or even radically impact enzymatic functions and overall energy dynamics in cells. While caution is warranted in extending our results beyond our model species, the diverse and dynamic environments that support the growth of many archaea may demand fitness-relevant changes in the composition of redox active assemblages to maximize energy gains under diverse conditions, and it is plausible that many of the compositional and partnership changes observed for redox associations in this study would be valuable for species within many clades.

The dynamic changes to *in vivo* assemblages are highlighted by examination of POR, originally classified as a heterotrimeric pyruvate-catabolizing complex (30) that functions *in vitro* without the inclusion of a fourth, non-operonic subunit (TK1978), which was only biochemically identified later (13, 41). *In vivo* AP-MS data support the formation of a stable, stand-alone tetrameric POR assemblage in the presence of S˚. Likewise, VOR appears to be a stand-alone tetramer in the presence of S˚ (Fig. 3C and D), but interestingly, a rather dramatic association of POR with the tetrameric VOR complex—that share a common subunit (TK1978)—appears to be adopted when the preferred terminal electron acceptor (S˚) is absent. Similarly, OGOR had been suggested to function as a heterooctamer (13) and heterotetramer (27), and our AP-MS data suggest that OGOR_1_ and OGOR_2_ tetramers may separate in the presence of S˚ and associate in the absence of S˚ (Fig. 4A). While such assertions require biochemical validation and characterization to establish the impacts of larger complex formation, it appears that the tetrameric or octameric nature of these catabolic complexes *in vivo* may provide a novel strategy to dynamically respond to specific redox states, suggesting that multiple protein associations respond to the presence or absence of varying terminal electron acceptors.

Prior studies established that PECs (specifically Fds) provide selective routes to coordinate electron flux (8), and thus mapping protein–Fd associations with larger redox assemblies was of immediate interest. While a single tryptic fragment of Fd2 was detectable in association with GGR and thus fully congruent with prior work (26, 52), evidence of Fd1 within assemblages was technically limited. Despite varied purification schemes, different sample handling techniques, different LC-MS-MS instrumentations, as well as using multiple detection methods, only a single peptide unique to Fd1 was identified when employing data-dependent acquisition (DDA). Thus, while the Fds are known to selectively shuttle electrons from select donors to specific acceptors, the nature of the tryptic fragments generated from these small PECs was largely not amenable to the techniques employed to identify the complexity of the bulk of the redox assemblies.

The totality of the voluminous AP-MS data collected within this study is reported in large supplemental data sets (including intersecting results, which can shed light on more dynamic network hubs, such as that seen with MBS, OGOR, and FDH [Data Set S1]). While a detailed description of these data is not feasible within the confines of a single report, the interconnections of electron flux—toward major sinks of membrane potentials generated through MBS and MBH (with associated production of H_2_S and H_2_, respectively), lipid reduction through the activities of geranylgeranyl reductase and Fd2, and regeneration of small molecular redox carriers via FNORs—are repeatedly reinforced by the assemblages and dynamic changes to such in the presence and absence of S˚. Our results demonstrate that electron flux responds to environmental conditions and can likely be tuned to funnel more electrons to specific endpoints but only with concomitant changes to the major redox protein assemblies *in vivo*. It will be critical to establish how changes to the composition of redox active protein assemblages are coordinated, and how complexes composed of different subunits behave with respect to each other in shuttling electrons. Only with a more complete knowledge of how electron flux is regulated and controlled can rational engineering release the full biotechnological value of archaeal biochemistries.

## MATERIALS AND METHODS

### Media formulations, culture growth, and *T. kodakarensis* strain construction

Parental (TS559) (23) and markerlessly modified strains of *T. kodakarensis* were anaerobically grown from 1:100 inoculums, as previously described (8, 20, 21), at 85°C in an artificial sea water base media supplemented with 5 g/L each of yeast extract and tryptone, and when appropriate, 2 and 5 g/L of S˚ and pyruvate, respectively, were added. Strain TS559 served as the parental strain for a two-stage integration and excision strain construction process, as previously described (19, 20, 58), wherein individual native loci were markerlessly modified to introduce 45 bp to the genome, with 27 bp (TACCCATACGACGTTCCGGACTACGCA) encoding a codon-optimized nine amino acid hemagglutinin (HA) epitope tag and the remaining 18 bp (CATCACCATCACCATCAC) encoding a six histidine affinity tag. Chromosomal markerless allelic exchange techniques result in minimal perturbation of native expression levels of target proteins within *T. kodakarensis*. When the open reading frame of a target protein encoding gene overlapped with an adjacent gene, the overlapping sequences were duplicated to eliminate concerns of the tagged encoding sequences impacting the production of proteins from adjacent genes (Fig. S15). When the introduction of the epitope and affinity tag encoding sequences to the C-terminus of target proteins proved incompatible with strain survival, the His_6_ and HA encoding sequences were instead introduced immediately following the native start codon to generate a protein with N-terminal extensions, which was required for only two genes included in this study: TK1129 and TK1130.

The genomic sequences of all modified loci were first confirmed via amplification of the modified locus by PCR; addition of the 45 bp to the genome resulted in a larger amplicon in modified versus parental strains (Fig. S2C). A secondary confirmation of the addition of the epitope and affinity tag encoding sequences was provided by differential *Bsp*EI digestion of amplicons from modified and parental strains (Fig. S2B). A tertiary measure of modification of the target loci was target loci amplicon generation via PCR and amplicon sequencing. Finally, to ensure our genetic manipulations did not introduce undesirable modifications anywhere within the >2 Mbp genome (10), we prepared DNA and sequenced the whole genome of every strain via in-house or commercial Oxford Nanopore Technology (ONT) sequencing at minimally 30× coverage and often >1,000× coverage (Fig. S2D through F).

### Purification of *in vivo* protein assemblies

*T. kodakarensis* biomass destined for AP-MS was rapidly chilled in an ice-water bath and harvested (9,000 × *g* for 20 minutes at 4°C) from cultures grown to mid-exponential phase (OD_600 nm_ 0.4–0.7) at 85°C. Cell pellets were stored at −80°C. To maintain the greatest number of protein interactions, all lysis and downstream chromatographic steps were completed as rapidly as feasible (typically in <4 hours). A minimum of three biological replicates were performed for each target protein to provide statistically relevant and reproducible results for every strain. Harvested biomass was resuspended in 3 mL of 10 mM Tris HCl (pH 8.0) per gram of cells, 500 mM NaCl, and 10% wt/vol glycerol (buffer A), repeatedly sonicated on ice. A clarified cell lysate was collected following centrifugation (9,000 × *g*, 20 minutes, 4°C). The clarified cell lysate was further clarified by passage through a 0.45 µm sterile filter immediately prior to chromatographic separations on a Ni^2+^-charged 5 mL chelating column (Cytiva Life Sciences, 17040903). One tagged protein, GAPOR (TK2163), required affinity purification using a Cu^2+^-charged column, and respective controls were purified in like fashion. After the capturing of tagged proteins and their binding partners to the column matrix and exhaustively washing the column with buffer A, 10 mL (or two column volumes) of 5% buffer B (10 mM Tris HCl [pH 8.0], 100 mM NaCl, 10% wt/vol glycerol, 200 mM imidazole) was used to wash the column, followed by collecting 1 mL fractions over a linear elution profile from 5% to 100% buffer B over ~40 mL (or four column volumes) to capture assemblages based on the tagged target protein (Fig. S3A).

Purification efficacy was assessed by SDS-PAGE and Coomassie or silver staining and by Western blotting based on the identification of the HA tag (Fig. S3B through E). An SDS-PAGE 4%–20% Criterion TGX Stain-Free gel was visualized under UV light to identify proteins containing tryptophan residues. Typically, the tagged protein was not visible at the detection level of the Stain-Free system (Fig. S3B). Denatured proteins run on a 12% Tris-Glycine gel were stained with Coomassie blue dye, which binds to amino acids with primary amines, and repeated methanol rinses removed sufficient stain to weakly visualize low concentrations of purified, tagged protein (Fig. S3C). Silver staining of denatured proteins separated on a 12% Tris-Glycine gel provided exceptional sensitivity to visualize proteins at low concentrations and was able to identify the purified, tagged protein along with other proteins that co-elute (Fig. S3D). Denatured proteins separated by SDS-PAGE were transferred to 0.2 µm polyvinylidene fluoride (PVDF) membrane and were visualized via Western blotting employing 1:10,000 anti-HA 1˚ antibody (Invitrogen, 26183-1MG) made in mouse, which binds to the HA epitope encoded on tagged protein, and a 1:1,000 anti-mouse conjugated alkaline phosphatase 2˚ antibody (LGC Clinical Diagnostics, 5220-0312) was used for colorimetric detection (Fig. S3E).

Sufficient volumes (typically ~0.5 mL) of ~3–5 central fractions containing the HA-tagged target protein of interest were pooled (Fig. S3E, red box), such that ~20 µg of total protein could be precipitated on ice by the addition of 0.25 volumes of 100% (wt/vol) trichloroacetic acid (TCA) and protein recovery via centrifugation (15,000 × *g*, 5 minutes, 4°C). Following the removal of the supernatant, the protein pellet was washed twice with 200 µL chilled acetone and then dried at 95°C for 5 minutes. Precipitated protein pellets were resuspended and incubated in 100 µL of 50 mM ammonium bicarbonate at 65°C for 15 minutes after the addition of 5 µL of 5 µg/µL DTT in 50 mM ammonium bicarbonate, followed by the addition of 5 µL of iodoacetamide (15 mg/mL in 50 mM ammonium bicarbonate) prior to a 30 minute room temperature dark incubation. Sequencing grade-modified trypsin (Promega, Madison, WI, USA) prepared in 50 mM ammonium bicarbonate was added to the sample at a 1:20 enzyme-substrate ratio for ~12 hours of 37°C incubation prior to quenching with 0.2% acetic acid (50 µL) for acidification, evaporative drying, and final resuspension with 40 µL of 0.1% formic acid.

### Liquid chromatography and tandem mass spectrometry

Liquid chromatography-nanospray tandem mass spectrometry (LC/MS/MS) was performed on a Thermo Scientific Orbitrap Fusion mass spectrometer equipped with an EASY-Spray Sources operated in positive ion mode. Samples (~1 µg in 2 µL) were separated on an easy spray nano column (Pepmap RSLC, C18 3µ 100A, 75 µm × 250 mm Thermo Scientific) using a 2D RSLC HPLC system from Thermo Scientific. Each sample was injected into the µ-Precolumn Cartridge (Thermo Scientific) and desalted with 0.1% formic acid in water for 5 minutes. The injector port was then switched to injection mode, and the peptides were eluted off of the trap onto the column. Mobile phase A was 0.1% formic acid in water, and acetonitrile (with 0.1% formic acid) was used as mobile phase B. The flow rate was set at 300 nL/minute. Mobile phase B was first kept at 2% for 5 minutes and then increased from 2% to 16% in 50 minutes before being increased again from 16% to 25% over 10 minutes. Mobile phase B was finally increased again from 25% to 85% in 1 minute and then kept at 85% for another 1 minute before dropping back to 2% in 1 minute. The column was equilibrated at 2% of mobile phase B (or 98% A) for 12 minutes prior to the next sample injection. MS/MS data were acquired with a spray voltage of 1.6 kV with a capillary temperature of 305°C. The scan sequence of the mass spectrometer was based on the preview mode data-dependent TopSpeed method: the analysis was programmed for a full scan recorded between *m/z* 200–2,000 and an MS/MS scan to generate product ion spectra to determine the amino acid sequence in consecutive scans starting from the most abundant peaks in the spectrum in the next 3 seconds. To achieve a high mass accuracy MS determination, the full scan was performed in Fourier transform (FT) mode, and the resolution was set at 120,000 with internal mass calibration. The Automatic Gain Control Target ion number for FT full scan was set at 4 × 10^5^ ions, the maximum ion injection time was set at 50 ms, and the micro scan number was set at 1. MSn was performed using higher-energy collisional dissociation in ion trap mode to ensure the highest signal intensity of MSn spectra. The higher-energy collisional dissociation collision energy was set at 32%. The Automatic Gain Control Target ion number for ion trap MSn scan was set at 3.0E4 ions, the maximum ion injection time was set at 35 ms, and the micro scan number was set at 1. Dynamic exclusion was enabled with a repeat count of 1 within 20 seconds and a low mass width and a high mass width of 10 ppm.

Data were searched using the Mascot Daemon by Matrix Science version 2.7.0 (Boston, MA, USA) via Proteome Discoverer (version 2.4, Thermo Scientific), and the database was searched against the most recent Uniprot databases: Common_Contamination_Proteins-cRAP_LZ_20190813.fasta (118 entries); Sere_TK1694_20220627.fasta (one entry); Sere_TK1694_010323_20230103.fasta (12 entries); and Uniprot_Thermococcus_Kodakarensis_KOD1_030121_20210301 database (unknown version, 2,432 entries). The mass accuracy of the precursor ions was set to 10 ppm, including an accidental pick of 1 ^13^C peak (to allow for the natural but random incorporation of heavy carbon). The fragment mass tolerance was set to 0.5 Da. Carbamidomethylation (Cys) was used as a fixed modification, and we considered variable modifications including oxidation (Met) and deamidation (N and Q). Four missed cleavages for the enzyme were permitted, which allowed for proteins from underdigested samples to still be detected, while most proteins only contained 0–2 missed cleavages. Using the empirical null approach, a decoy database was searched to determine the false discovery rate (FDR), and peptides and proteins were filtered at 1% FDR. Proteins were identified with a minimum of at least one unique peptide.

### Statistical analysis of proteomic MS data

Mean spectral counts assigned to each *T. kodakarensis* protein identified within experimental (fractions recovered from tagged strains) and control (identical chromatographic fractions recovered from otherwise isogenic parental strains) samples were individually compared (Significant_Copurifying_Proteins.xlsx). The sensitivity of our LC-MS-MS techniques typically positively identified ~100–700 proteins per sample, and we applied a Fisher’s exact test (*P* = 0.05) with a Benjamini-Hochberg multiple testing correction and a minimum log_2_ fold change of 2 to consider the abundance of proteins in experimental samples significantly greater than control samples. Significant proteins were identified from comparisons involving all experimental and all control replicates that typically included a minimum of six sample runs (three experimental and three control) (Fig. 1A).

Significance as measured by the Fisher’s exact test for every protein in a comparison returns a *P* value and log_2_ fold change value (Fig. 1A). The *P* value provides a quantitative metric for the likelihood of observing the difference in spectral counts of a protein between sample conditions (experimental vs control); *P* values are typically plotted as the -log_10_ conversion of the *P* value on a *y*-axis (i.e., -log_10_[0.05] = 1.3, or -log_10_[0.01] = 2). The complementary log_2_ fold change value plotted on the *x*-axis measures the difference in abundance of a protein between the experimental and control samples. We mandated that a protein returns a *P* value of <0.05 and a log_2_ fold change ≥2 in experimental over control conditions to be considered significant. After determining standard statistical parameters, only one target protein, the product of TK2076, was narrowly excluded from data sets (typically displaying a log_2_ fold change of ~1.8). In the rare instances that the tagged protein fell just outside of the threshold of significance, the threshold for abundance in experimental samples was lowered to include the tagged protein and all proteins within this adjusted range in the respective sample comparison; thus, TK2076C was rescued when appropriate.

### A simplified ranking system to define the reproducibility and confidence of protein copurifications

Our stringent thresholds for reliably identified proteins eliminate false positives and provide a platform to quantize the confidence of positive identifications (Fig. 1A; Data Set S2). A five-tier ranking system (5 = highest confidence; 1 = less confidence) compiles data from each of the three experimental biological replicates as well as the *P* value and the log_2_ fold change. While a minimum threshold *P* value of <0.05 was required for a positive identification, a *P* value of <0.01 provides increased confidence that the protein was not identified as significant by chance, increasing the ranking of such an identification. Similarly, while a minimum log_2_ fold change ≥2 in protein abundance must be met, a protein identified with a log_2_ fold change ≥4 increases confidence that this protein is more abundant in the experimental samples compared to the control samples and results in an increased score within the ranking. The remaining component of our ranking system considers the reproducibility of protein identifications between biological replicates. A protein that appears in three biological replicate purifications at a *P* value ≤ 0.01 and a log_2_ fold change of ≥4 earns a rank score of 5 (Fig. 1A). A protein that falls short in any one category would earn a rank score of 4, etc.

## Data Availability

LC-MS-MS data (“.dat” files exported in “.xlsx” format; ~86.1 MB of data in 178 files) of peptides mapping to proteins as determined by MASCOT via Proteome Discoverer are available at https://osf.io/8b2es/?view_only=aa1d17e3bba64d3685eeea6a3c2a2aa7. Spectral counts for significant copurifying proteins can be found in Data Set S2. Analyses of overlapping or intersecting protein partners for individually tagged proteins and their complexes can be found in Data Set S1.

## SUPPLEMENTAL MATERIAL

Data Set S1 aem.00293-25-s0001.xlsx Intersecting significant proteins.

Data Set S2 aem.00293-25-s0002.xlsx Significant copurifying proteins.

Supplemental figures aem.00293-25-s0003.pdf Figures S1 to S15.

Supplemental tables aem.00293-25-s0004.pdf Tables S1 to S25.

ASM does not own the copyrights to Supplemental Material that may be linked to, or accessed through, an article. The authors have granted ASM a non-exclusive, world-wide license to publish the Supplemental Material files. Please contact the corresponding author directly for reuse.
