# Supplementary material for: A dynamic protein interactome drives energy conservation and electron flux in Thermococcus kodakarensis
Source: Appl Environ Microbiol. 2025 Apr 3;91(4):e00293-25. doi: 10.1128/aem.00293-25 (PMC12016516; doi:10.1128/aem.00293-25)
Supplement: Supplemental figures — Figures S1 to S15. [file aem.00293-25-s0003.pdf]

SUPPLEMENTAL FIGURES

A dynamic protein interactome drives energy conservation and electron flux in *Thermococcus kodakarensis*

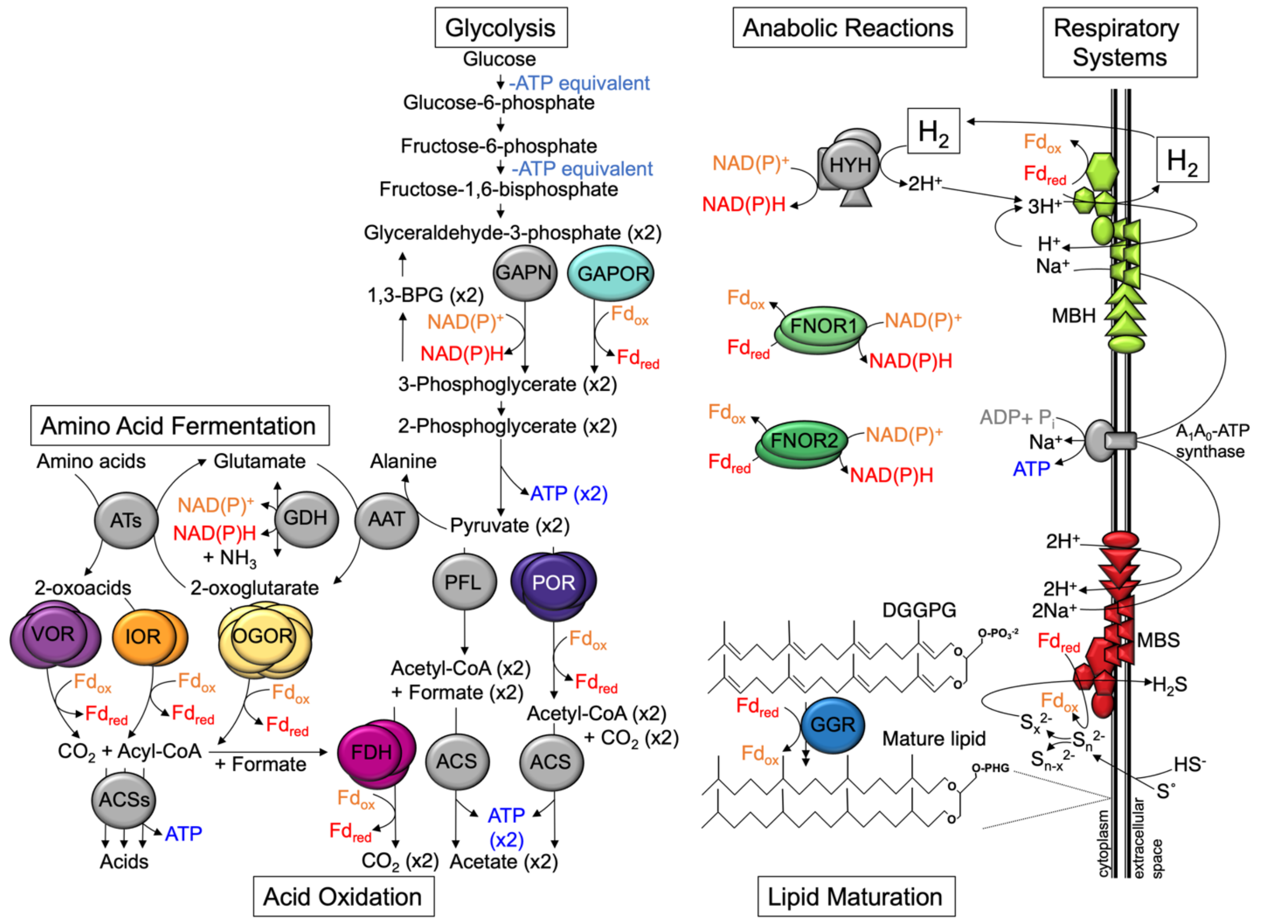

Figure S1: ***T. kodakarensis* catabolic and anabolic pathways provide for energy consumption and generation via diverse pathways.** Catabolic pathways (at left) include amino acid fermentation wherein amino acids are deaminated to generate 2-oxoacids. Three unique protein complexes, VOR, IOR, and OGOR (plum, orange, yellow), further catabolize 2-oxoacids into acyl-Co-A and CO_2, generating a reduced PEC (*e.g.*, Fd_{red}) in the process. *T. kodakarensis* maintains both glycolytic and gluconeogenic capacities via a modified Embden-Meyerhof-Parnas pathway (center left) that results in the net gain of 2 ATP (or equivalent) upon the conversion of glucose to pyruvate (x2). Pyruvate can further be oxidized by POR (purple) to produce Fd_{red} and acetyl-CoA. Acyl-CoA and formate are oxidized to CO_2 by FDH (fuchsia) generating

another Fd_{red}. Fd_{red} generated in catabolic reactions are utilized in a variety of anabolic reactions (center right). FNOR1 and FNOR2 (greens at center) oxidize Fd_{red} to generate reduced small molecules (*e.g.*, NADPH) and GGR (blue at bottom) catalyzes the maturation of isoprenoid lipids. Two membrane-bound respiratory systems, MBH (lime green at top right) and MBS (red at bottom right), generate a proton gradient at the membrane. In the presence of S˚, MBS catalyzes the reduction of disulfides to generate a proton gradient, while in the absence of S˚, MBH reduces protons towards the production of H_2 gas. Each respiratory complex maintains a Na^+/H^+ antiporter which exchange the protons for Na^+, thereby driving A_1A_0-ATP synthase and producing ATP. Proteins tagged in this study are shown in various colors while additional metabolic proteins of interest are shown in grey. Figure adapted from Burkhart *et al.*, 2019.

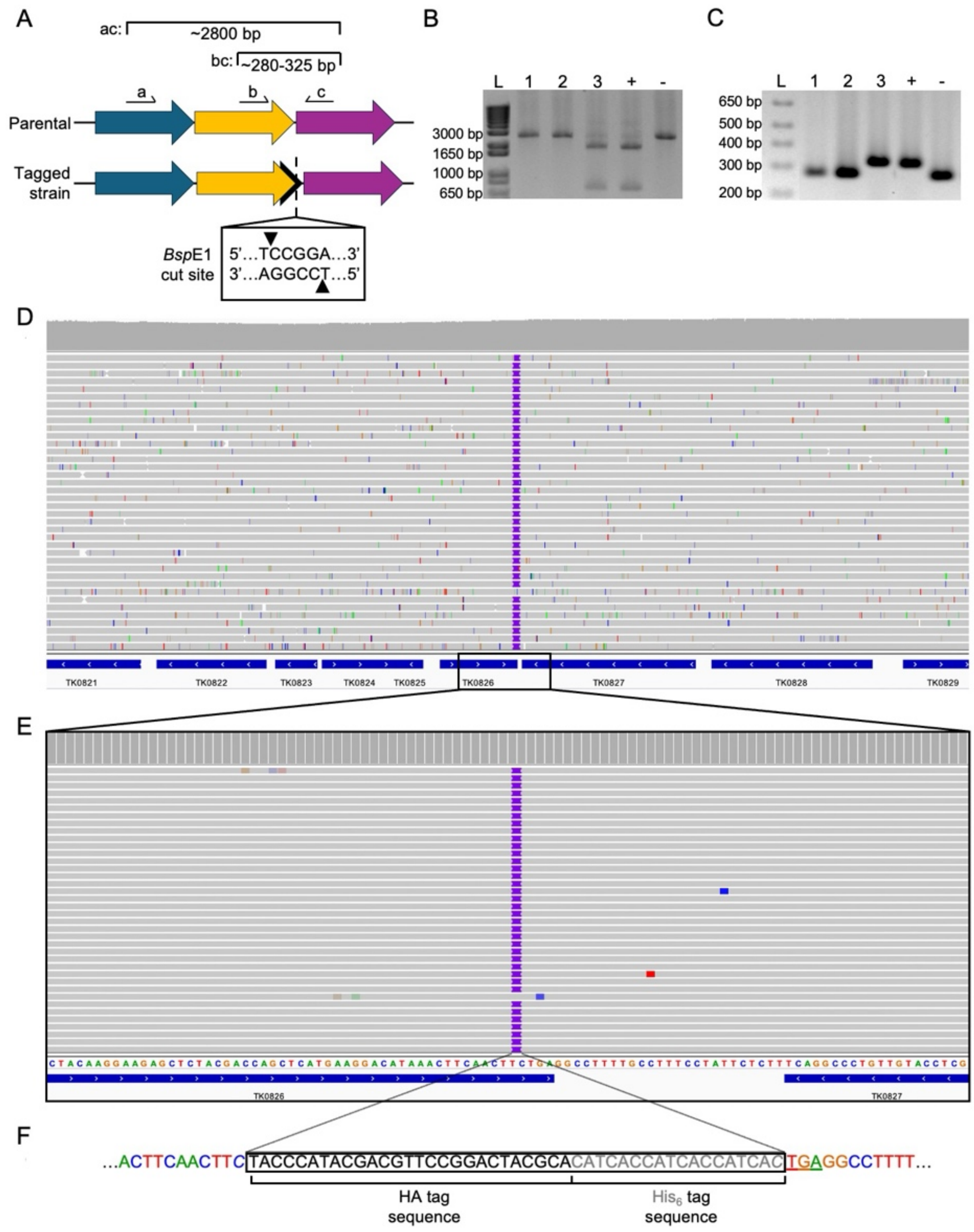

Figure S2: Confirmation of the presence or absence of the tag sequence in transformed genomic DNA via PCR and Whole Genome Sequencing (WGS). A)

Genomic sequences are modified to encode a hemagglutinin (HA) and His$_6$ tag sequence: 5'-TACCCATACGACGTTCCGGACTACGCACATCACCATCACCATCAC-3'. The *Bsp*E1 restriction enzyme cleaves following the T in the 5'-TCCGGA-3' sequence included in the HA tag sequence. Primers upstream ("a", "b") and downstream ("c") of the modification are used to amplify DNA purified from transformed strains following pop-in/pop-out selection. **B)** Amplicons generated via PCR with "a" and "c" primers that flank the target locus using genomic DNA purified from putatively modified strains of *T. kodakarensis* are digested with *Bsp*EI prior to resolution via agarose gel electrophoresis to identify the genotype of interest. Amplicons generated from DNA purified from parental, non-modified strains (lanes 1, 2, and -) are resistant to *Bsp*E1 digestion, whereas amplicons generated from DNA purified from strains encoding the HA and His$_6$ tags (lanes 3 and +) now encode a *Bsp*EI recognition site within the newly added sequences encoding the epitope and affinity tags. **C)** An alternative diagnostic PCR strategy employing purified genomic DNA and "b" and "c" primers is used to identify genotypes lacking (lanes 1, 2, and -) or containing (lanes 3 and +) the 45 bp insertion amplified in a short sequence (< 400 bp) encoding the HA and His$_6$ sequences can immediately reveal the migration impact on amplicon size due to the genomic insertion. **D)** Whole genome sequencing of *T. kodakarensis* strains confirms the absence of secondary site modifications throughout the genome while confirming the exact sequence of the desired genomic modifications encoding the epitope and affinity tag sequence insertion at the target locus. **D** and **E)** Coarse and zoomed, respectively, IGV views of binary alignment maps (.bam) files from DNA extracted from a strain with DNA sequences encoding an HA and His$_6$ tag preceding the stop codon of TK0826. Standard

IGV settings identify reads horizontally as grey bars and depth of coverage by stacking reads vertically (>700X coverage recovered, although only ~40X coverage is displayed). Open reading frames are identified in blue below the sequencing reads and insertions are identified as purple bars within the grey reads. **F)** The inserted sequences encode the HA epitope (black, bold) and six histidine affinity tag (gray) sequences immediately upstream of the TGA translation stop codon (underlined).

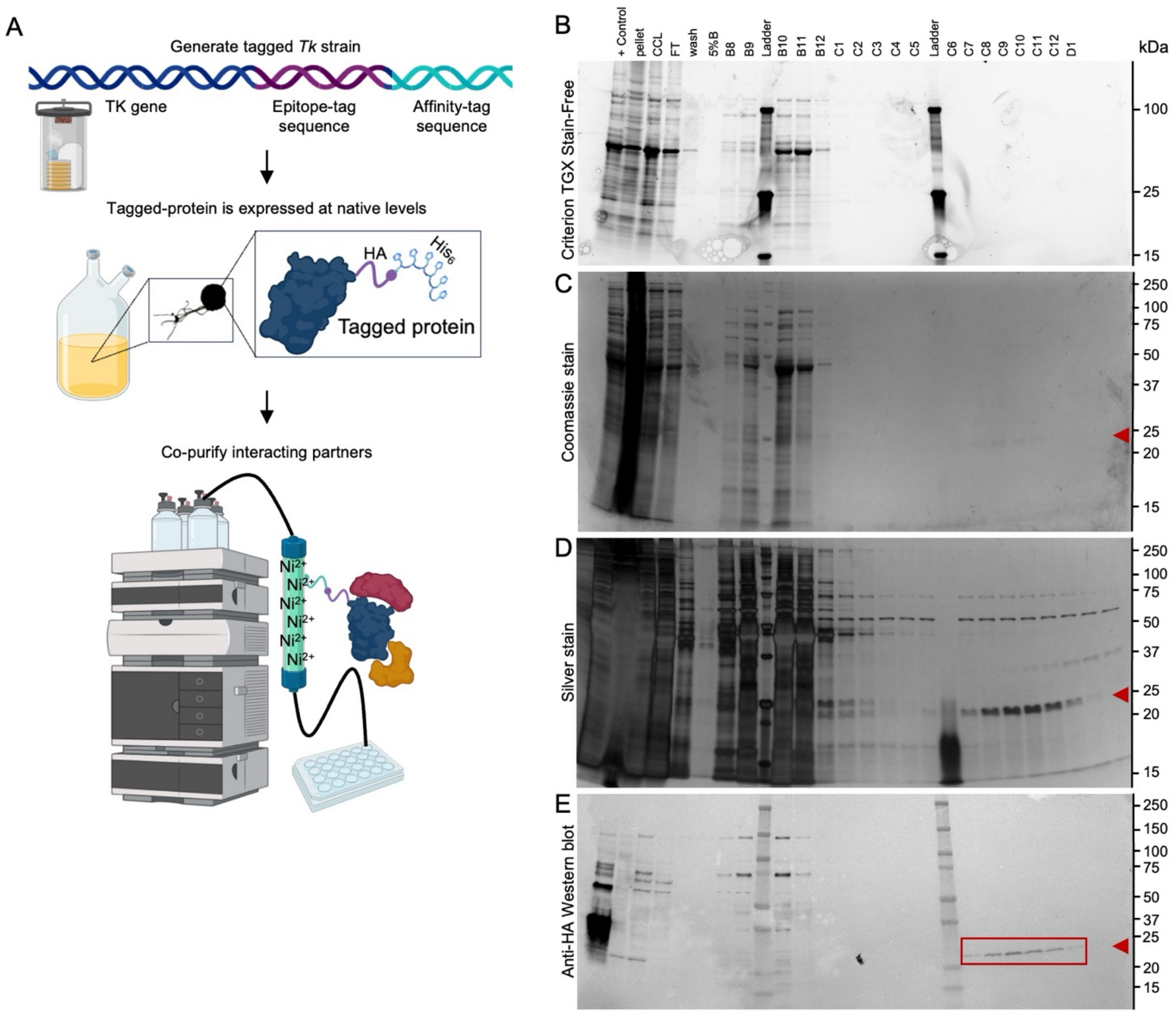

Figure S3: AP-MS identifies co-purified proteins associating with the tagged protein at native expression levels. A) *T. kodakarensis* strains are modified to encode a genomic epitope and affinity tag at the end of the gene sequence using targeted mutagenesis and selection strategies. Sequence confirmed strains are cultured to produce biomass in minimally three biological replicates. Actively dividing cells are pelleted before being lysed via sonication and clarified via centrifugation. The clarified cell lysate (CCL) is purified over a prepared Ni^{2+}-charged affinity column. **B)** Proteins of interest within chromatographic fractions separated by polyacrylamide gel

electrophoresis are typically not sufficiently abundant for identification through stain-free imaging **(B)** or Coomassie staining **(C)** but are often sufficiently abundant to be identified through silver staining **(D)** or anti-HA Western blotting **(E)**. In this representative example, the target protein of interest is the 22.6 kDa product of tagged-TK0826 is marked on the right side by a small, red triangle **(C-E)**. Three-six central fractions containing the tagged-protein are pooled for analysis (**E**, red box). CCL from a *T. kodakarensis* strain encoding a tagged-TK1984 (36 kDa) in lane 1 serves as the positive control. Precision Plus Dual Color Standard protein ladder (BioRad, 1610374) was loaded in lane 9 on **(B-E)** and lane 18 on **(B)** and **(E)**. No sample was loaded in lane 18 in **(C)** and 1 μg of recombinant-TK1694 (~7 kDa) was loaded in lane 18 in **(D)**. Figure partially created in BioRender.com.

A

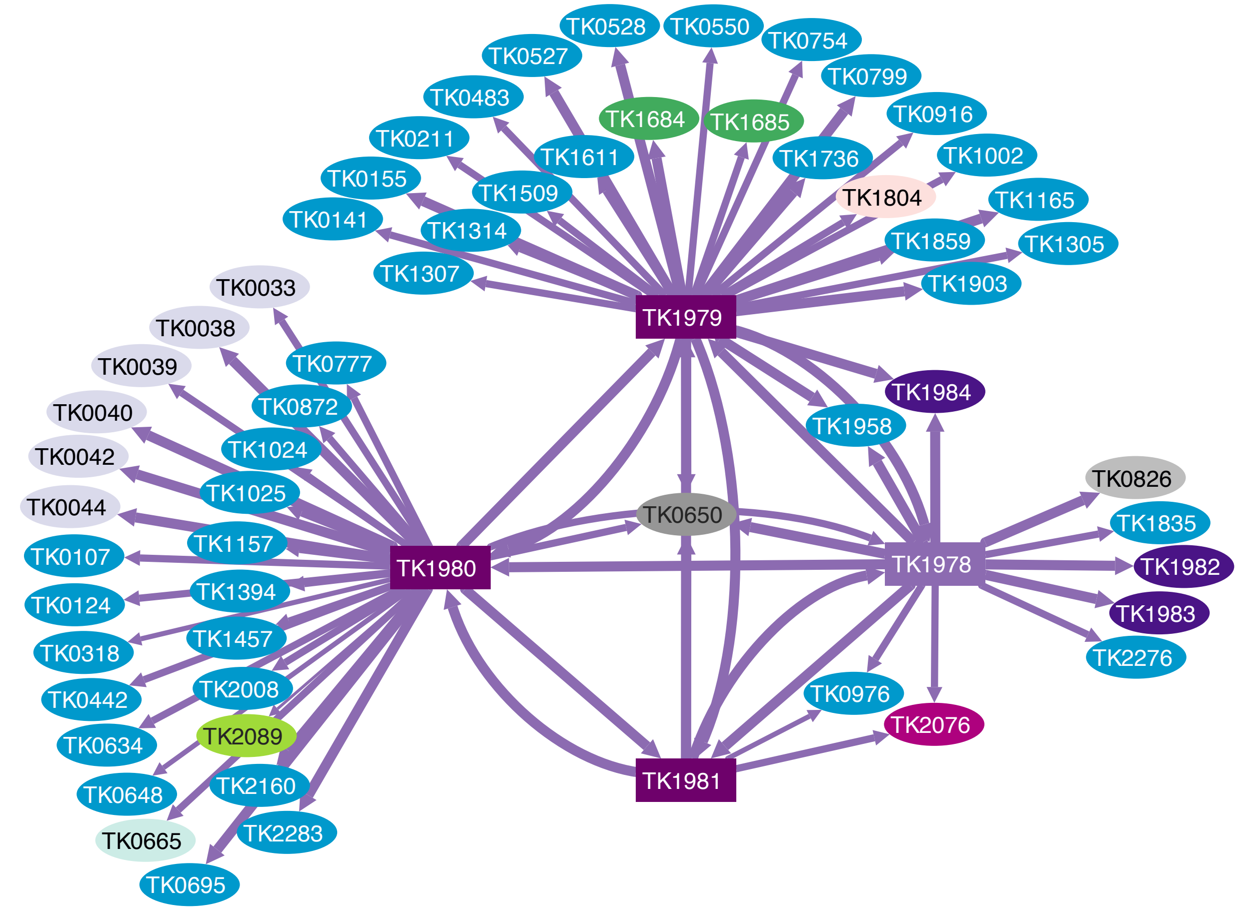
TK0528
TK0550
TK0754
TK0527
TK0799
TK0483
TK1684
TK1685
TK0916
TK0211
TK1611
TK1736
TK1002
TK0155
TK1509
TK1804
TK1165
TK0141
TK1314
TK1859
TK1305
TK1307
TK1903
TK0033
TK1979
TK0038
TK0777
TK0039
TK1984
TK0872
TK0040
TK1958
TK1024
TK0042
TK0826
TK1025
TK0650
TK0044
TK1835
TK1157
TK0107
TK1980
TK1978
TK1982
TK1394
TK0124
TK1983
TK1457
TK0318
TK2276
TK2008
TK0442
TK0976
TK2076
TK2089
TK0634
TK1981
TK2160
TK0648
TK2283
TK0665
TK0695

B

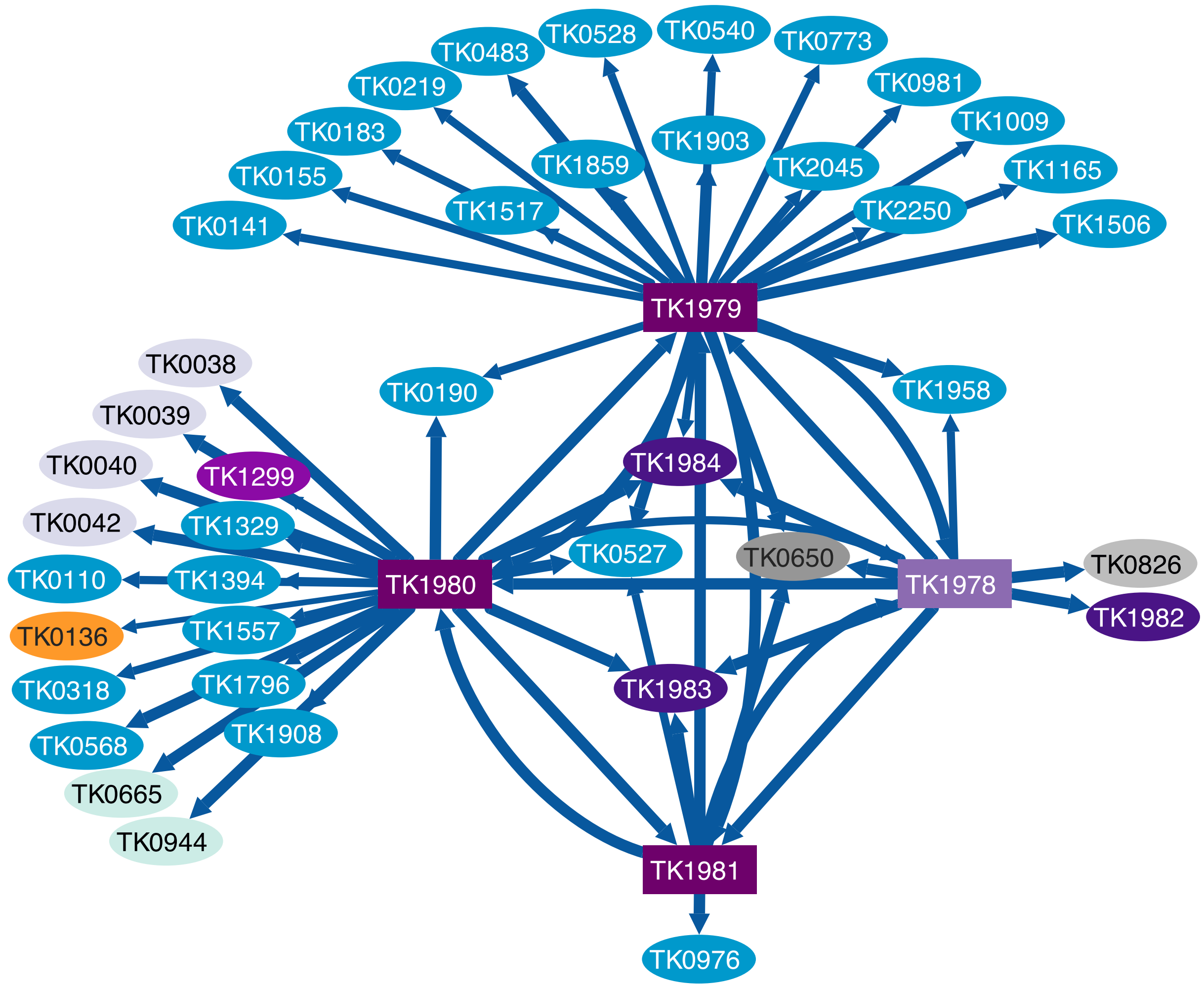

Figure S4: Co-purifying proteins of the VOR complex. The VOR heterotetramer is composed of TK1978 – 1981 and all four subunits were independently tagged for AP-MS analysis. Each tagged-subunit of VOR identified every other VOR-subunit with high confidence in +S˚ **(A)** and -S˚ **(B)** conditions. Similar numbers of significant co-purifying proteins were seen in both redox states. All POR subunits (TK1982-1984, dark purple) were identified in both conditions. TK0650 (RBR1) was identified by all VOR subunits in +S˚ and ¾ subunits in -S˚.

A

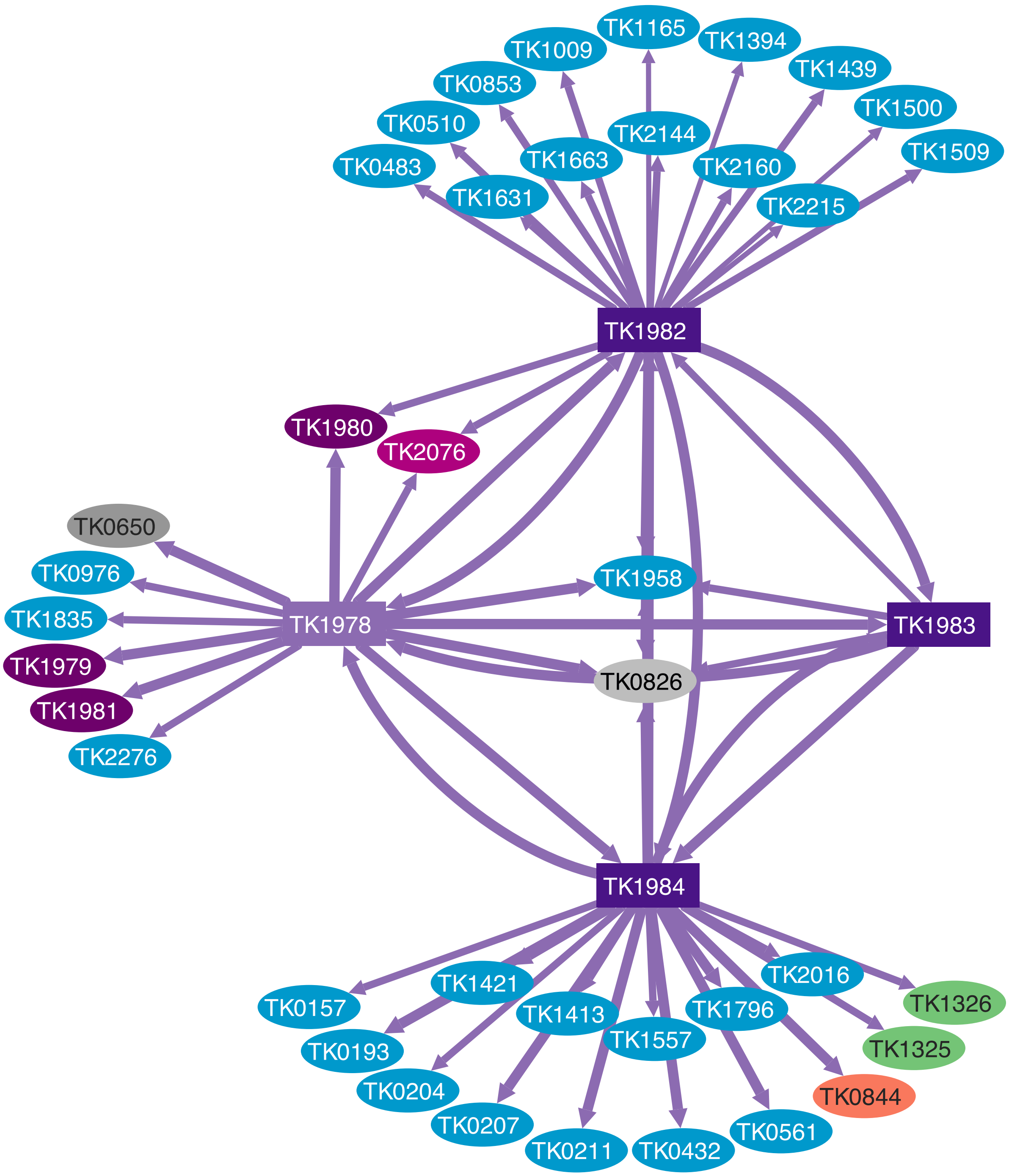
TK1165
TK1009
TK1394
TK0853
TK1439
TK1500
TK0510
TK2144
TK1509
TK1663
TK0483
TK2160
TK1631
TK2215
TK1982
TK1980
TK2076
TK0650
TK0976
TK1958
TK1835
TK1978
TK1983
TK1979
TK0826
TK1981
TK2276
TK1984
TK2016
TK1421
TK1326
TK0157
TK1413
TK1796
TK1557
TK0193
TK1325
TK0204
TK0844
TK0207
TK0561
TK0211
TK0432

B

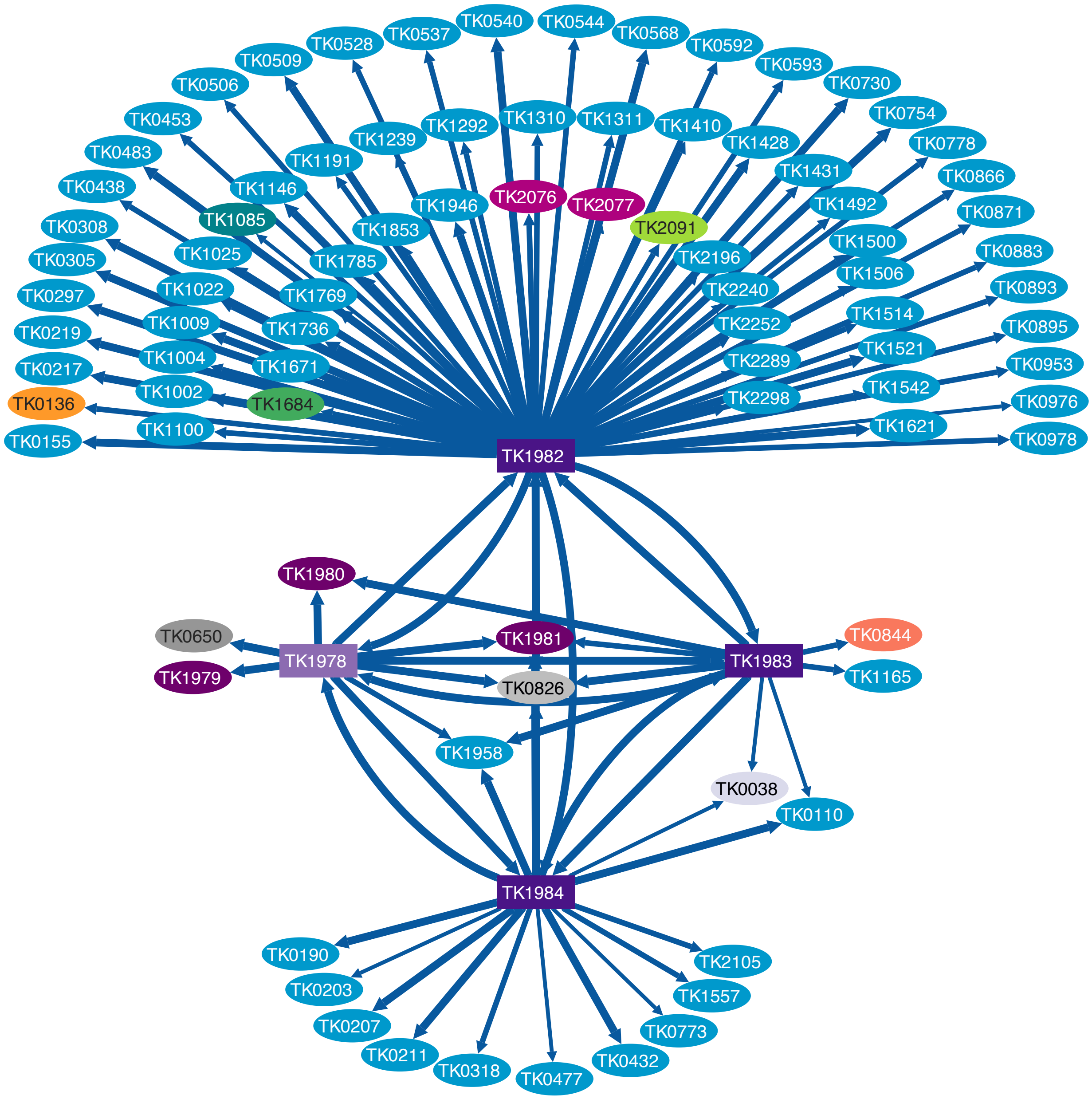

Figure S5: Co-purifying proteins of the POR complex. The POR heterotetramer is composed of TK1978 and TK1982-1984 and all four subunits were independently tagged for AP-MS analysis. Each tagged-subunit of POR identified every other POR-subunit with high confidence in +S˚ **(A)** and -S˚ **(B)** conditions. All VOR subunits (TK1978-1981, plum) were identified in both conditions. TK0826 (RBR2) was identified by all VOR subunits in both conditions.

A

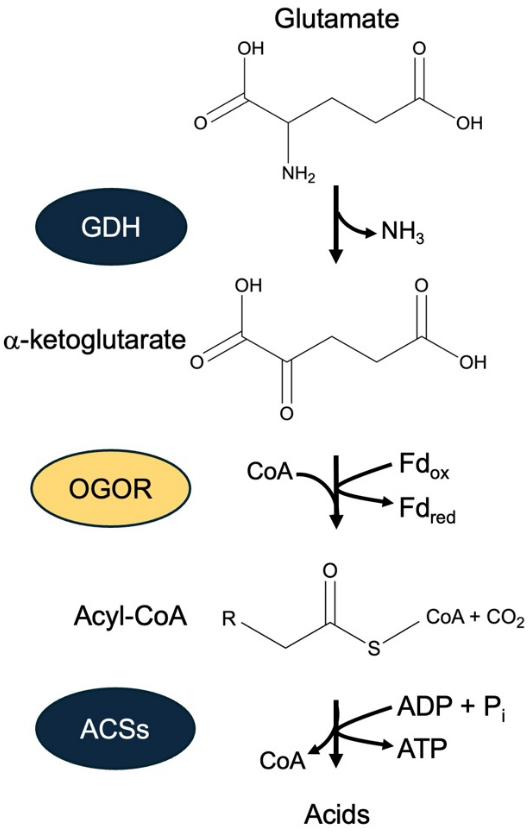

Glutamate
OH
O
O
OH
NH2
GDH
NH3
OH
O
α-ketoglutarate
O
OH
O
OGOR
CoA
Fdox
Fdred
O
Acyl-CoA
R
S
CoA + CO2
ACSs
ADP + Pi
ATP
CoA
Acids

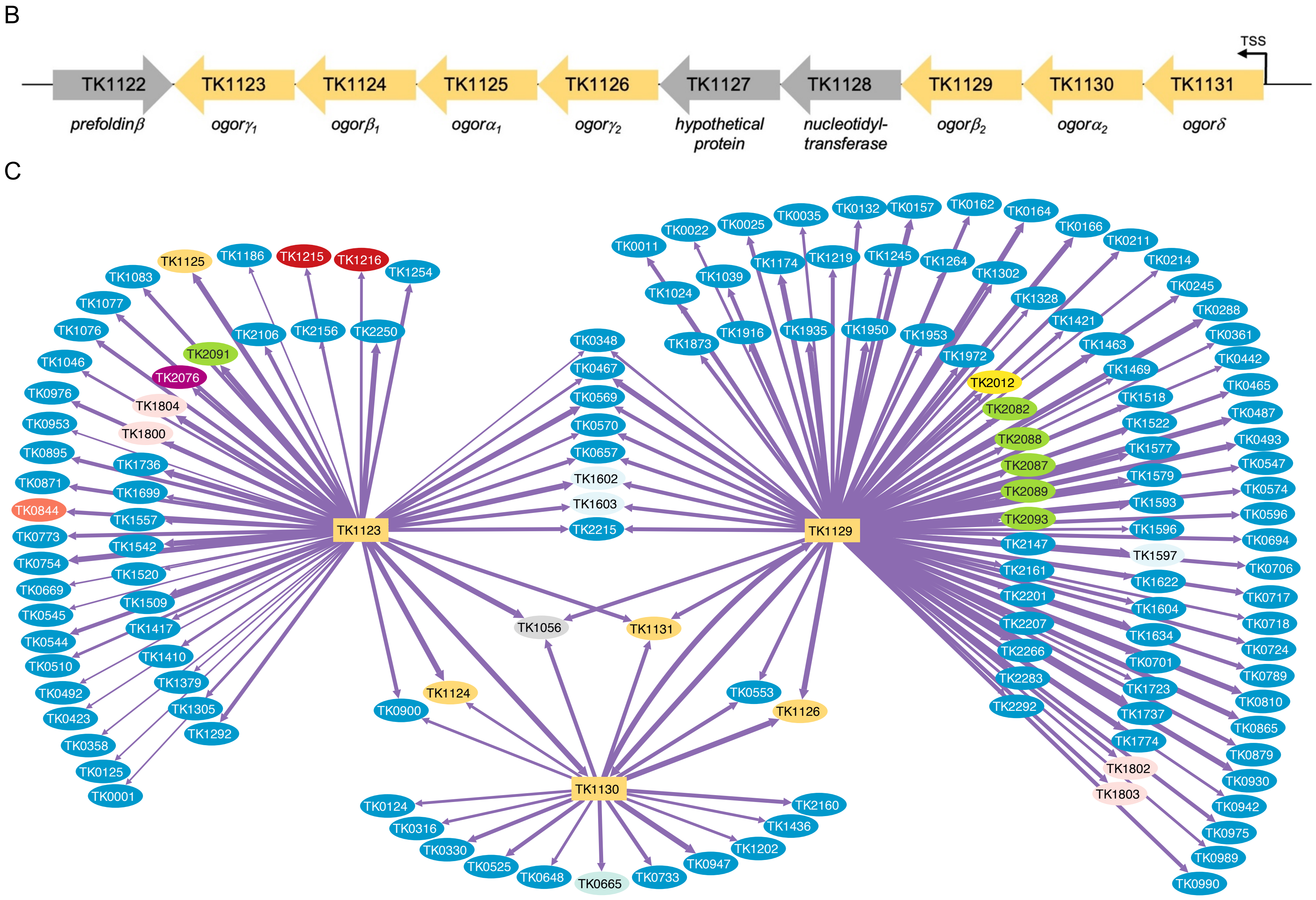
B
TSS
TK1122
TK1123
TK1124
TK1125
TK1126
TK1127
TK1128
TK1129
TK1130
TK1131
prefoldinβ
ogorγ_1
ogorβ_1
ogorα_1
ogorγ_2
hypothetical protein
nucleotidyl-transferase
ogorβ_2
ogorα_2
ogorδ
C
TK1123
TK1129
TK1130
TK1125
TK1186
TK1215
TK1216
TK1254
TK1083
TK1077
TK1076
TK1046
TK0976
TK0953
TK0895
TK0871
TK0844
TK0773
TK0754
TK0669
TK0545
TK0544
TK0510
TK0492
TK0423
TK0358
TK0125
TK0001
TK2106
TK2156
TK2250
TK2091
TK2076
TK1804
TK1800
TK1736
TK1699
TK1557
TK1542
TK1520
TK1509
TK1417
TK1410
TK1379
TK1305
TK1292
TK0348
TK0467
TK0569
TK0570
TK0657
TK1602
TK1603
TK2215
TK1056
TK1131
TK0900
TK1124
TK0553
TK1126
TK0124
TK0316
TK0330
TK0525
TK0648
TK0665
TK0733
TK0947
TK1202
TK1436
TK2160
TK0011
TK0022
TK0025
TK0035
TK0132
TK0157
TK0162
TK0164
TK0166
TK0211
TK0214
TK0245
TK0288
TK0361
TK0442
TK0465
TK0487
TK0493
TK0547
TK0574
TK0596
TK0694
TK0706
TK0717
TK0718
TK0724
TK0789
TK0810
TK0865
TK0879
TK0930
TK0942
TK0975
TK0989
TK0990
TK1024
TK1039
TK1174
TK1219
TK1245
TK1264
TK1302
TK1328
TK1421
TK1463
TK1469
TK1873
TK1916
TK1935
TK1950
TK1953
TK1972
TK2012
TK2082
TK2088
TK2087
TK2089
TK2093
TK2147
TK2161
TK2201
TK2207
TK2266
TK2283
TK2292
TK1518
TK1522
TK1577
TK1579
TK1593
TK1596
TK1597
TK1622
TK1604
TK1634
TK0701
TK1723
TK1737
TK1774
TK1802
TK1803

D

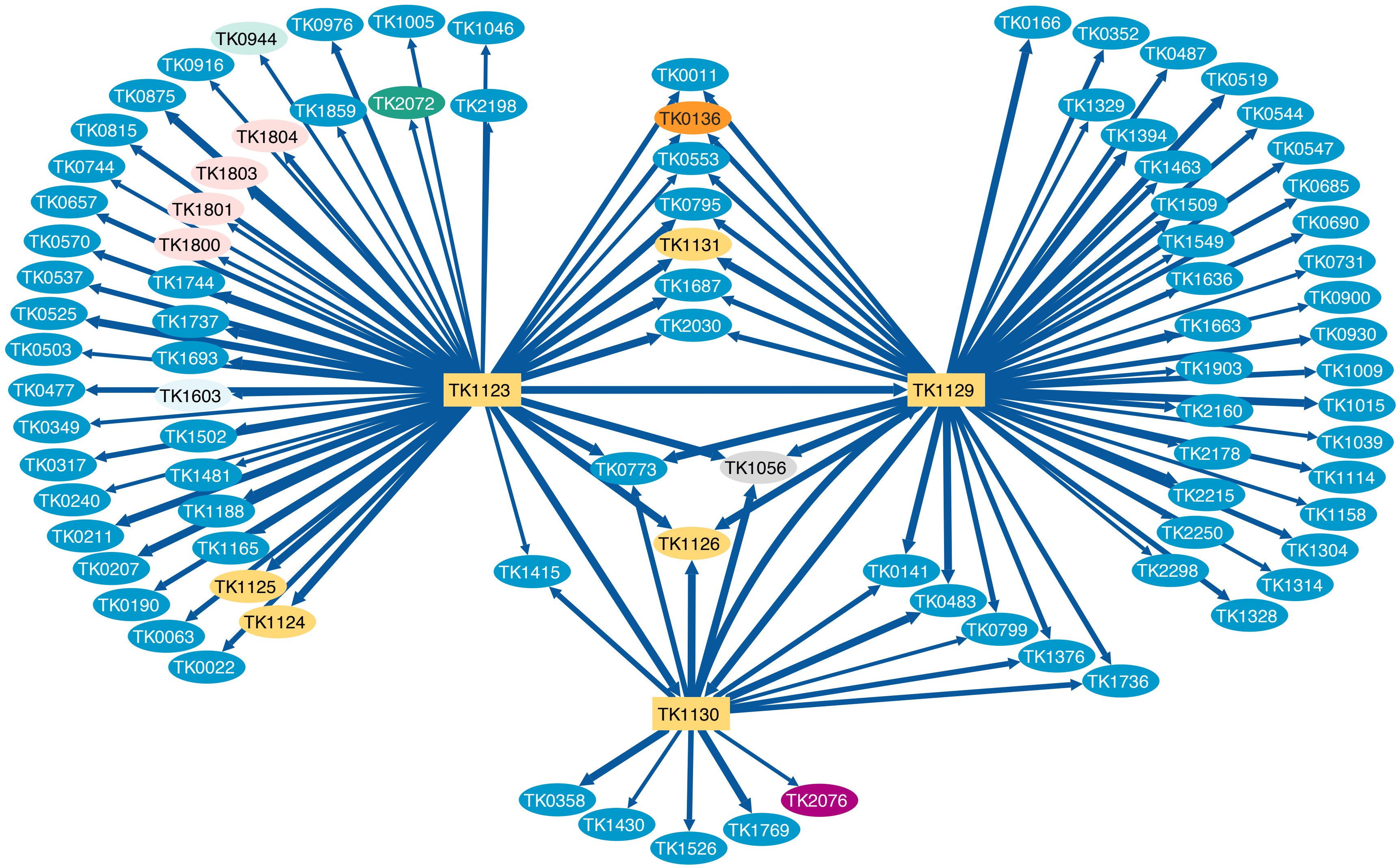

TK1123
TK1129
TK1130
TK0944
TK0976
TK1005
TK1046
TK0916
TK0875
TK0815
TK0744
TK0657
TK0570
TK0537
TK0525
TK0503
TK0477
TK0349
TK0317
TK0240
TK0211
TK0207
TK0190
TK0063
TK0022
TK1804
TK1803
TK1801
TK1800
TK1744
TK1737
TK1693
TK1603
TK1502
TK1481
TK1188
TK1165
TK1125
TK1124
TK1859
TK2072
TK2198
TK0011
TK0136
TK0553
TK0795
TK1131
TK1687
TK2030
TK0773
TK1056
TK1126
TK1415
TK0166
TK0352
TK0487
TK0519
TK0544
TK0547
TK0685
TK0690
TK0731
TK0900
TK0930
TK1009
TK1015
TK1039
TK1114
TK1158
TK1304
TK1314
TK1328
TK1329
TK1394
TK1463
TK1509
TK1549
TK1636
TK1663
TK1903
TK2160
TK2178
TK2215
TK2250
TK2298
TK0141
TK0483
TK0799
TK1376
TK1736
TK0358
TK1430
TK1526
TK1769
TK2076

Figure S6: Metabolic function, gene organization, and co-purifying proteins of the OGOR complex. A) OGOR (gold ellipse) preferentially catabolizes α-ketoglutarate to generate Fd_{red}, acyl-CoA, and CO_2. **B)** The OGOR multimer is encoded by seven genes: TK1131-1129 and TK1126-1123. TK1131 encodes the sole δ subunit, while two α (TK1130 and TK1125), two β (TK1129 and TK1124), and two γ (TK1126 and TK1123) subunits are encoded following TK1131, much like the VOR/POR operon. While two genes disrupt the OGOR operon (TK1127-1128), there is only one transcription start site (TSS). **C)** AP-MS data from three tagged-OGOR subunits (gold rectangles) in +S˚ **(C)** and -S˚ **(D)** conditions identifies co-purifying proteins of the respective tagged-OGOR subunits. All seven OGOR subunits are identified in each network (gold ellipses), though not by all tagged subunits. All tagged OGORs identified RBR3 (TK1056) in both conditions.

A

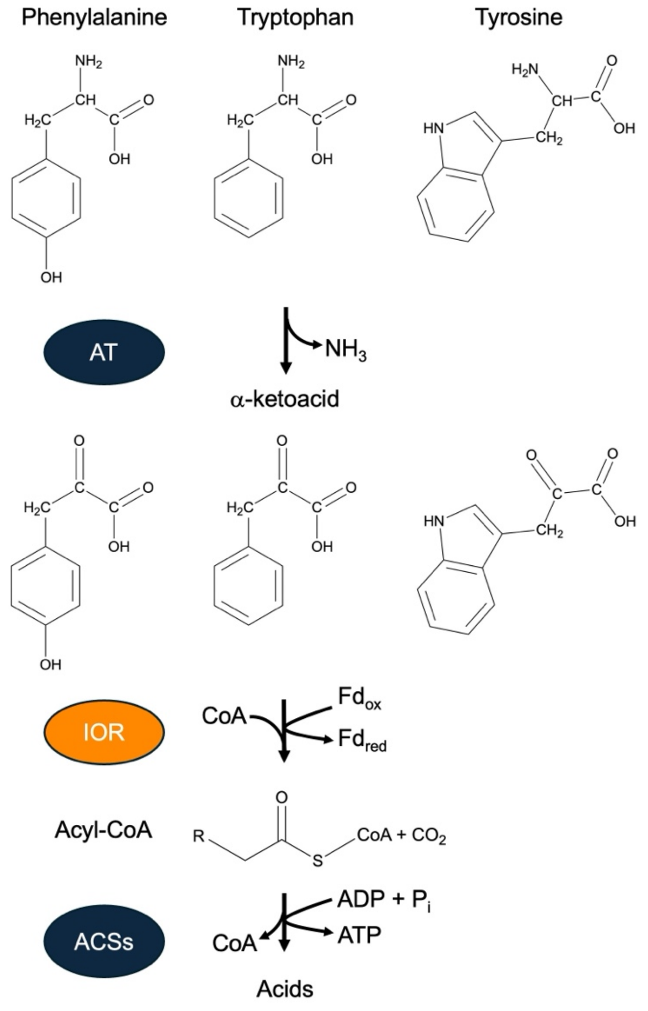
Phenylalanine
Tryptophan
Tyrosine
AT
NH3
α-ketoacid
IOR
CoA
Fdox
Fdred
Acyl-CoA
CoA + CO2
ACSs
ADP + Pi
ATP
CoA
Acids

B

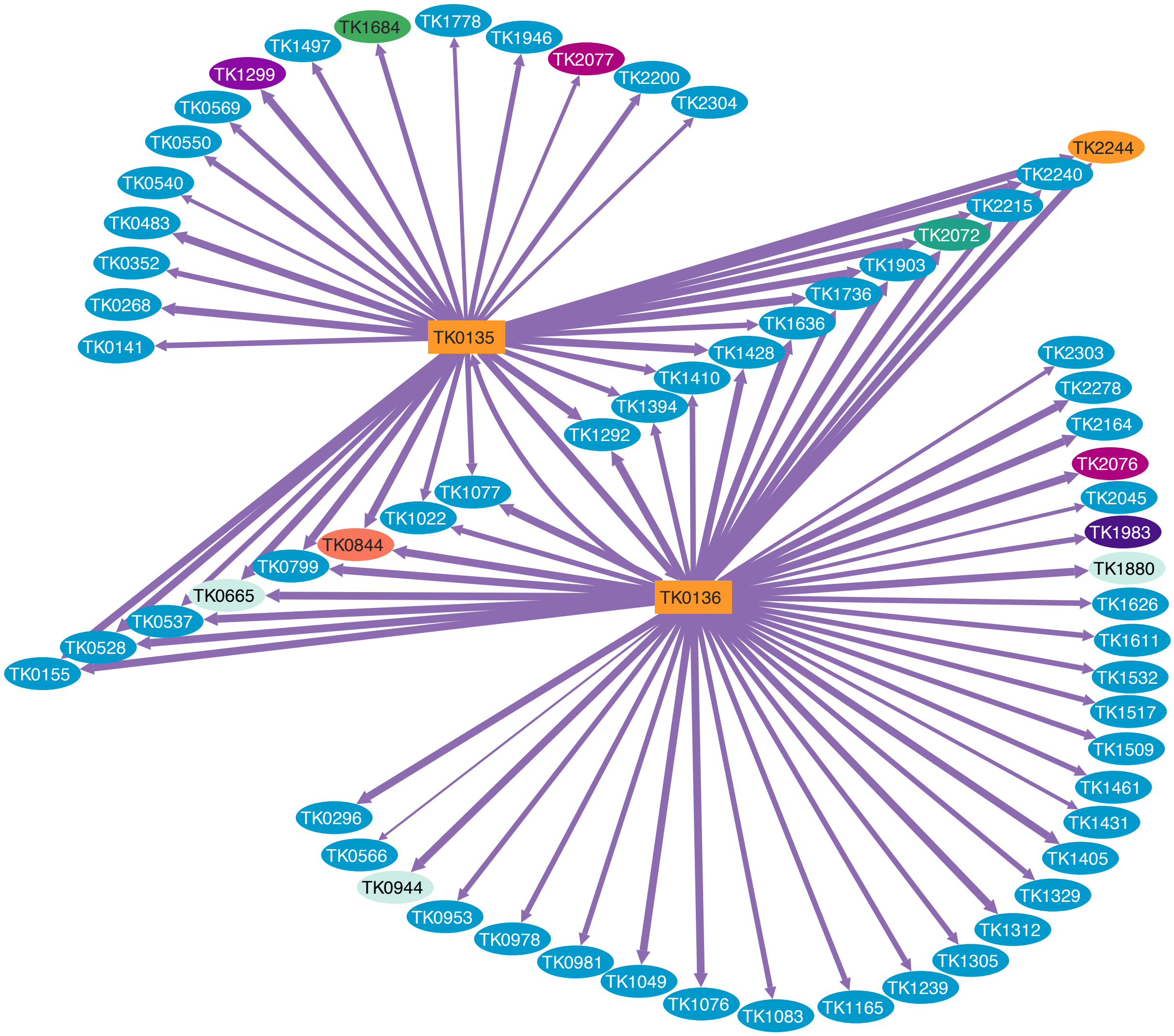

TK0135
TK0136
TK1684
TK1778
TK1946
TK1497
TK2077
TK1299
TK2200
TK2304
TK0569
TK0550
TK2244
TK0540
TK2240
TK2215
TK0483
TK2072
TK0352
TK1903
TK1736
TK0268
TK1636
TK0141
TK1428
TK1410
TK2303
TK1394
TK2278
TK1292
TK2164
TK2076
TK1077
TK2045
TK1022
TK1983
TK0844
TK0799
TK1880
TK0665
TK1626
TK0537
TK1611
TK0528
TK0155
TK1532
TK1517
TK1509
TK1461
TK0296
TK1431
TK0566
TK1405
TK0944
TK1329
TK0953
TK1312
TK0978
TK1305
TK0981
TK1239
TK1049
TK1076
TK1165
TK1083

C

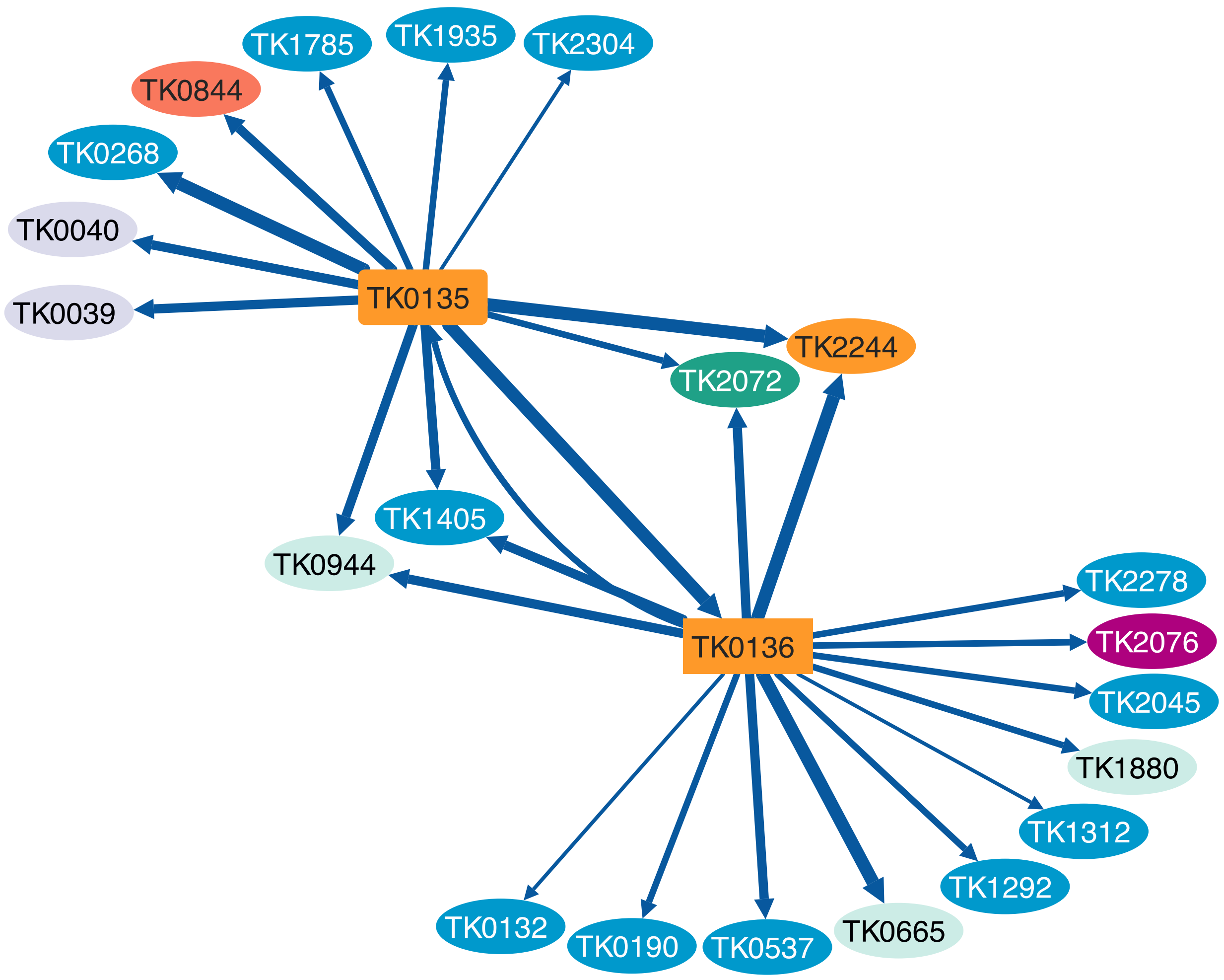

Figure S7: Metabolic function and co-purifying proteins of the IOR complex. A) IOR (orange ellipse) preferentially catabolizes α-ketoacids of deaminated aromatic amino acids to produce Fd_{red}, acyl-CoA, and CO_2. **B)** AP-MS data for tagged-IORα (TK0136) and IORβ (TK0135) (orange rectangles) identified > ~2X proteins in +S˚ **(B)** compared to -S˚ **(C)** conditions. Both tagged-IOR subunits identified one another in both conditions.

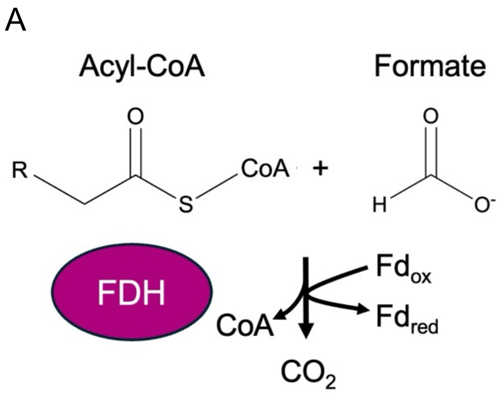
A
Acyl-CoA
Formate
O
R
S
CoA
+
O
H
O-
FDH
Fdox
Fdred
CoA
CO2

B

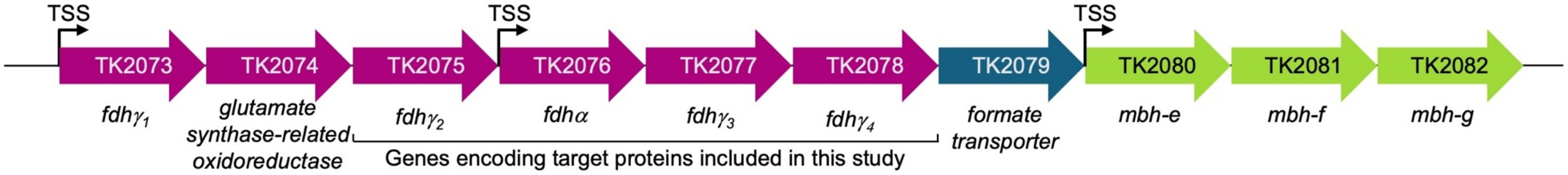

TSS
TSS
TSS
TK2073
TK2074
TK2075
TK2076
TK2077
TK2078
TK2079
TK2080
TK2081
TK2082
fdhγ1
glutamate synthase-related oxidoreductase
fdhγ2
fdhα
fdhγ3
fdhγ4
formate transporter
mbh-e
mbh-f
mbh-g
Genes encoding target proteins included in this study

C

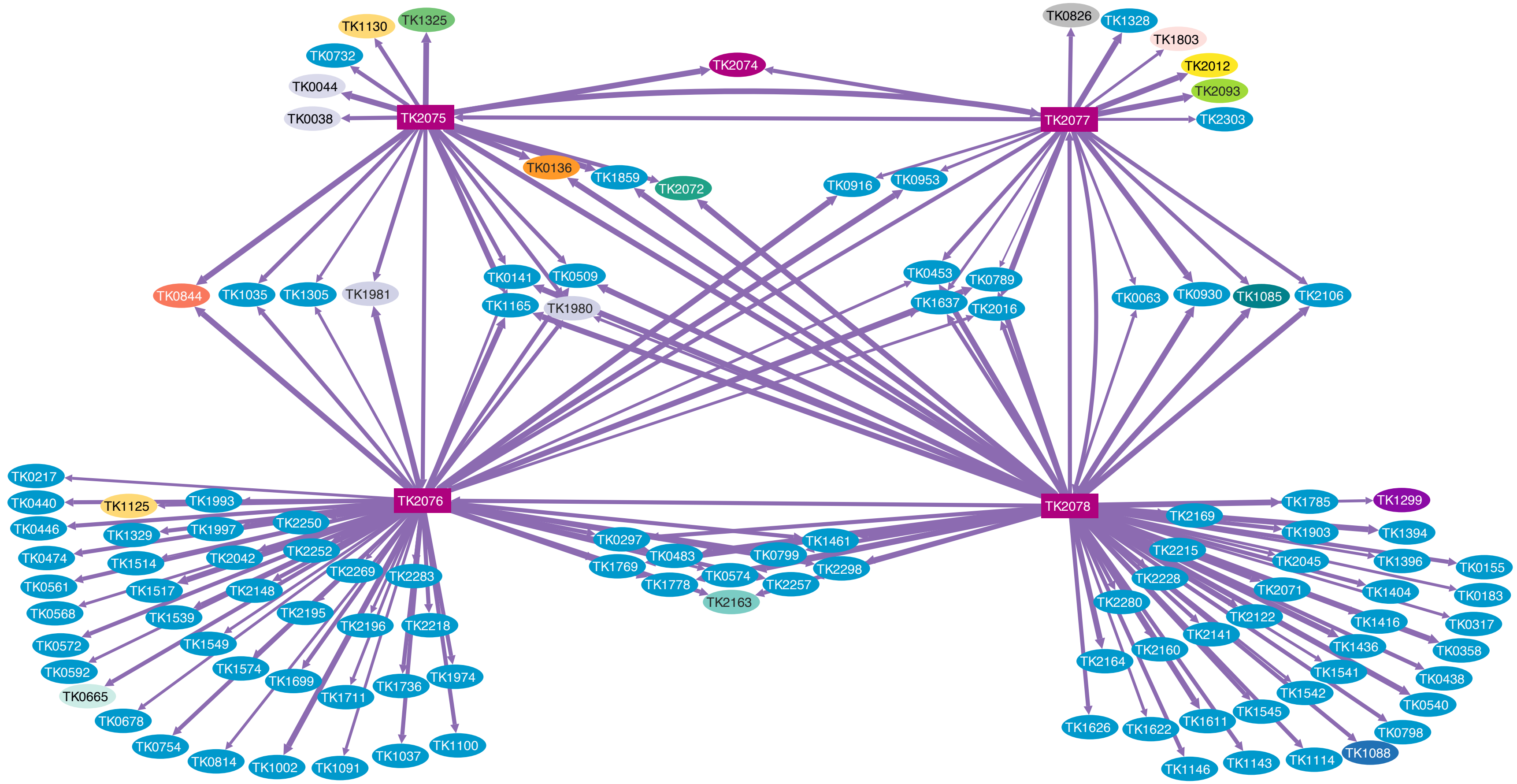

TK1130
TK1325
TK0732
TK0044
TK0038
TK2075
TK2074
TK0826
TK1328
TK1803
TK2012
TK2093
TK2303
TK2077
TK0136
TK1859
TK2072
TK0916
TK0953
TK0844
TK1035
TK1305
TK1981
TK0141
TK0509
TK1165
TK1980
TK0453
TK0789
TK1637
TK2016
TK0063
TK0930
TK1085
TK2106
TK0217
TK0440
TK1125
TK1993
TK0446
TK1329
TK1997
TK2250
TK0474
TK1514
TK2042
TK2252
TK0561
TK1517
TK2269
TK2283
TK0568
TK2148
TK1539
TK2195
TK0572
TK1549
TK2196
TK2218
TK0592
TK1574
TK0665
TK1699
TK1736
TK1974
TK0678
TK1711
TK0754
TK0814
TK1002
TK1091
TK1037
TK1100
TK2076
TK0297
TK1769
TK0483
TK1778
TK0574
TK2163
TK0799
TK1461
TK2298
TK2257
TK2078
TK1785
TK1299
TK2169
TK1903
TK1394
TK2215
TK2045
TK1396
TK0155
TK2228
TK2071
TK1404
TK0183
TK2280
TK2122
TK1416
TK0317
TK2141
TK1436
TK0358
TK2164
TK2160
TK1541
TK0438
TK1542
TK0540
TK1545
TK1626
TK1622
TK1611
TK0798
TK1146
TK1143
TK1114
TK1088

D

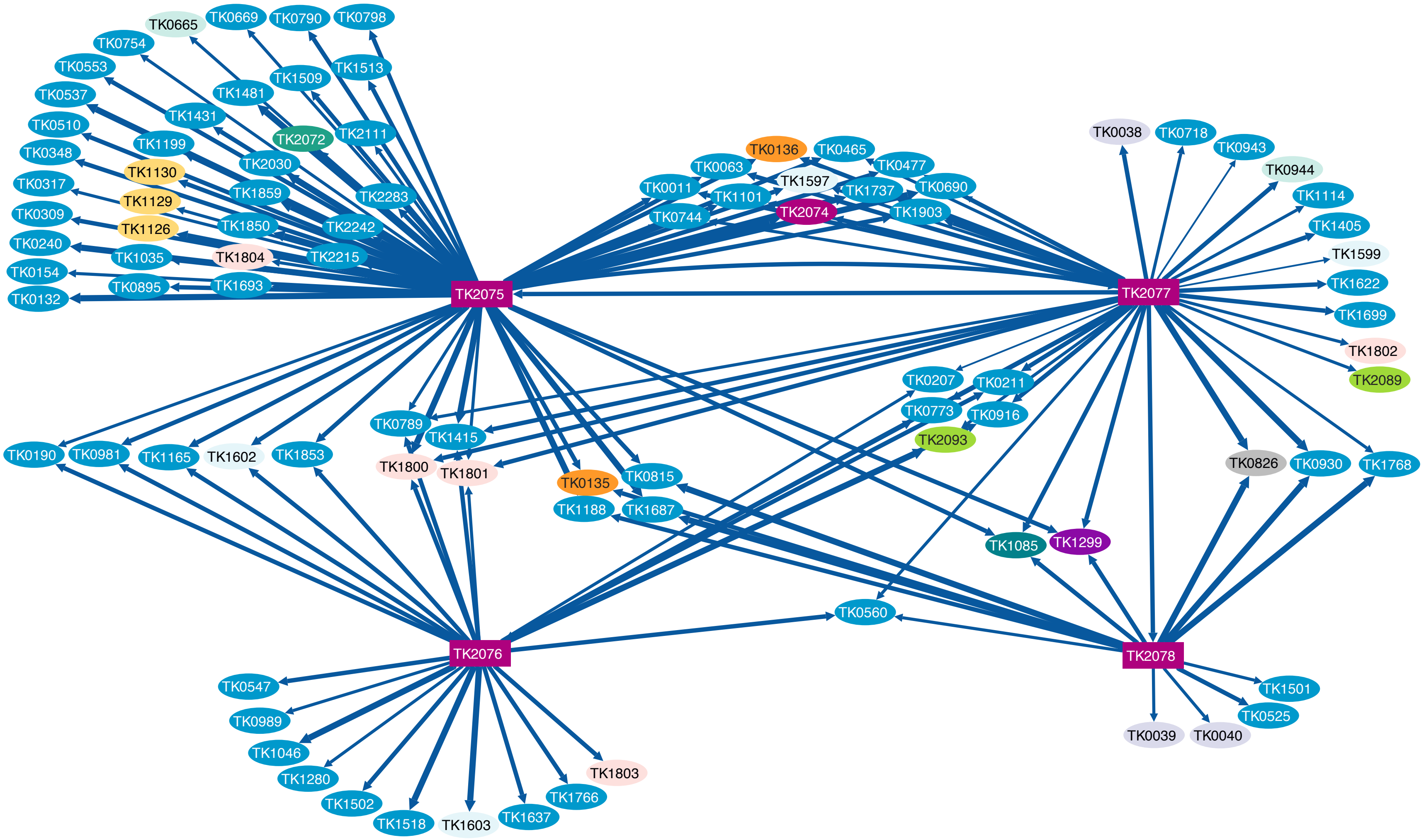

Figure S8: Metabolic function, gene organization, and co-purifying proteins of FDH-related proteins. A) FDH catalyzes the conversion of acyl-CoA and formate to generate Fd_{red} and CO_2. **B)** FDH-related genes (fuschia, TK2073 -

2078) are encoded in a gene cluster downstream of the MBH operon (TK2080 - 2093). TK2075 -TK2078 encode FDHγ_2, FDHα, FDHγ_3, and FDHγ_4, respectively, and were targets for AP-MS. Significant co-purifying proteins in +S˚ **(C)** and -S˚ **(D)** are shown in colored ellipses. No proteins were identified by all four tagged FDH-related proteins in +S˚ or -S˚. TK2077 and TK2078 identified one another in +S˚, as did TK2077 and TK2075. TK2075 identified TK2078 and TK2076, but these associations were not observed reciprocally. Both TK2077 and TK2075 were found to associate with TK2074, annotated as a glutamate synthase related oxidoreductase but homologous to *To*-FDH3 and encoded within the FDH-operon. While TK2073 (FDHγ_1) is reported as a 4Fe-4S cluster binding protein related to FDH, it was not identified as significant with any tagged FDH-related protein. TK2075 and TK2077 were seen to associate with more proteins in -S˚ than in +S˚, while TK2076 and TK2078 showed the opposite. TK2076 and TK2078 identified eighteen similar interacting partners in +S˚ but only one in -S˚.

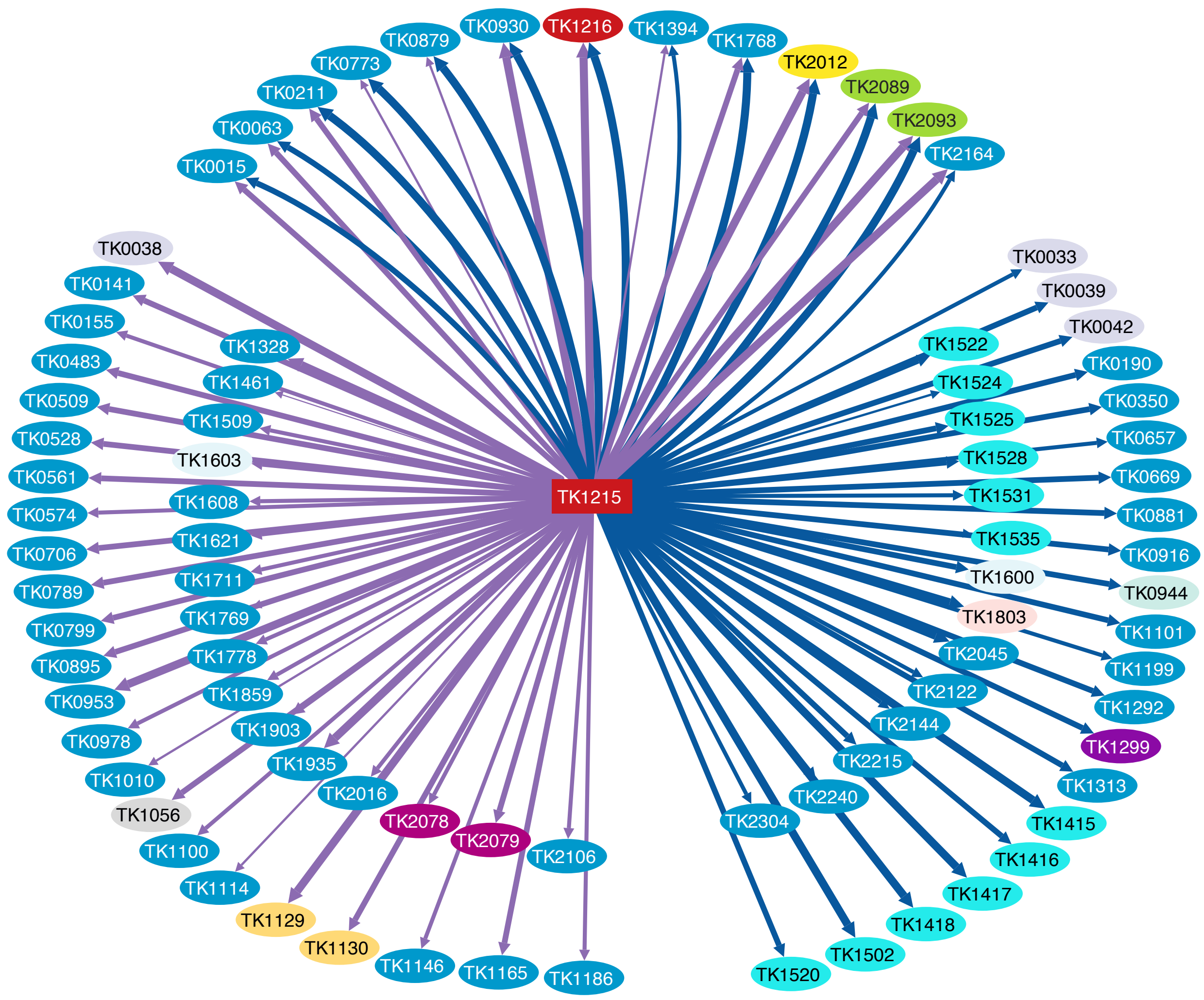

Figure S9: Co-purifying proteins of MBS-L. AP-MS data for the tagged subunit of MBS-L (TK1215, red rectangle) identified ~ fifty interacting partners in both +S$^\circ$ (purple lines, at left) and -S$^\circ$ (blue lines, at right) conditions, with thirteen of these interacting partners being identified in both conditions (purple and blue lines, at top). One other subunit of MBS, MBS-K (TK1216, red ellipse) was identified in both conditions, along with two subunits of MBH (TK2089 and TK2093, lime green ellipses), and Fd3 (TK2012, yellow ellipse).

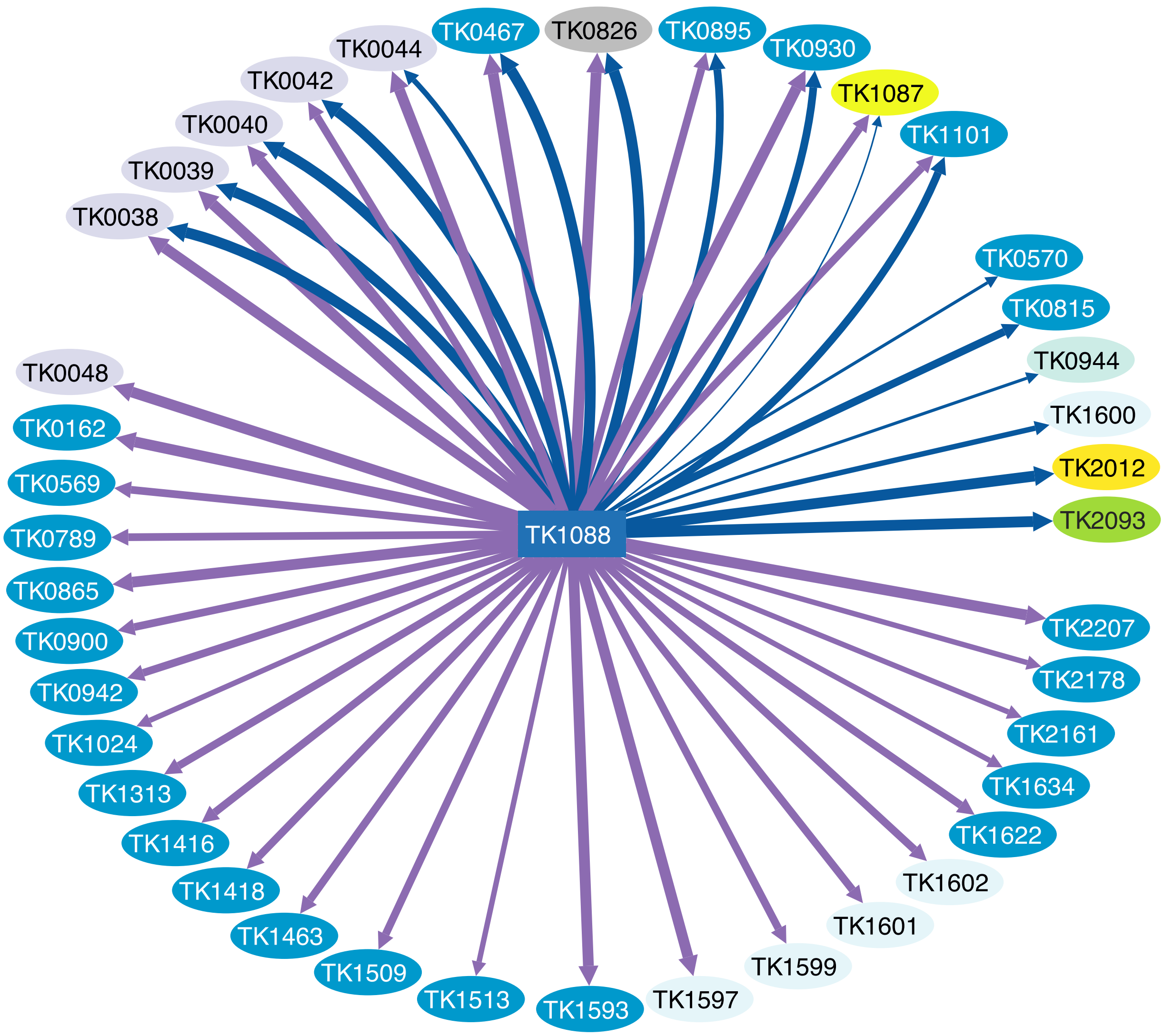

Figure S10: Co-purifying proteins of GGR. AP-MS data of tagged GGR (TK1088, dark blue rectangle) identified a greater number of interacting partners in +S˚ (purple lines) than in -S˚ (blue lines), however eleven partners were identified in both conditions including Fd2 (TK1087, lime green ellipse), RBR2 (TK0826, grey ellipse) and five subunits of the flagellin complex (TK0038 - TK0044, light grey ellipses). Another Fd, Fd3 (TK2012, yellow ellipse) and the MBH-N subunit (TK2093, green ellipse) of the MBH complex were identified -S˚ conditions.

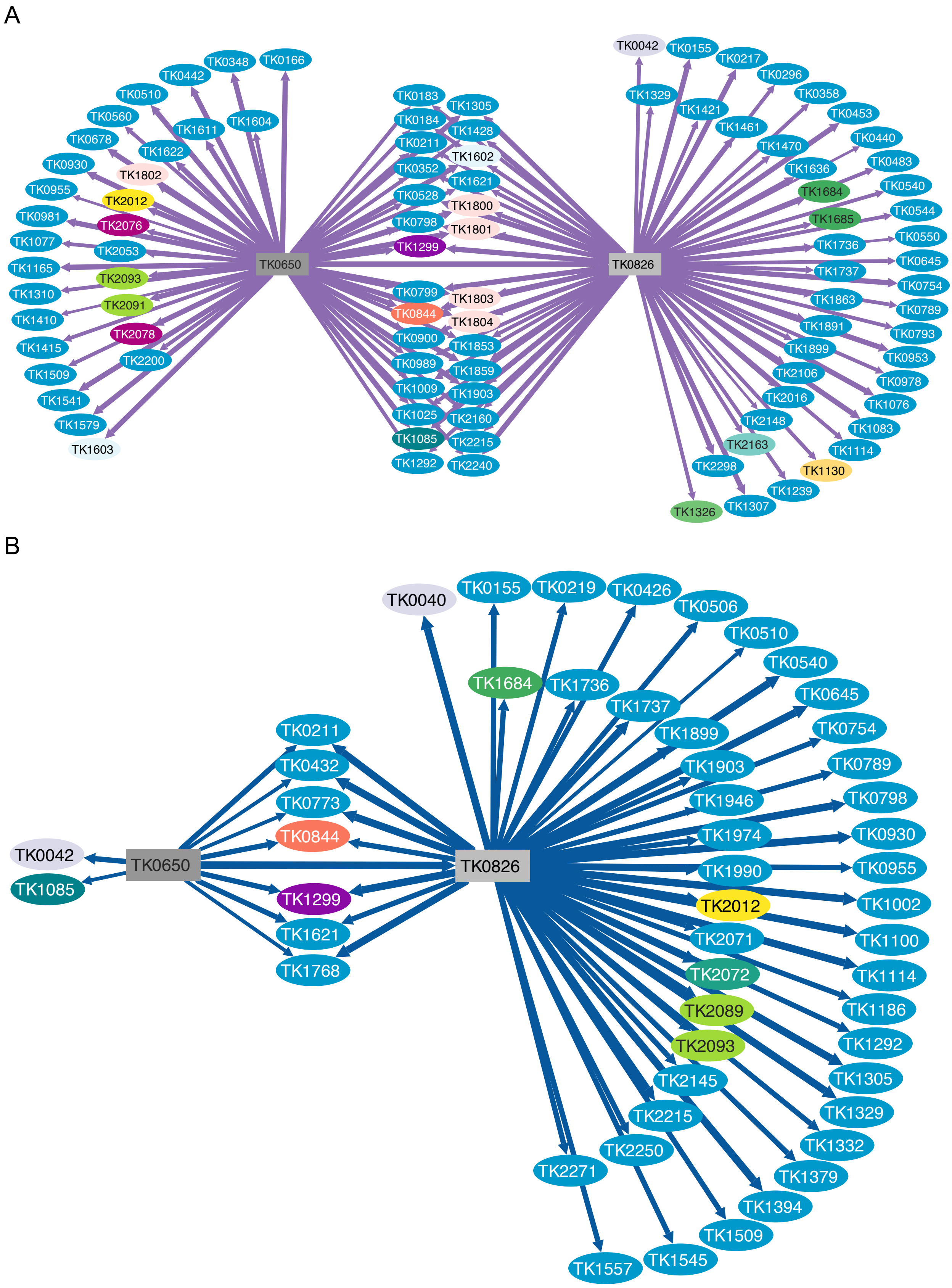
A
TK0650
TK0826
TK0166
TK0348
TK0442
TK0510
TK0560
TK0678
TK0930
TK0955
TK0981
TK1077
TK1165
TK1310
TK1410
TK1415
TK1509
TK1541
TK1579
TK1603
TK1604
TK1611
TK1622
TK1802
TK2012
TK2076
TK2053
TK2093
TK2091
TK2078
TK2200
TK0183
TK0184
TK0211
TK0352
TK0528
TK0798
TK1299
TK0799
TK0844
TK0900
TK0989
TK1009
TK1025
TK1085
TK1292
TK1305
TK1428
TK1602
TK1621
TK1800
TK1801
TK1803
TK1804
TK1853
TK1859
TK1903
TK2160
TK2215
TK2240
TK0042
TK0155
TK0217
TK0296
TK0358
TK0453
TK0440
TK0483
TK0540
TK0544
TK0550
TK0645
TK0754
TK0789
TK0793
TK0953
TK0978
TK1076
TK1083
TK1114
TK1130
TK1239
TK1307
TK1326
TK1329
TK1421
TK1461
TK1470
TK1636
TK1684
TK1685
TK1736
TK1737
TK1863
TK1891
TK1899
TK2106
TK2016
TK2148
TK2163
TK2298
B
TK0042
TK1085
TK0650
TK0211
TK0432
TK0773
TK0844
TK1299
TK1621
TK1768
TK0826
TK0040
TK0155
TK0219
TK0426
TK0506
TK0510
TK0540
TK0645
TK0754
TK0789
TK0798
TK0930
TK0955
TK1002
TK1100
TK1114
TK1186
TK1292
TK1305
TK1329
TK1332
TK1379
TK1394
TK1509
TK1545
TK1557
TK1684
TK1736
TK1737
TK1899
TK1903
TK1946
TK1974
TK1990
TK2012
TK2071
TK2072
TK2089
TK2093
TK2145
TK2215
TK2250
TK2271

Figure S11: Co-purifying proteins of two rubrerythrins. Two rubrerythrins (grey rectangles) were tagged for AP-MS analysis: RBR1 (TK0650) and RBR2 (TK0826). Resulting proteins identified as interacting partners are shown here in +S˚ **(A)** and -S˚ conditions **(B)**.

A

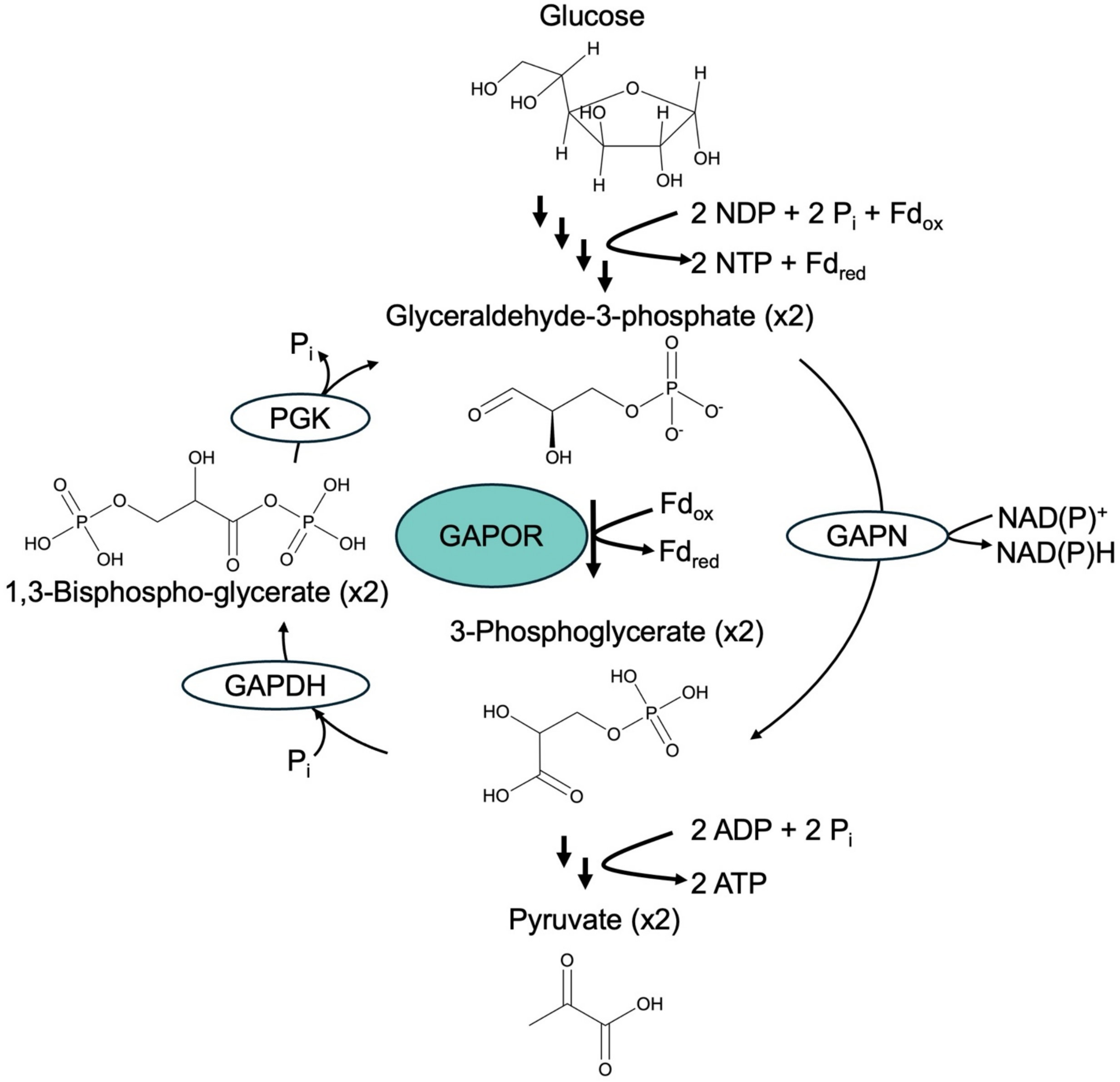

Glucose
2 NDP + 2 P_i + Fd_{ox}
2 NTP + Fd_{red}
Glyceraldehyde-3-phosphate (x2)
P_i
PGK
1,3-Bisphospho-glycerate (x2)
GAPOR
Fd_{ox}
Fd_{red}
GAPN
$NAD(P)^+$
NAD(P)H
3-Phosphoglycerate (x2)
GAPDH
P_i
2 ADP + 2 P_i
2 ATP
Pyruvate (x2)

B

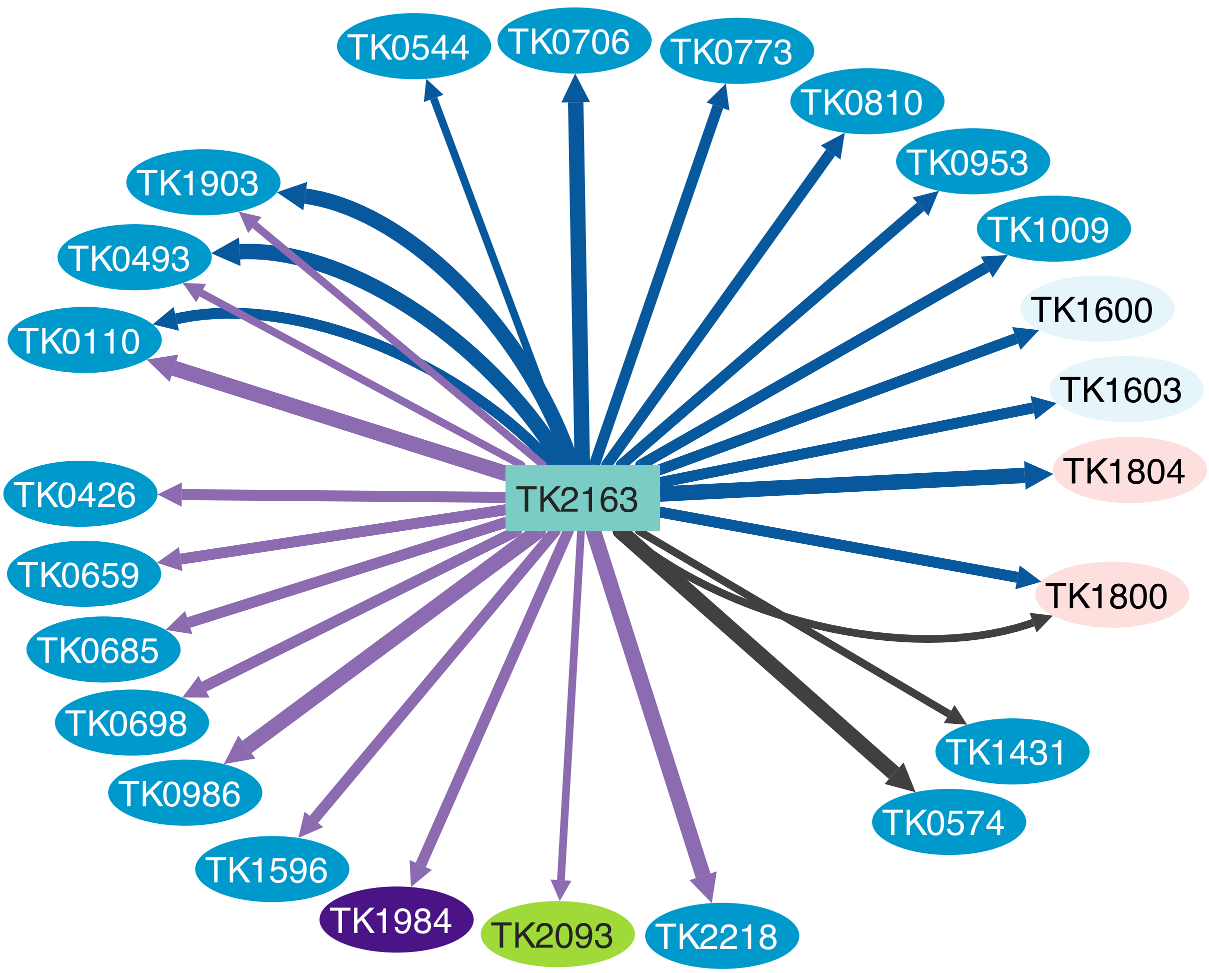

Figure S12: Co-purifying proteins of glyceraldehyde-3-phosphate oxidoreductase (GAPOR). A) *T. kodakarensis* maintains a modified Embden-Meyerhof glycolytic pathway which utilizes either glyceraldehyde-3-phosphate (GAP) oxidoreductase (GAPOR, TK2163) or GAP dehydrogenase (non-phosphorylating, GAPN, TK0705) to convert GAP into 3-phosphoglycerate, generating Fd_{red} or NAD(P)H, respectively. 3-phosphoglycerate is converted by GAPDH (phosphorylating, GAPDH, TK0765) into 1,3-bisphosphoglycerate which is converted by phosphoglycerate kinase (PGK, TK1146) to GAP in gluconeogenic conditions. **B)** As GAPOR (light blue rectangle) is primarily active in glycolysis, pyruvate-limited conditions (black connecting lines at lower right) were included in addition to +S˚ (purple lines) and -S˚ (blue lines) conditions.

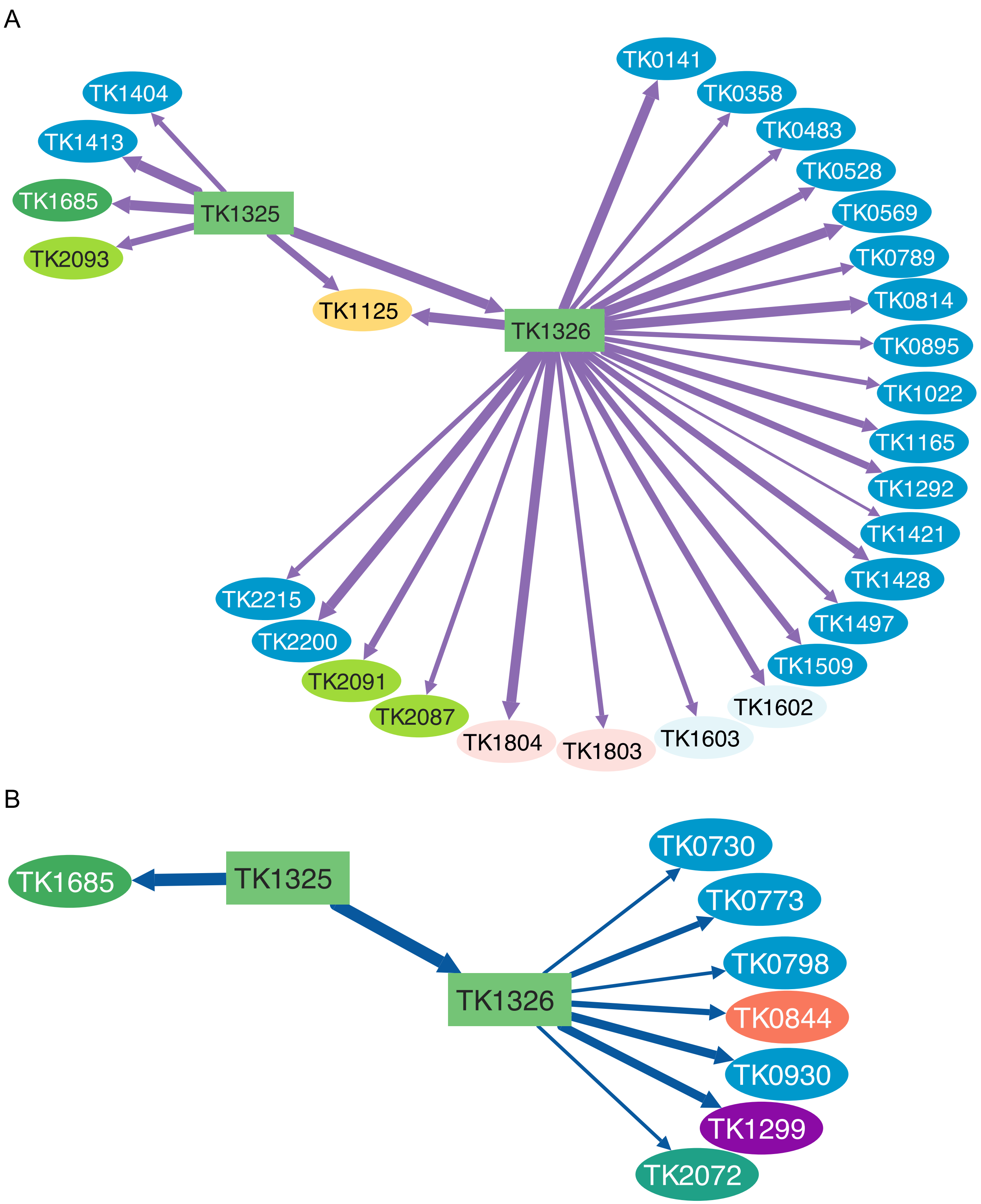

Figure S13: Co-purifying proteins of ferredoxin NAD(P)H oxidoreductase 1 (FNOR1). FNOR1 is composed of an α (TK1325) and a β subunit (TK1326) (light green rectangles). AP-MS data shows interacting partners for FNOR1 in +S° **(A)** and -S° **(B)**

conditions. Slightly more interactions were observed in +S° conditions, supporting previous evidence that FNOR1 is more active in the presence of sulfur. FNOR1α identified FNOR1β, as well as FNOR2α (TK1685) in both conditions.

A

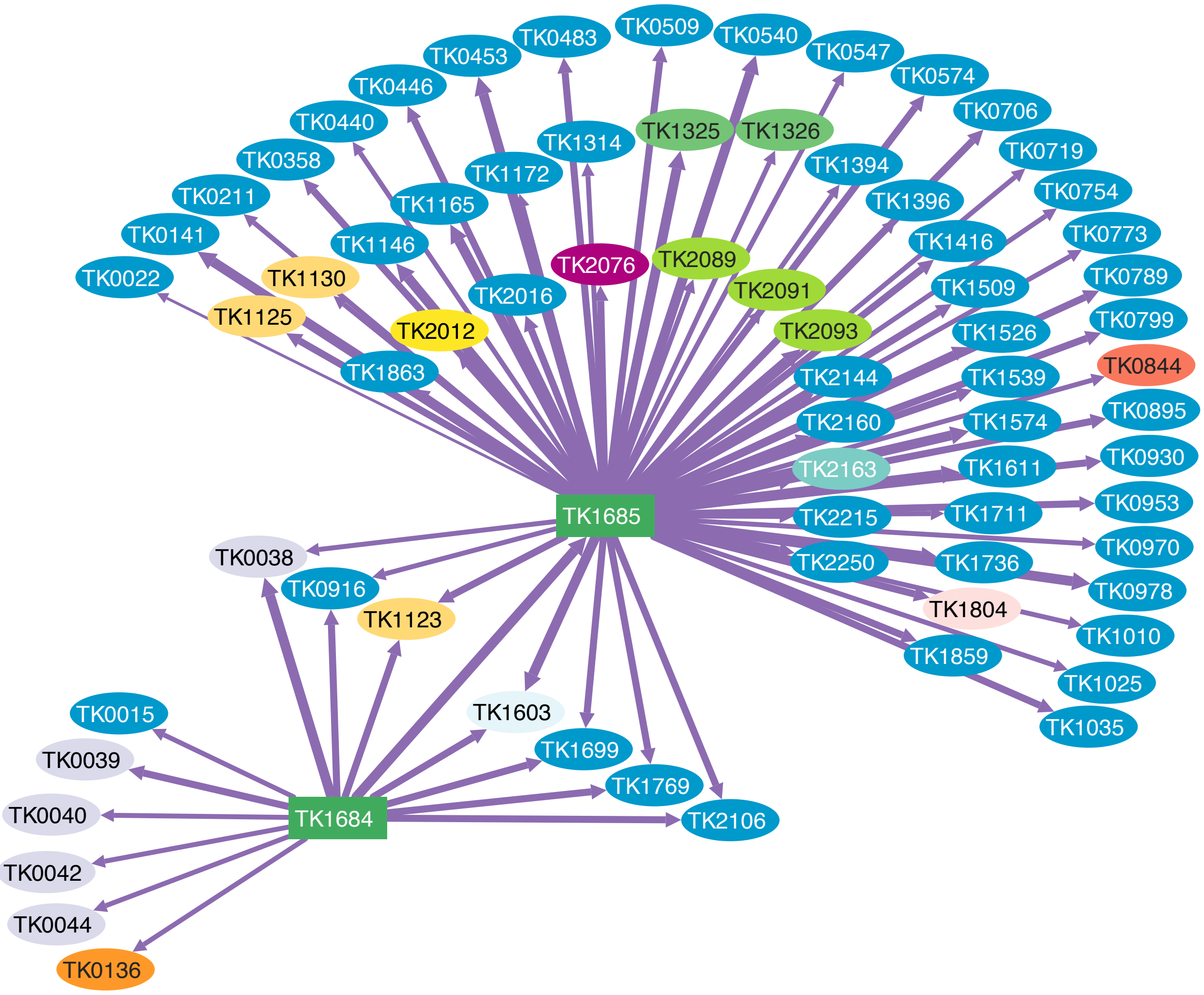

TK1685
TK1684
TK0022
TK0141
TK0211
TK0358
TK0440
TK0446
TK0453
TK0483
TK0509
TK0540
TK0547
TK0574
TK0706
TK0719
TK0754
TK0773
TK0789
TK0799
TK0844
TK0895
TK0930
TK0953
TK0970
TK0978
TK1010
TK1025
TK1035
TK1130
TK1125
TK1146
TK1165
TK1172
TK1314
TK1325
TK1326
TK1394
TK1396
TK1416
TK1509
TK1526
TK1539
TK1574
TK1611
TK1711
TK1736
TK1804
TK1859
TK2012
TK2016
TK2076
TK2089
TK2091
TK2093
TK2144
TK2160
TK2163
TK2215
TK2250
TK1863
TK0038
TK0916
TK1123
TK1603
TK1699
TK1769
TK2106
TK0015
TK0039
TK0040
TK0042
TK0044
TK0136

B

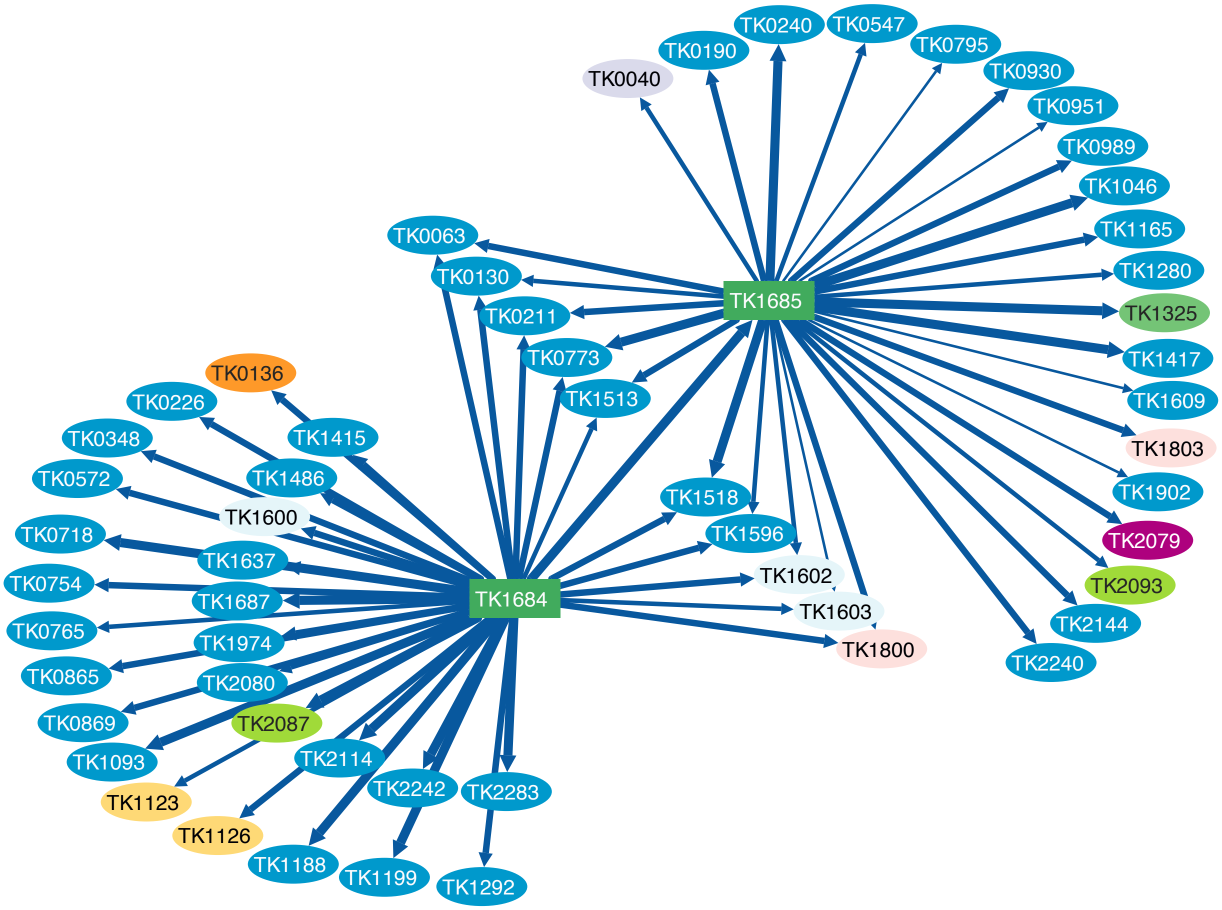

Figure S14: Co-purifying proteins of ferredoxin NAD(P)H oxidoreductase 2 (FNOR2). FNOR2 is composed of an α (TK1684) and a β subunit (TK1685) (green rectangles) and both proteins were tagged and analyzed in +S˚ **(A)** and -S˚ **(B)** conditions.

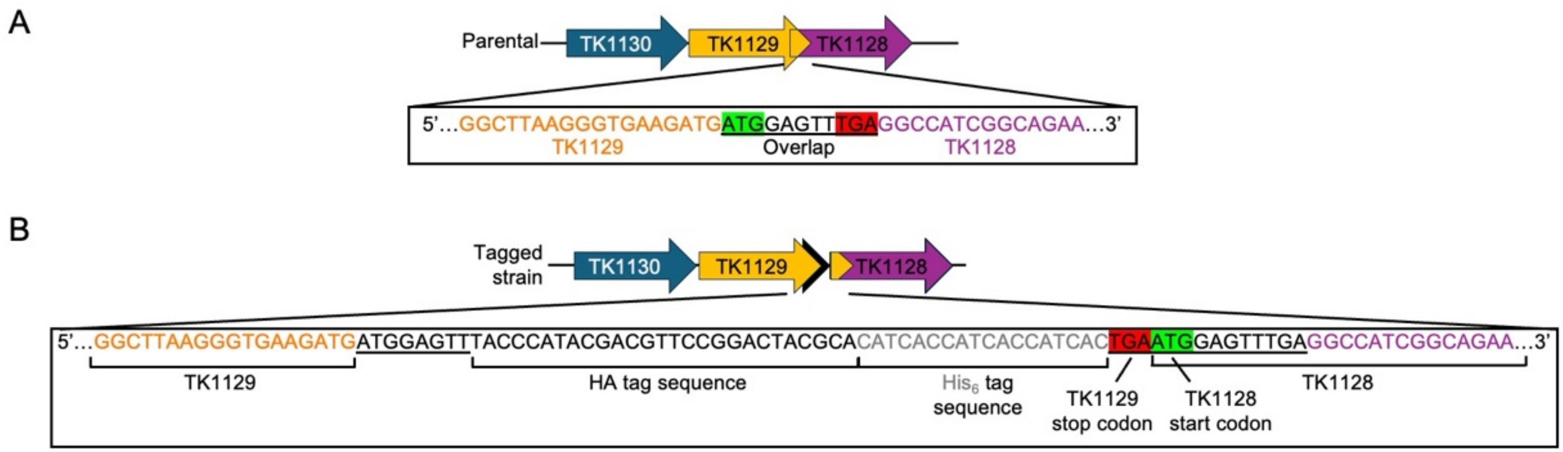

Figure S15: Overlapping genomic regions are accounted for when generating tagged strains. A) The *T. kodakarensis* genome is compact and contains many overlapping genetic elements. **B)** In the case that two gene sequences overlap (where the 3' end of the gene of interest occurs within the coding region of the downstream gene) the tag sequence is inserted directly upstream of the stop codon of the target gene and the overlapping genetic element is repeated following the insertion such that both genes produce whole protein products.
