# Supplementary material for: A dynamic protein interactome drives energy conservation and electron flux in Thermococcus kodakarensis
Source: Appl Environ Microbiol. 2025 Apr 3;91(4):e00293-25. doi: 10.1128/aem.00293-25 (PMC12016516; doi:10.1128/aem.00293-25)
Supplement: Supplemental tables — Tables S1 to S25. [file aem.00293-25-s0004.pdf]

SUPPLEMENTAL TABLES

A dynamic protein interactome drives energy conservation and electron flux in *Thermococcus kodakarensis*

Table S1: Co-purified protein partners of VORγ/PORγ (TK1978) in sulfur and non-sulfur conditions.

Co-purifying proteins	Gene No.	+S° p-value	+S° Fold Change	+S° Rank	-S° p-value	-S° Fold Change	-S° Rank
Rubrerythrin-related protein	TK0650	0.00001	∞	5	0.00001	∞	5
Rubrerythrin domain-containing protein	TK0826	0.00001	∞	5	0.00001	∞	5
Uncharacterized protein	TK1958	0.0044	∞	5	0.018	∞	4
Pyruvate/ketoisovalerate oxidoreductases common subunit gamma	TK1978	0.00001	49	5	0.00001	62	5
2-oxoisovalerate:ferredoxin oxidoreductase, delta subunit	TK1979	0.00001	∞	5	0.00001	200	5
2-oxoisovalerate:ferredoxin oxidoreductase, alpha subunit	TK1980	0.00001	83	5	0.00001	270	5
2-oxoisovalerate:ferredoxin oxidoreductase, beta subunit	TK1981	0.00001	160	5	0.00001	∞	5
Pyruvate:ferredoxin oxidoreductase, delta subunit	TK1982	0.00001	∞	5	0.00001	140	5
Pyruvate:ferredoxin oxidoreductase, alpha subunit	TK1983	0.00001	93	5	0.00001	60	5
Pyruvate:ferredoxin oxidoreductase, beta subunit	TK1984	0.00001	420	5	0.00001	340	5
Putative snRNP Sm-like protein	TK0976	0.00001	1.9	4			
Pyrrolidone-carboxylate peptidase	TK1835	0.038	∞	4			
Probable formate dehydrogenase, alpha subunit	TK2076	0.00001	1.7	4			
Orotidine 5'-phosphate decarboxylase	TK2276	0.02	∞	4			

Table S2: Co-purified protein partners of VORδ (TK1979) in sulfur and non-sulfur conditions.

Co-purifying proteins	Gene No.	+S° p-value	+S° Fold Change	+S° Rank	-S° p-value	-S° Fold Change	-S° Rank
UPF0173 metal-dependent hydrolase TK0141	TK0141	0.026	7.6	4	0.032	6.8	4
RecJ-like exonuclease, containing OB-fold nucleic acid-binding domains	TK0155	0.0034	∞	5	0.013	18	4
Uncharacterized protein	TK0483	0.049	6.2	4	0.00001	18	5
FeS_assembly_P domain-containing protein	TK0527	0.0049	∞	5	0.00041	∞	5
Serine hydroxymethyltransferase	TK0528	0.0024	∞	5	0.028	7	4
Rubrerythrin-related protein	TK0650	0.00001	∞	5	0.00001	∞	5
Predicted AP endonuclease	TK1165	0.00029	8.2	5	0.03	6.3	4
Probable lipoprotein releasing system, ATP-binding protein	TK1859	0.035	12	4	0.004	22	5
DNA polymerase II large subunit	TK1903	0.00043	11	5	0.008	5.5	5
Uncharacterized protein	TK1958	0.0085	∞	5	0.0081	∞	5
Pyruvate/ketoisovalerate oxidoreductases common subunit gamma	TK1978	0.00001	24	5	0.00001	32	5
2-oxoisovalerate:ferredoxin oxidoreductase, delta subunit	TK1979	0.00001	150	5	0.00001	88	5
2-oxoisovalerate:ferredoxin oxidoreductase, alpha subunit	TK1980	0.00001	41	5	0.00001	48	5
2-oxoisovalerate:ferredoxin oxidoreductase, beta subunit	TK1981	0.00001	90	5	0.00001	64	5

Pyruvate:ferredoxin oxidoreductase, beta subunit	TK1984	0.003	18	5	0.03	6.3	4
Amidophosphoribosyltransferase	TK0211	0.03	12	4			
Proline--tRNA ligase	TK0550	0.039	18	4			
tRNA(Met) cytidine acetyltransferase TmcA	TK0754	0.018	21	4			
Type 2 DNA topoisomerase 6 subunit B	TK0799	0.00001	12	5			
L-threonine 3-dehydrogenase	TK0916	0.046	11	4			
Adenylosuccinate synthetase	TK1002	0.021	∞	4			
Probable translation initiation factor IF-2	TK1305	0.018	8.5	4			
Nucleoside diphosphate kinase	TK1307	0.046	11	4			
Predicted ATPase, AAA superfamily	TK1314	0.034	19	4			
Probable tRNA pseudouridine synthase B	TK1509	0.015	9.7	4			
Predicted metal-dependent hydrolase	TK1611	0.037	∞	4			
Ferredoxin:NADP oxidoreductase, alpha subunit	TK1684	0.0085	∞	5			
Ferredoxin:NADP oxidoreductase, beta subunit	TK1685	0.031	∞	4			
Vitamin B12-dependent ribonucleotide reductase	TK1736	0.0048	5.1	5			
ABC-type dipeptide/oligopeptide transport system, probable periplasmic component	TK1804	0.02	11	4			
Fibrillarin-like rRNA/tRNA 2'-O-methyltransferase	TK0183				0.035	15	4
GMP synthase [glutamine-hydrolyzing] subunit A	TK0190				0.02	∞	4
Sugar-phosphate nucleotidyltransferase	TK0219				0.013	18	4
Metal-dependent phosphohydrolase, HD superfamily	TK0540				0.019	11	4
Predicted ATP-dependent endonuclease, OLD family	TK0773				0.02	∞	4
N2, N2-dimethylguanosine tRNA methyltransferase	TK0981				0.044	5	4
Putative 5-methylcytosine restriction system, GTPase subunit	TK1009				0.032	6.8	4
30S ribosomal protein S13	TK1506				0.0081	∞	5
Adenylate kinase	TK1517				0.043	14	4
SAM-dependent methyltransferase, UPF0020 family	TK2045				0.00013	∞	4
Non-specific serine/threonine protein kinase	TK2250				0.014	5.6	4

Table S3: Co-purified protein partners of VORα (TK1980) in sulfur and non-sulfur conditions.

		+S°			-S°		
**Co-purifying proteins**	**Gene No.**	**p-value**	**Fold Change**	**Rank**	**p-value**	**Fold Change**	**Rank**
Flagellin B1	TK0038	0.00001	4.5	5	0.00001	31	5
Flagellin B2	TK0039	0.00001	3.8	4	0.00001	40	5
Flagellin B3	TK0040	0.00001	8.2	5	0.00001	∞	5
Flagellin B5	TK0042	0.00001	4.1	5	0.00013	23	5
DUF3216 domain-containing protein	TK0318	0.017	2.7	3	0.0011	∞	4

Acetate--CoA ligase (ADP-forming)	TK0665	0.004	2.2	4	0.00051	9	5
Uncharacterized protein	TK1394	0.00001	2.6	4	0.012	∞	4
Pyruvate/ketoisovalerate oxidoreductases common subunit gamma	TK1978	0.00061	2	4	0.0024	2.9	4
2-oxoisovalerate:ferredoxin oxidoreductase, delta subunit	TK1979	0.00001	9.1	5	0.0019	17	5
2-oxoisovalerate:ferredoxin oxidoreductase, alpha subunit	TK1980	0.00001	3.5	4	0.00001	53	5
2-oxoisovalerate:ferredoxin oxidoreductase, beta subunit	TK1981	0.00001	4.4	5	0.00001	12	5
Uncharacterized protein	TK0033	0.00081	2.7	4			
Archaeal flagella-related protein D, internal insertion	TK0044	0.00001	11	5			
Manganese-dependent transcription regulator	TK0107	0.046	6	4			
Uncharacterized protein	TK0124	0.03	6.7	4			
Uncharacterized protein	TK0442	0.03	6.7	4			
Chemotaxis histidine kinase, flame shift	TK0634	0.00013	3.9	4			
Uncharacterized protein	TK0648	0.023	2.6	3			
Rubrerythrin-related protein	TK0650	0.0067	3.7	4			
RHH_1 domain-containing protein	TK0695	0.01	∞	5			
Tyrosine recombinase XerA	TK0777	0.04	4	4			
tRNA/rRNA cytosine-C5-methylase, NOL1/NOP2/Sun family	TK0872	0.03	6.7	4			
Hypothetical membrane protein	TK1024	0.017	∞	3			
Uncharacterized protein	TK1025	0.00001	3.6	4			
CDC48/VCP homolog, AAA superfamily	TK1157	0.028	∞	3			
Probable phosphate transport system regulator, fused to TrkA-C domain	TK1457	0.029	2.7	3			
Hydrogenase maturation factor HypA	TK2008	0.011	4	4			
Membrane bound hydrogenase, NiFe-hydrogenase small subunit	TK2089	0.047	∞	2			
Radical_SAM domain-containing protein	TK2160	0.00001	5.9	5			
Uncharacterized protein	TK2283	0.0061	∞	5			
Hydrolase, HAD superfamily	TK0110				0.0075	∞	4
Indolepyruvate oxidoreductase subunit IorA	TK0136				0.036	2.6	3
GMP synthase [glutamine-hydrolyzing] subunit A	TK0190				0.00001	6.3	5
FeS_assembly_P domain-containing protein	TK0527				0.0004	∞	5
Tyrosine--tRNA ligase	TK0568				0.00001	15	5
Acetate--CoA ligase (ADP-forming)	TK0944				0.0004	∞	5
NAD(P)H sulfur oxidoreductase (CoA-dependent)	TK1299				0.046	3.4	3
Uncharacterized protein	TK1329				0.02	∞	4
Predicted dehydrogenase	TK1557				0.046	2.1	3
Glutamine synthetase	TK1796				0.013	3.9	3
Lipoate-protein ligase A, N-terminal section	TK1908				0.00025	∞	5

Pyruvate:ferredoxin oxidoreductase, alpha subunit	TK1983				0.00001	12	5
Pyruvate:ferredoxin oxidoreductase, beta subunit	TK1984				0.00001	44	5

Table S4: Co-purified protein partners of VORβ (TK1981) in sulfur and non-sulfur conditions.

		+S˚			-S˚		
**Co-purifying proteins**	**Gene No.**	**p-value**	**Fold Change**	**Rank**	**p-value**	**Fold Change**	**Rank**
Rubrerythrin-related protein	TK0650	0.00001	∞	5	0.00001	∞	5
Putative snRNP Sm-like protein	TK0976	0.032	2.3	3	0.00001	6.9	5
Pyruvate/ketoisovalerate oxidoreductases common subunit gamma	TK1978	0.00001	67	5	0.00001	47	5
2-oxoisovalerate:ferredoxin oxidoreductase, delta subunit	TK1979	0.00001	∞	5	0.00001	∞	5
2-oxoisovalerate:ferredoxin oxidoreductase, alpha subunit	TK1980	0.00001	200	5	0.00001	180	5
2-oxoisovalerate:ferredoxin oxidoreductase, beta subunit	TK1981	0.00001	∞	5	0.00001	350	5
Probable formate dehydrogenase, alpha subunit	TK2076	0.04	5.5	4			
FeS_assembly_P domain-containing protein	TK0527				0.015	∞	4
Pyruvate:ferredoxin oxidoreductase, alpha subunit	TK1983				0.00001	6.3	5

Table S5: Co-purified protein partners of PORδ (TK1982) in sulfur and non-sulfur conditions.

		+S˚			-S˚		
**Co-purifying proteins**	**Gene No.**	**p-value**	**Fold Change**	**Rank**	**p-value**	**Fold Change**	**Rank**
Uncharacterized protein	TK0483	0.00001	3.2	4	0.00001	56	5
Rubrerythrin domain-containing protein	TK0826	0.00001	87	5	0.00001	∞	5
Putative 5-methylcytosine restriction system, GTPase subunit	TK1009	0.00062	2.3	4	0.00001	140	5
30S ribosomal protein S9	TK1500	0.038	2.3	3	0.017	50	4
Pyruvate/ketoisovalerate oxidoreductases common subunit gamma	TK1978	0.00001	19	5	0.00001	46	5
Pyruvate:ferredoxin oxidoreductase, delta subunit	TK1982	0.00001	66	5	0.00001	∞	5
Pyruvate:ferredoxin oxidoreductase, alpha subunit	TK1983	0.00001	29	5	0.00001	110	5
Pyruvate:ferredoxin oxidoreductase, beta subunit	TK1984	0.00001	56	5	0.00001	270	5
Probable formate dehydrogenase, alpha subunit	TK2076	0.00001	2	4	0.00001	3.6	4
Diadenylate cyclase	TK0510	0.0077	2.3	4			
UPF0284 protein TK0853	TK0853	0.02	6.5	4			
Predicted AP endonuclease	TK1165	0.039	2	3			
Uncharacterized protein	TK1394	0.022	3.4	3			
Putative nickel-responsive regulator	TK1439	0.038	9	4			
Probable tRNA pseudouridine synthase B	TK1509	0.00001	3.6	4			

Uncharacterized protein	TK1631	0.02	6.5	3			
Uncharacterized protein	TK1663	0.031	6	4			
Uncharacterized protein	TK1958	0.00001	∞	5			
2-oxoisovalerate:ferredoxin oxidoreductase, alpha subunit	TK1980	0.0071	2.3	4			
Uncharacterized protein	TK2144	0.046	5.5	4			
Radical_SAM domain-containing protein	TK2160	0.0052	3.5	4			
tRNA-splicing endonuclease	TK2215	0.032	2.4	3			
Indolepyruvate oxidoreductase subunit IorA	TK0136				0.00036	2	4
RecJ-like exonuclease, containing OB-fold nucleic acid-binding domains	TK0155				0.00059	82	5
Pyridoxal 5'-phosphate synthase subunit PdxS	TK0217				0.00001	100	5
Sugar-phosphate nucleotidyltransferase	TK0219				0.00001	38	5
L-aspartate oxidase	TK0297				0.0033	∞	5
Uridylate kinase	TK0305				0.0034	41	5
Elongation factor 1-alpha	TK0308				0.00001	4	5
Uncharacterized protein	TK0438				0.022	∞	4
Uncharacterized protein	TK0453				0.011	∞	4
Predicted GTPase, containing TGS domain	TK0506				0.02	∞	4
Flavin prenyltransferase UbiX	TK0509				0.0086	∞	5
Serine hydroxymethyltransferase	TK0528				0.012	54	4
Peroxiredoxin	TK0537				0.032	∞	4
Metal-dependent phosphohydrolase, HD superfamily	TK0540				0.00001	99	5
Molybdenum cofactor biosynthesis protein B	TK0544				0.00001	2.5	4
Tyrosine--tRNA ligase	TK0568				0.00082	79	5
Uncharacterized protein	TK0592				0.046	∞	4
Uncharacterized protein	TK0593				0.018	∞	4
ABC-type transport system involved in Fe-S cluster assembly, permease component	TK0730				0.003	67	5
tRNA(Met) cytidine acetyltransferase TmcA	TK0754				0.006	∞	5
Toprim domain-containing protein	TK0778				0.0011	2.6	4
2,3-bisphosphoglycerate-independent phosphoglycerate mutase	TK0866				0.0023	2.5	4
Ornithine carbamoyltransferase	TK0871				0.04	∞	4
Uncharacterized protein	TK0883				0.036	∞	4
Pyruvate fromate-lyase activating enzyme-related protein, radical SAM superfamily	TK0893				0.021	48	4
S-layer protein	TK0895				0.005	2.6	4
Predicted ATPase, AAA superfamily, containing PIN and KH nucleic acid-binding domains	TK0953				0.032	44	4
Putative snRNP Sm-like protein	TK0976				0.024	2.5	3
Glycine--tRNA ligase	TK0978				0.02	∞	4

Adenylosuccinate synthetase	TK1002		0.00001	50	5
UDP-glucose 4-epimerase	TK1004		0.029	45	4
D-aminopeptidase	TK1022		0.035	43	4
Uncharacterized protein	TK1025		0.028	∞	4
Protein disulfide oxidoreductase	TK1085		0.027	2.2	3
Translation initiation factor 2 subunit alpha	TK1100		0.024	47	4
Phosphoglycerate kinase	TK1146		0.0054	∞	5
30S ribosomal protein S8e	TK1191		0.00028	2.9	4
Peptide chain release factor subunit 1	TK1239		0.013	53	4
Phosphoenolpyruvate synthase	TK1292		0.032	∞	4
30S ribosomal protein S28e	TK1310		0.001	2.3	4
50S ribosomal protein L7Ae	TK1311		0.0097	2.5	4
DNA primase DnaG	TK1410		0.00001	3.3	4
Cleavage and polyadenylation specficity factor subunit homolog	TK1428		0.00001	40	5
Glutamate dehydrogenase	TK1431		0.00001	2.8	4
Uncharacterized protein	TK1492		0.043	18	4
30S ribosomal protein S13	TK1506		0.012	∞	4
Cytidylate kinase	TK1514		0.016	∞	4
30S ribosomal protein S5	TK1521		0.0066	4.6	5
50S ribosomal protein L3	TK1542		0.018	∞	4
Translation initiation factor 2 subunit beta	TK1621		0.00001	∞	5
S-adenosyl-L-methionine-dependent tRNA 4-demethylwyosine synthase	TK1671		0.022	∞	4
Ferredoxin:NADP oxidoreductase, alpha subunit	TK1684		0.036	∞	4
Vitamin B12-dependent ribonucleotide reductase	TK1736		0.00001	16	5
Predicted transcription regulator, DUF118 helix-turn-helix family	TK1769		0.003	∞	5
Probable tRNA/rRNA methyltransferase	TK1785		0.032	∞	4
Type II/IV secretion system ATPase	TK1853		0.00062	2	4
Translation initiation factor 2 subunit gamma	TK1946		0.00045	23	5
2-oxoisovalerate:ferredoxin oxidoreductase, beta subunit	TK1981		0.021	2.7	3
4Fe-4S cluster-binding protein	TK2077		0.00062	2.8	4
Membrane bound hydrogenase, NiFe-hydrogenase large subunit 2	TK2091		0.029	45	4
Aspartate carbamoyltransferase	TK2196		0.032	29	4
Lysine--tRNA ligase	TK2240		0.021	3.4	3
Proteasome-activating nucleotidase	TK2252		0.018	∞	4
Archaeal histone B	TK2289		0.0081	2.1	4
Anaerobic ribonucleoside-triphosphate reductase	TK2298		0.0018	∞	5

Table S6: Co-purified protein partners of PORα (TK1983) in sulfur and non-sulfur conditions.

		+S°			-S°		
**Co-purifying proteins**	**Gene No.**	**p-value**	**Fold Change**	**Rank**	**p-value**	**Fold Change**	**Rank**
Rubrerythrin domain-containing protein	TK0826	0.00001	53	4	0.00001	∞	5
Uncharacterized protein	TK1958	0.00001	∞	4	0.0013	∞	5
Pyruvate/ketoisovalerate oxidoreductases common subunit gamma	TK1978	0.00001	13	5	0.00001	41	5
Pyruvate:ferredoxin oxidoreductase, delta subunit	TK1982	0.00001	41	4	0.00001	∞	5
Pyruvate:ferredoxin oxidoreductase, alpha subunit	TK1983	0.00001	16	5	0.00001	89	5
Pyruvate:ferredoxin oxidoreductase, beta subunit	TK1984	0.00001	29	5	0.00001	170	5
Flagellin B1	TK0038				0.023	∞	3
Hydrolase, HAD superfamily	TK0110				0.031	∞	3
Aldehyde ferredoxin oxidoreductase	TK0844				0.012	12	4
Predicted AP endonuclease	TK1165				0.028	6.8	4
2-oxoisovalerate:ferredoxin oxidoreductase, alpha subunit	TK1980				0.00001	7.7	5
2-oxoisovalerate:ferredoxin oxidoreductase, beta subunit	TK1981				0.049	4.9	4

Table S7: Co-purified protein partners of PORβ (TK1984) in sulfur and non-sulfur conditions.

		+S°			-S°		
**Co-purifying proteins**	**Gene No.**	**p-value**	**Fold Change**	**Rank**	**p-value**	**Fold Change**	**Rank**
Formate-dependent phosphoribosylglycinamide formyltransferase	TK0207	0.0021	9.3	5	0.00001	72	5
Amidophosphoribosyltransferase	TK0211	0.00001	24	5	0.00001	21	5
Phosphoribosylaminoimidazole-succinocarboxamide synthase	TK0432	0.00001	∞	5	0.00001	∞	5
Rubrerythrin domain-containing protein	TK0826	0.00001	9.9	5	0.00001	∞	5
Predicted dehydrogenase	TK1557	0.00001	3.9	4	0.0067	2	4
Uncharacterized protein	TK1958	0.011	∞	4	0.00001	∞	5
Pyruvate/ketoisovalerate oxidoreductases common subunit gamma	TK1978	0.00001	16	5	0.00001	48	5
Pyruvate:ferredoxin oxidoreductase, delta subunit	TK1982	0.00001	51	5	0.00001	36	5
Pyruvate:ferredoxin oxidoreductase, alpha subunit	TK1983	0.00001	8.9	5	0.00001	37	5
Pyruvate:ferredoxin oxidoreductase, beta subunit	TK1984	0.00001	29	5	0.00001	78	5
Xanthine/uracilpermease	TK0157	0.037	∞	4			
GMP synthase [glutamine-hydrolyzing] subunit B	TK0193	0.00001	8.8	5			
Phosphoribosylamine--glycine ligase	TK0204	0.016	∞	4			
Adenylosuccinate lyase	TK0561	0.009	5.1	5			
Aldehyde ferredoxin oxidoreductase	TK0844	0.0033	5.4	5			

Ferredoxin:NADP oxidoreductase, alpha subunit	TK1325	0.041	7.5	4			
Ferredoxin:NADP oxidoreductase, beta subunit	TK1326	0.025	∞	4			
Archaeal histone A	TK1413	0.0021	∞	4			
Cell division protein FtsZ 1	TK1421	0.0014	21	5			
Glutamine synthetase	TK1796	0.00001	8	5			
Iron-molybdenum cofactor-binding protein	TK2016	0.011	∞	4			
Flagellin B1	TK0038				0.017	12	3
Hydrolase, HAD superfamily	TK0110				0.0005	∞	5
GMP synthase [glutamine-hydrolyzing] subunit A	TK0190				0.00001	5.2	5
ATP-grasp domain-containing protein	TK0203				0.022	∞	3
DUF3216 domain-containing protein	TK0318				0.042	∞	4
Glyceraldehyde 3-phosphate phosphatase	TK0477				0.026	2.3	3
Predicted ATP-dependent endonuclease, OLD family	TK0773				0.035	3.8	3
2-oxoisovalerate:ferredoxin oxidoreductase, beta subunit	TK1981				0.00001	8	5
Uncharacterized protein	TK2105				0.022	∞	4

Table S8: Co-purified protein partners of OGORγ_1 (TK1123) in sulfur and non-sulfur conditions.

		+S°			-S°		
**Co-purifying proteins**	**Gene No.**	**p-value**	**Fold Change**	**Rank**	**p-value**	**Fold Change**	**Rank**
ABC-type iron(III) transport system, periplasmic component	TK0570	0.025	∞	3	0.017	5	4
ABC-type transport system, probable periplasmic component	TK0657	0.04	24	4	0.0027	3	4
Predicted ATP-dependent endonuclease, OLD family	TK0773	0.021	∞	4	0.00001	6.2	5
Putative snRNP Sm-like protein	TK0976	0.00018	2.1	4	0.00001	2.1	4
Uncharacterized protein	TK1046	0.047	∞	3	0.025	3.7	3
Rubrerythrin-related protein	TK1056	0.004	∞	5	0.00001	24	5
2-oxoacid:ferredoxin oxidoreductases, gamma subunit	TK1123	0.00028	∞	5	0.00001	23	5
2-oxoacid:ferredoxin oxidoreductases, beta subunit	TK1124	0.00051	∞	5	0.00001	50	5
2-oxoacid:ferredoxin oxidoreductases, alpha subunit	TK1125	0.00001	∞	5	0.00001	45	5
2-oxoacid:ferredoxin oxidoreductases, alpha subunit	TK1130	0.0049	∞	5	0.00001	5.7	5
2-oxoacid: ferredoxin oxidoreductase, delta subunit	TK1131	0.038	∞	4	0.00001	∞	5
V-type ATP synthase beta chain	TK1603	0.02	28	4	0.027	2.8	3
ABC-type dipeptide/oligopeptide transport system, ATPase component	TK1800	0.024	27	4	0.041	3.3	3
ABC-type dipeptide/oligopeptide transport system, probable periplasmic component	TK1804	0.007	17	5	0.00001	3.5	4
DNA polymerase	TK0001	0.017	∞	2			
N-acetyltransferase, GNAT family	TK0125	0.047	∞	2			
Predicted membrane protease subunit, stomatin/prohibitin homolog	TK0348	0.047	∞	2			

RNA-splicing ligase RtcB	TK0358	0.021	∞	2	
Prolyl endopeptidase	TK0423	0.038	∞	2	
Uncharacterized protein	TK0467	0.047	∞	3	
Aspartate—tRNA(Asp) ligase	TK0492	0.031	∞	2	
Diadenylate cyclase	TK0510	0.038	2	3	
Molybdenum cofactor biosynthesis protein B	TK0544	0.00001	2.6	4	
S-adenosylmethionine synthase	TK0545	0.021	∞	2	
Uncharacterized protein	TK0569	0.0069	34	5	
CDC48/VCP homolog, AAA superfamily	TK0669	0.047	∞	2	
tRNA(Met) cytidine acetyltransferase TmcA	TK0754	0.00001	∞	5	
Aldehyde ferredoxin oxidoreductase	TK0844	0.00034	∞	4	
Ornithine carbamoyltransferase	TK0871	0.025	∞	4	
S-layer protein	TK0895	0.029	7.9	4	
Uncharacterized protein	TK0900	0.038	∞	4	
Predicted ATPase, AAA superfamily, containing PIN and KH nucleic acid-binding domains	TK0953	0.038	∞	2	
DNA-directed RNA polymerase subunit	TK1076	0.0026	∞	4	
30S ribosomal protein S7	TK1077	0.048	23	4	
DNA-directed RNA polymerase subunit beta	TK1083	0.0012	26	4	
Uncharacterized protein	TK1186	0.047	∞	2	
Membrane bound hydrogenase, NiFe-hydrogenase large subunit 2	TK1215	0.00001	∞	3	
Membrane bound hydrogenase, NiFe-hydrogenase large subunit 1	TK1216	0.025	∞	3	
30S ribosomal protein S3Ae	TK1254	0.031	∞	4	
Phosphoenolpyruvate synthase	TK1292	0.0018	∞	4	
Probable translation initiation factor IF-2	TK1305	0.038	∞	3	
Probable glycine dehydrogenase (decarboxylating) subunit 2	TK1379	0.031	∞	2	
DNA primase DnaG	TK1410	0.03	2.8	3	
50S ribosomal protein L1	TK1417	0.031	∞	3	
Probable tRNA pseudouridine synthase B	TK1509	0.034	25	4	
50S ribosomal protein L30	TK1520	0.038	∞	2	
50S ribosomal protein L3	TK1542	0.0012	∞	5	
Predicted dehydrogenase	TK1557	0.019	2.6	3	
V-type ATP synthase alpha chain	TK1602	0.0049	∞	5	
DNA-directed RNA polymerase subunit A’’	TK1699	0.017	∞	3	
Vitamin B12-dependent ribonucleotide reductase	TK1736	0.0011	15	5	
Probable formate dehydrogenase, alpha subunit	TK2076	0.00034	2.5	4	
Membrane bound hydrogenase, NiFe-hydrogenase large subunit 2	TK2091	0.0091	∞	5	
Enolase	TK2106	0.017	∞	4	
Archaeosine synthase subunit alpha	TK2156	0.047	∞	2	

tRNA-splicing endonuclease	TK2215	0.042	15	4			
Non-specific serine/threonine protein kinase	TK2250	0.0038	23	5			
Uncharacterized protein	TK0011				0.00001	2	4
Uncharacterized protein	TK0022				0.032	7	4
Nucleotidyltransferase, fused to N-terminal DNA-binding domain	TK0063				0.0029	2.7	4
Indolepyruvate oxidoreductase subunit IorA	TK0136				0.00001	2.4	4
GMP synthase [glutamine-hydrolyzing] subunit A	TK0190				0.0004	2.7	4
Formate-dependent phosphoribosylglycinamide formyltransferase	TK0207				0.00001	21	5
Amidophosphoribosyltransferase	TK0211				0.00001	4.2	5
Arginase	TK0240				0.022	2.7	3
TatD-related deoxyribonuclease	TK0317				0.007	∞	4
ATPase involved in chromosome partitioning, ParA/MinD family	TK0349				0.029	∞	3
Glyceraldehyde 3-phosphate phosphatase	TK0477				0.00001	2.3	4
Cupin_2 domain-containing protein	TK0503				0.032	7	3
Superoxide reductase	TK0525				0.007	∞	5
Peroxiredoxin	TK0537				0.0051	2.3	4
tRNA(Ile2) 2-agmatinylcytidine synthetase TiaS	TK0553				0.014	2	3
Uncharacterized protein	TK0744				0.018	2.6	3
Putative 5-methylcytosine restriction system, GTPase subunit	TK0795				0.001	5.7	5
Peroxiredoxin, AhpC/TSA family	TK0815				0.007	∞	4
Saccharopine reductase	TK0875				0.00001	∞	5
L-threonine 3-dehydrogenase	TK0916				0.026	2	3
Acetate—CoA ligase (ADP-forming)	TK0944				0.032	7	3
Prefoldin subunit alpha 1	TK1005				0.029	∞	3
2-oxoacid:ferredoxin oxidoreductases, gamma subunit	TK1126				0.00001	30	5
2-oxoacid:ferredoxin oxidoreductases, beta subunit	TK1129				0.00001	7	5
Predicted AP endonuclease	TK1165				0.00001	2	4
Glucosamine-1-phosphate N-acetyltransferase	TK1188				0.015	4	4
50S ribosomal protein L12	TK1415				0.014	2.8	3
NADH:polysulfide oxidoreductase	TK1481				0.02	2.2	3
50S ribosomal protein L18e	TK1502				0.032	7	4
Cysteine synthase	TK1687				0.0051	10	5
FeS_assembly_P domain-containing protein	TK1693				0.0034	∞	5
Xanthine/guanine phosphoribosyltransferase	TK1737				0.0055	6	5
Uncharacterized protein	TK1744				0.00001	∞	5
ABC-type dipeptide/oligopeptide transport system, ATPase component	TK1801				0.042	2.6	3
ABC-type dipeptide/oligopeptide transport system, permease component	TK1803				0.015	4	4

Probable lipoprotein releasing system, ATP-binding protein	TK1859				0.011	2.2	3
Uncharacterized protein	TK2030				0.00083	∞	5
Cytosolic NiFe-hydrogenase, beta subunit	TK2072				0.032	2.2	3
Uncharacterized protein	TK2198				0.022	2.7	3

Table S9: Co-purified protein partners of OGORβ$_2$ (TK1129) in sulfur and non-sulfur conditions.

		+S°			-S°		
**Co-purifying proteins**	**Gene No.**	**p-value**	**Fold Change**	**Rank**	**p-value**	**Fold Change**	**Rank**
Uncharacterized protein	TK0011	0.0033	2.1	4	0.041	4.1	4
Uncharacterized protein	TK0166	0.00001	4.3	5	0.0022	∞	5
Uncharacterized protein	TK0487	0.00001	∞	5	0.00021	∞	4
Metallophosphoesterase, calcineurin superfamily	TK0547	0.00049	2.7	4	0.021	5.7	4
tRNA(Ile2) 2-agmatinylcytidine synthetase TiaS	TK0553	0.00001	2.2	4	0.0034	3.6	4
Uncharacterized protein	TK0930	0.00001	5.5	5	0.028	6.2	4
Cyclic 2,3-diphosphoglycerate synthetase	TK1039	0.00001	2	4	0.018	3.5	3
Rubrerythrin-related protein	TK1056	0.001	2.1	4	0.0021	8.9	5
2-oxoacid:ferredoxin oxidoreductases, gamma subunit	TK1126	0.00001	18	5	0.00001	∞	5
2-oxoacid:ferredoxin oxidoreductases, beta subunit	TK1129	0.00001	7.6	5	0.00001	41	5
2-oxoacid:ferredoxin oxidoreductases, alpha subunit	TK1130	0.00001	5.2	5	0.00001	39	5
2-oxoacid: ferredoxin oxidoreductase, delta subunit	TK1131	0.00001	3.4	4	0.00017	11	5
tRNA (1-methyladenosine) methyltransferase	TK1328	0.00001	∞	3	0.023	13	4
Uncharacterized protein	TK1463	0.00001	8.3	5	0.048	11	4
tRNA-splicing endonuclease	TK2215	0.00001	2.3	4	0.0044	4.2	5
Uncharacterized protein	TK0022	0.021	2.5	3			
Hypothetical membrane protein, conserved	TK0025	0.021	4.5	4			
Hypothetical membrane protein, conserved	TK0035	0.023	∞	3			
TATA-box-binding protein	TK0132	0.037	4	4			
Xanthine/uracilpermease	TK0157	0.0065	9	5			
Hypothetical membrane protein	TK0162	0.023	∞	4			
S-layer-like array protein	TK0164	0.00001	∞	5			
Amidophosphoribosyltransferase	TK0211	0.00037	2.2	4			
Probable formate dehydrogenase, alpha subunit	TK0214	0.049	∞	3			
Imidazoleglycerol-phosphate dehydratase	TK0245	0.023	∞	4			
Phosphonate 12etabolism protein PhnP homolog, metallo-beta-lactamase superfamily	TK0288	0.0098	4	5			
Predicted membrane protease subunit, stomatin/prohibitin homolog	TK0348	0.021	2.1	3			
Protein archease	TK0361	0.011	∞	3			

Uncharacterized protein	TK0442	0.029	3.3	3	
Acetyl-CoA synthetase I (NDP forming), beta subunit	TK0465	0.023	∞	4	
Uncharacterized protein	TK0467	0.00001	6.9	5	
Hypothetical membrane protein, conserved, containing DUF11 domain	TK0493	0.00029	6	5	
Uncharacterized protein	TK0569	0.00001	2.5	4	
ABC-type iron(III) transport system, periplasmic component	TK0570	0.003	2.5	4	
Metallophosphoesterase, calcineurin superfamily	TK0574	0.00001	3.4	4	
Hypothetical membrane protein	TK0596	0.049	∞	4	
ABC-type transport system, probable periplasmic component	TK0657	0.00001	3.2	4	
ABC-type multidrug transport system, ATPase component	TK0694	0.049	∞	4	
ATPase, ParA/MinD family, containing ferredoxin domains	TK0701	0.049	∞	3	
ABC-type iron(III)-siderophore transport system, periplasmic component fused to N-terminal uncharacterized domain	TK0706	0.00001	3.8	4	
ABC-type molybdate transport system, periplasmic component	TK0717	0.011	∞	4	
ABC-type molybdate transport system, permease component	TK0718	0.022	3	3	
Iron-molybdenum cofactor-binding protein	TK0724	0.045	6	4	
Glycerol-1-phosphate dehydrogenase [NAD(P)+]	TK0789	0.00062	2.7	4	
Oligosaccharyl transferase	TK0810	0.00043	13	5	
Probable vitamin B12 transport protein	TK0865	0.00098	7	5	
Uncharacterized protein	TK0879	0.012	5	4	
ABC-type multidrug transport system, ATPase component	TK0942	0.023	∞	3	
50S ribosomal protein L37e	TK0975	0.049	∞	4	
Fructose-bisphosphate aldolase class 1	TK0989	0.018	2	3	
Oxaloacetate decarboxylase, alpha subunit	TK0990	0.012	5	4	
Hypothetical membrane protein	TK1024	0.049	∞	4	
Predicted acetyltransferase, isoleucine patch superfamily	TK1174	0.0063	5.5	5	
Membrane bound hydrogenase, MbxH' subunit	TK1219	0.012	5	4	
Uncharacterized protein	TK1245	0.0083	2.8	4	
Archaeal Lon protease	TK1264	0.049	∞	3	
Hypothetical membrane protein, conserved	TK1302	0.023	∞	4	
Cell division protein FtsZ 1	TK1421	0.0051	3	4	
Ribonuclease J	TK1469	0.00001	28	3	
Protein translocase subunit SecY	TK1518	0.0023	3.6	4	
50S ribosomal protein L18	TK1522	0.021	2.5	3	
Hypothetical membrane protein, conserved	TK1577	0.0017	11	5	
ABC-type multidrug transport system, ATPase component	TK1579	0.00001	5.4	5	
Protein-export membrane protein SecF	TK1593	0.0033	10	5	
Archaeal/vacuolar-type H+-ATPase, subunit H	TK1596	0.013	3.2	3	

V-type ATP synthase subunit I	TK1597	0.0018	6.5	5			
V-type ATP synthase alpha chain	TK1602	0.00075	3.1	4			
V-type ATP synthase beta chain	TK1603	0.0018	2.4	4			
V-type ATP synthase subunit D	TK1604	0.021	4.5	4			
Methylmalonyl-CoA decarboxylase, alpha subunit	TK1622	0.00001	3.6	4			
Exosome complex component Rrp41	TK1634	0.0033	10	5			
Glycosyltransferase, family 4	TK1723	0.049	∞	3			
Xanthine/guanine phosphoribosyltransferase	TK1737	0.00015	5.2	5			
Amylopullulanase, GH57 family	TK1774	0.0063	5.5	5			
ABC-type dipeptide/oligopeptide transport system, permease component	TK1802	0.00038	2.6	4			
ABC-type dipeptide/oligopeptide transport system, permease component	TK1803	0.00045	3.2	4			
Peptidase_M1 domain-containing protein	TK1873	0.049	∞	4			
Carbon-nitrogen hydrolase	TK1916	0.0051	∞	3			
tRNA/rRNA cytosine-C5-methylase, NOL1/NOP2/Sun family	TK1935	0.00001	6.2	5			
Uncharacterized protein	TK1950	0.0017	11	5			
Uncharacterized protein	TK1953	0.045	6	4			
Hypothetical membrane protein, conserved, containing TPR-repeat domains	TK1972	0.00043	13	4			
Ferredoxin 3	TK2012	0.00015	2.8	4			
Membrane bound hydrogenase, MbhC subunit	TK2082	0.037	4	4			
Membrane bound hydrogenase, MbhH subunit	TK2087	0.017	3.7	3			
Membrane bound hydrogenase, MbhI subunit	TK2088	0.0051	∞	4			
Membrane bound hydrogenase, NiFe-hydrogenase small subunit	TK2089	0.00015	2.8	4			
Membrane bound hydrogenase, 4Fe-4S cluster-binding subunit	TK2093	0.00053	2	4			
Methyl-accepting chemotaxis protein	TK2147	0.011	∞	4			
ABC-type multidrug transport system, ATPase component	TK2161	0.049	∞	3			
Hypothetical membrane protein	TK2201	0.049	∞	3			
Proteasome subunit beta 2	TK2207	0.049	∞	4			
Predicted permease, major facilitator superfamily	TK2266	0.049	∞	4			
Uncharacterized protein	TK2283	0.0033	10	5			
30S ribosomal protein S17e	TK2292	0.048	3	3			
Indolepyruvate oxidoreductase subunit IorA	TK0136				0.022	4.2	4
UPF0173 metal-dependent hydrolase TK0141	TK0141				0.0062	6	5
AMP phosphorylase	TK0352				0.00011	3	4
Uncharacterized protein	TK0483				0.00001	8.1	5
Uncharacterized protein	TK0519				0.0077	9.7	5
Molybdenum cofactor biosynthesis protein B	TK0544				0.00001	3.8	4

NAD-dependent protein deacylase	TK0685				0.037	7.3	4
Molybdopterin oxidoreductase, molybdopterin-binding subunit	TK0690				0.046	7	4
ABC-type transport system involved in Fe-S cluster assembly, ATPase component	TK0731				0.017	3.9	3
Predicted ATP-dependent endonuclease, OLD family	TK0773				0.00022	4.8	5
Putative 5-methylcytosine restriction system, GTPase subunit	TK0795				0.028	6.2	4
Type 2 DNA topoisomerase 6 subunit B	TK0799				0.0026	3	4
Uncharacterized protein	TK0900				0.027	3.4	3
Putative 5-methylcytosine restriction system, GTPase subunit	TK1009				0.002	3.1	4
Large helicase-related protein	TK1015				0.00001	5.9	5
Ribonuclease Z	TK1114				0.012	5.1	4
Site-specific DNA-methyltransferase (adenine-specific)	TK1158				0.047	3.7	3
Uncharacterized protein	TK1304				0.03	∞	4
Predicted ATPase, AAA superfamily	TK1314				0.026	3.9	3
Uncharacterized protein	TK1329				0.031	3.4	3
Uncharacterized protein	TK1376				0.023	∞	4
Uncharacterized protein	TK1394				0.0076	5.9	5
Probable tRNA pseudouridine synthase B	TK1509				0.0003	4.2	5
Predicetd ATPase	TK1549				0.012	3	3
Ribosome maturation protein SDO1 homolog	TK1636				0.048	2.6	3
Uncharacterized protein	TK1663				0.00067	6.8	5
Cysteine synthase	TK1687				0.041	∞	4
Vitamin B12-dependent ribonucleotide reductase	TK1736				0.00001	2.6	4
DNA polymerase II large subunit	TK1903				0.025	2.8	3
Uncharacterized protein	TK2030				0.03	∞	4
Radical_SAM domain-containing protein	TK2160				0.019	6.7	4
LTD domain-containing protein	TK2178				0.041	∞	3
Non-specific serine/threonine protein kinase	TK2250				0.027	3	3
Anaerobic ribonucleoside-triphosphate reductase	TK2298				0.026	3.9	3

Table S10: Co-purified protein partners of OGORα_2 (TK1130) in sulfur and non-sulfur conditions.

		+S°			-S°		
**Co-purifying proteins**	**Gene No.**	**p-value**	**Fold Change**	**Rank**	**p-value**	**Fold Change**	**Rank**
Rubrerythrin-related protein	TK1056	0.0088	2.2	4	0.0077	7.6	5
2-oxoacid:ferredoxin oxidoreductases, gamma subunit	TK1126	0.00001	11	5	0.00001	∞	5
2-oxoacid:ferredoxin oxidoreductases, beta subunit	TK1129	0.00001	5.6	5	0.00001	22	5
2-oxoacid:ferredoxin oxidoreductases, alpha subunit	TK1130	0.00001	3.4	4	0.00001	22	5

Uncharacterized protein	TK0124	0.043	2.8	3			
Rubrerythrin-related protein, fused to C-terminal DUF835 domain	TK0316	0.019	2.6	3			
Methylmalonyl-CoA epimerase	TK0330	0.041	4.5	4			
Superoxide reductase	TK0525	0.043	7	4			
tRNA(Ile2) 2-agmatinylcytidine synthetase TiaS	TK0553	0.007	1.8	4			
Uncharacterized protein	TK0648	0.027	2.2	3			
Acetate—CoA ligase (ADP-forming)	TK0665	0.00018	2.8	4			
Uncharacterized protein	TK0733	0.037	∞	4			
Uncharacterized protein	TK0900	0.036	1.8	3			
TPR_REGION domain-containing protein	TK0947	0.009	6	5			
2-oxoacid:ferredoxin oxidoreductases, beta subunit	TK1124	0.048	1.8	3			
2-oxoacid: ferredoxin oxidoreductase, delta subunit	TK1131	0.0046	2.8	4			
Uncharacterized protein	TK1202	0.048	1.8	3			
1,4-alpha-glucan branching enzyme TK1436	TK1436	0.033	3.2	3			
Radical_SAM domain-containing protein	TK2160	0.00036	2.1	4			
UPF0173 metal-dependent hydrolase TK0141	TK0141				0.013	5.5	4
RNA-splicing ligase RtcB	TK0358				0.0072	4.2	5
Uncharacterized protein	TK0483				0.0017	4.3	5
Predicted ATP-dependent endonuclease, OLD family	TK0773				0.0085	3.8	4
Type 2 DNA topoisomerase 6 subunit B	TK0799				0.036	2.6	3
Uncharacterized protein	TK1376				0.04	∞	4
50S ribosomal protein L12	TK1415				0.046	11	4
Uncharacterized protein	TK1430				0.025	3.2	3
30S ribosomal protein S8	TK1526				0.014	6	4
Vitamin B12-dependent ribonucleotide reductase	TK1736				0.0056	2.3	4
Predicted transcription regulator, DUF118 helix-turn-helix family	TK1769				0.0047	4.2	5
Probable formate dehydrogenase, alpha subunit	TK2076				0.04	2.3	3

Table S11: Co-purified protein partners of IORβ (TK0135) in sulfur and non-sulfur conditions.

		+S˚			-S˚		
Co-purifying proteins	Gene No.	p-value	Fold Change	Rank	p-value	Fold Change	Rank
Indolepyruvate oxidoreductase subunit IorB	TK0135	0.00001	72	5	0.00001	29	5
Indolepyruvate oxidoreductase subunit IorA	TK0136	0.00001	190	5	0.00001	96	5
2-dehydro-3-deoxyphosphoheptonate aldolase	TK0268	0.00001	∞	5	0.00001	∞	5
Aldehyde ferredoxin oxidoreductase	TK0844	0.0048	31	5	0.049	5	4

Cytosolic NiFe-hydrogenase, beta subunit	TK2072	0.0061	∞	5	0.012	13	3
Indolepyruvate: ferredoxin oxidoreductase, beta subunit	TK2244	0.00001	∞	5	0.00001	∞	5
tRNA/rRNA cytosine-C5-methylase, NOL1/NOP2/Sun family, fused to N-terminal NusB regulator domain	TK2304	0.00048	∞	3	0.035	5.3	2
UPF0173 metal-dependent hydrolase TK0141	TK0141	0.026	9.1	4			
RecJ-like exonuclease, containing OB-fold nucleic acid-binding domains	TK0155	0.0024	21	5			
AMP phosphorylase	TK0352	0.00001	2.2	4			
Uncharacterized protein	TK0483	0.00065	40	5			
Serine hydroxymethyltransferase	TK0528	0.00087	39	5			
Peroxiredoxin	TK0537	0.022	∞	4			
Metal-dependent phosphohydrolase, HD superfamily	TK0540	0.041	∞	3			
Proline--tRNA ligase	TK0550	0.033	21	4			
Uncharacterized protein	TK0569	0.032	9.7	4			
Acetate--CoA ligase (ADP-forming)	TK0665	0.00066	∞	5			
Type 2 DNA topoisomerase 6 subunit B	TK0799	0.0015	12	5			
D-aminopeptidase	TK1022	0.016	∞	4			
30S ribosomal protein S7	TK1077	0.023	15	4			
Phosphoenolpyruvate synthase	TK1292	0.0012	∞	5			
NAD(P)H sulfur oxidoreductase (CoA-dependent)	TK1299	0.009	12	5			
Uncharacterized protein	TK1394	0.03	∞	4			
DNA primase DnaG	TK1410	0.0045	2.1	4			
Cleavage and polyadenylation specificity factor subunit homolog	TK1428	0.00089	18	5			
Phosphopyruvate hydratase	TK1497	0.038	13	4			
Ribosome maturation protein SDO1 homolog	TK1636	0.03	14	4			
Ferredoxin:NADP oxidoreductase, alpha subunit	TK1684	0.041	∞	4			
Vitamin B12-dependent ribonucleotide reductase	TK1736	0.0061	6	5			
Predicted hydrolase, metallo-beta-lactamase superfamily	TK1778	0.03	∞	3			
DNA polymerase II large subunit	TK1903	0.0066	8.8	5			
Translation initiation factor 2 subunit gamma	TK1946	0.049	6.3	4			
4Fe-4S cluster-binding protein	TK2077	0.022	2	3			
cobW domain-containing protein	TK2200	0.028	6.1	4			
tRNA-splicing endonuclease	TK2215	0.03	14	4			
Lysine--tRNA ligase	TK2240	0.0029	13	5			
Flagellin B2	TK0039				0.0035	16	4
Flagellin B3	TK0040				0.022	∞	4
Acetate--CoA ligase (ADP-forming)	TK0944				0.014	∞	4
Phosphoenolpyruvate carboxykinase [GTP]	TK1405				0.028	7	4
Probable tRNA/rRNA methyltransferase	TK1785				0.0054	∞	3

tRNA/rRNA cytosine-C5-methylase, NOL1/NOP2/Sun family	TK1935				0.0033	∞	3

Table S12: Co-purified protein partners of IORα (TK1036) in sulfur and non-sulfur conditions.

		+S°			-S°		
**Co-purifying proteins**	**Gene No.**	**p-value**	**Fold Change**	**Rank**	**p-value**	**Fold Change**	**Rank**
Indolepyruvate oxidoreductase subunit IorB	TK0135	0.013	8.7	4	0.028	3.2	3
Indolepyruvate oxidoreductase subunit IorA	TK0136	0.00001	140	5	0.00001	66	5
Peroxiredoxin	TK0537	0.00001	∞	5	0.00071	6.2	4
Acetate--CoA ligase (ADP-forming)	TK0665	0.00001	∞	5	0.00034	9	5
Acetate--CoA ligase (ADP-forming)	TK0944	0.00001	∞	5	0.00001	∞	4
Phosphoenolpyruvate synthase	TK1292	0.0032	∞	5	0.048	4.7	3
V4R domain-containing protein	TK1312	0.0023	∞	5	0.048	∞	2
Phosphoenolpyruvate carboxykinase [GTP]	TK1405	0.00001	∞	5	0.00046	11	4
Acetate--CoA ligase (ADP-forming)	TK1880	0.00001	∞	5	0.0023	∞	3
SAM-dependent methyltransferase, UPF0020 family	TK2045	0.00001	∞	3	0.0023	∞	3
Cytosolic NiFe-hydrogenase, beta subunit	TK2072	0.0032	∞	5	0.0022	16	4
Probable formate dehydrogenase, alpha subunit	TK2076	0.0045	4.4	5	0.021	1.3	3
Indolepyruvate: ferredoxin oxidoreductase, beta subunit	TK2244	0.00001	∞	5	0.00001	∞	5
Myo-inositol-1-phosphate synthase	TK2278	0.00001	∞	5	0.00001	∞	3
RecJ-like exonuclease, containing OB-fold nucleic acid-binding domains	TK0155	0.0019	20	5			
Quinolinate synthase A	TK0296	0.0089	∞	5			
Serine hydroxymethyltransferase	TK0528	0.0011	21	5			
DEAD/DEAH box RNA helicase	TK0566	0.012	∞	2			
Type 2 DNA topoisomerase 6 subunit B	TK0799	0.00047	11	5			
Aldehyde ferredoxin oxidoreductase	TK0844	0.0043	29	5			
Predicted ATPase, AAA superfamily, containing PIN and KH nucleic acid-binding domains	TK0953	0.012	∞	4			
Glycine--tRNA ligase	TK0978	0.017	∞	4			
N2, N2-dimethylguanosine tRNA methyltransferase	TK0981	0.039	7	4			
D-aminopeptidase	TK1022	0.034	∞	4			
Methionine--tRNA ligase	TK1049	0.0089	∞	5			
DNA-directed RNA polymerase subunit	TK1076	0.00026	25	5			
30S ribosomal protein S7	TK1077	0.0057	17	5			
DNA-directed RNA polymerase subunit beta	TK1083	0.035	6.8	4			
Predicted AP endonuclease	TK1165	0.029	5.9	4			
Peptide chain release factor subunit 1	TK1239	0.017	∞	4			
Probable translation initiation factor IF-2	TK1305	0.019	23	4			

Uncharacterized protein	TK1329	0.034	20	4			
Uncharacterized protein	TK1394	0.034	∞	4			
DNA primase DnaG	TK1410	0.0068	2	4			
Cleavage and polyadenylation specficity factor subunit homolog	TK1428	0.00081	17	5			
Glutamate dehydrogenase	TK1431	0.024	2.3	3			
Leucine--tRNA ligase	TK1461	0.024	∞	4			
Probable tRNA pseudouridine synthase B	TK1509	0.022	14	4			
Adenylate kinase	TK1517	0.034	∞	4			
30S ribosomal protein S17	TK1532	0.036	10	4			
Predicted metal-dependent hydrolase	TK1611	0.034	20	4			
Uncharacterized protein	TK1626	0.034	9	4			
Ribosome maturation protein SDO1 homolog	TK1636	0.0019	20	5			
Vitamin B12-dependent ribonucleotide reductase	TK1736	0.015	5.2	4			
DNA polymerase II large subunit	TK1903	0.00001	16	5			
Pyruvate:ferredoxin oxidoreductase, alpha subunit	TK1983	0.0034	2	4			
Fructose-1,6-bisphosphate aldolase/phosphatase	TK2164	0.0023	∞	5			
tRNA-splicing endonuclease	TK2215	0.036	13	4			
Lysine--tRNA ligase	TK2240	0.0088	11	5			
Thermosome subunit beta	TK2303	0.032	2	3			
TATA-box-binding protein	TK0132				0.048	∞	2
GMP synthase [glutamine-hydrolyzing] subunit A	TK0190				0.046	3.3	3

Table S13: Co-purified protein partners of FDHγ_2 (TK2075) in sulfur and non-sulfur conditions.

		$+S^{\circ}$			$-S^{\circ}$		
**Co-purifying proteins**	**Gene No.**	**p-value**	**Fold Change**	**Rank**	**p-value**	**Fold Change**	**Rank**
Indolepyruvate oxidoreductase subunit IorA	TK0136	0.00045	14	5	0.00001	3.7	4
Predicted glutamine amidotransferase, class II	TK1035	0.047	11	4	0.024	2.7	3
2-oxoacid:ferredoxin oxidoreductases, alpha subunit	TK1130	0.033	12	4	0.00099	3.9	4
Predicted AP endonuclease	TK1165	0.00001	5.5	5	0.00001	3.3	4
Probable lipoprotein releasing system, ATP-binding protein	TK1859	0.0019	12	5	0.00001	4.2	5
Cytosolic NiFe-hydrogenase, beta subunit	TK2072	0.0098	∞	4	0.0025	4	5
Glutamate synthase beta chain-related oxidoreductase	TK2074	0.00001	68	5	0.00001	20	5
4Fe-4S cluster-binding protein	TK2075	0.00001	45	5	0.00001	18	5
4Fe-4S cluster-binding protein	TK2077	0.00001	4	5	0.00036	2.1	4
Flagellin B1	TK0038	0.00092	13	4			
Archaeal flagella-related protein D, internal insertion	TK0044	0.00001	∞	5			
UPF0173 metal-dependent hydrolase TK0141	TK0141	0.027	4	4			

Flavin prenyltransferase UbiX	TK0509	0.033	12	4			
Iron-molybdenum cofactor-binding protein	TK0732	0.023	∞	4			
Aldehyde ferredoxin oxidoreductase	TK0844	0.0085	7.7	5			
Probable translation initiation factor IF-2	TK1305	0.029	3.3	3			
Ferredoxin:NADP oxidoreductase, alpha subunit	TK1325	0.0028	∞	5			
2-oxoisovalerate:ferredoxin oxidoreductase, alpha subunit	TK1980	0.021	6.7	4			
2-oxoisovalerate:ferredoxin oxidoreductase, beta subunit	TK1981	0.035	∞	4			
Probable formate dehydrogenase, alpha subunit	TK2076	0.00001	3.6	4			
4Fe-4S cluster-binding protein	TK2078	0.0094	5	5			
Uncharacterized protein	TK0011				0.0013	2.1	4
Nucleotidyltransferase, fused to N-terminal DNA-binding domain	TK0063				0.002	3.5	4
TATA-box-binding protein	TK0132				0.00025	∞	5
Indolepyruvate oxidoreductase subunit IorB	TK0135				0.00099	3.9	4
Uncharacterized protein	TK0154				0.049	∞	3
GMP synthase [glutamine-hydrolyzing] subunit A	TK0190				0.014	2.7	3
Arginase	TK0240				0.0011	4.7	5
Elongation factor 2	TK0309				0.0065	3.6	4
TatD-related deoxyribonuclease	TK0317				0.023	∞	3
Predicted membrane protease subunit, stomatin/prohibitin homolog	TK0348				0.049	4	4
Acetyl-CoA synthetase I (NDP forming), beta subunit	TK0465				0.049	∞	4
Glyceraldehyde 3-phosphate phosphatase	TK0477				0.00037	2.5	4
Diadenylate cyclase	TK0510				0.004	2	4
Peroxiredoxin	TK0537				0.00001	6	5
tRNA(Ile2) 2-agmatinylcytidine synthetase TiaS	TK0553				0.00016	3.7	4
Acetate--CoA ligase (ADP-forming)	TK0665				0.014	2.7	3
CDC48/VCP homolog, AAA superfamily	TK0669				0.032	2.7	3
Molybdopterin oxidoreductase, molybdopterin-binding subunit	TK0690				0.00098	7.1	5
Uncharacterized protein	TK0744				0.017	3.7	3
tRNA(Met) cytidine acetyltransferase TmcA	TK0754				0.019	2	3
Glycerol-1-phosphate dehydrogenase [NAD(P)+]	TK0789				0.014	3	3
UPF0179 protein TK0790	TK0790				0.006	2.2	4
Type 2 DNA topoisomerase 6 subunit A	TK0798				0.0024	2.1	4
Peroxiredoxin, AhpC/TSA family	TK0815				0.049	∞	4
S-layer protein	TK0895				0.00001	2.4	4
N2, N2-dimethylguanosine tRNA methyltransferase	TK0981				0.00001	2.9	4
Protein disulfide oxidoreductase	TK1085				0.00001	3.8	4
Ribosome biogenesis protein Nop10	TK1101				0.045	8	4
2-oxoacid:ferredoxin oxidoreductases, gamma subunit	TK1126				0.0011	∞	5

2-oxoacid:ferredoxin oxidoreductases, beta subunit	TK1129				0.033	3.1	3
Glucosamine-1-phosphate N-acetyltransferase	TK1188				0.00098	7.1	5
DNA helicase	TK1199				0.029	4.4	4
NAD(P)H sulfur oxidoreductase (CoA-dependent)	TK1299				0.00001	3.5	4
50S ribosomal protein L12	TK1415				0.00056	4.6	5
Glutamate dehydrogenase	TK1431				0.00001	2.2	4
NADH:polysulfide oxidoreductase	TK1481				0.00001	4.1	5
Probable tRNA pseudouridine synthase B	TK1509				0.00065	2.3	4
50S ribosomal protein L14e	TK1513				0.029	4.4	4
V-type ATP synthase subunit I	TK1597				0.049	∞	4
V-type ATP synthase alpha chain	TK1602				0.0065	3.6	4
Cysteine synthase	TK1687				0.00001	48	5
FeS_assembly_P domain-containing protein	TK1693				0.049	∞	3
Xanthine/guanine phosphoribosyltransferase	TK1737				0.0065	12	5
ABC-type dipeptide/oligopeptide transport system, ATPase component	TK1800				0.0032	6.2	5
ABC-type dipeptide/oligopeptide transport system, ATPase component	TK1801				0.037	3.7	3
ABC-type dipeptide/oligopeptide transport system, probable periplasmic component	TK1804				0.044	2	3
Peptidyl-prolyl cis-trans isomerase	TK1850				0.00012	∞	5
Type II/IV secretion system ATPase	TK1853				0.00001	3.2	4
DNA polymerase II large subunit	TK1903				0.00001	2.4	4
Uncharacterized protein	TK2030				0.0051	∞	5
dITP/XTP pyrophosphatase	TK2111				0.00001	∞	3
tRNA-splicing endonuclease	TK2215				0.00039	2.3	4
Beta-ribofuranosylaminobenzene 5'-phosphate synthase	TK2242				0.023	∞	4
Uncharacterized protein	TK2283				0.012	6.7	4

Table S14: Co-purified protein partners of FDHα (TK2076) in sulfur and non-sulfur conditions.

		+S°			-S°		
**Co-purifying proteins**	**Gene No.**	**p-value**	**Fold Change**	**Rank**	**p-value**	**Fold Change**	**Rank**
Glycerol-1-phosphate dehydrogenase [NAD(P)+]	TK0789	0.0083	10	5	0.002	3	4
L-threonine 3-dehydrogenase	TK0916	0.0032	5.4	5	0.00041	2.4	4
Predicted AP endonuclease	TK1165	0.00001	4	5	0.00001	2.1	4
Proteasome subunit alpha	TK1637	0.032	6.3	4	0.0007	3	4
Probable formate dehydrogenase, alpha subunit	TK2076	0.00001	4.5	5	0.00001	2.4	4
UPF0173 metal-dependent hydrolase TK0141	TK0141	0.023	3.9	3			

Pyridoxal 5'-phosphate synthase subunit PdxS	TK0217	0.048	3.2	3	
L-aspartate oxidase	TK0297	0.011	6.5	4	
Uncharacterized protein	TK0440	0.042	5.2	4	
PDDEXK_1 domain-containing protein	TK0446	0.011	∞	4	
Uncharacterized protein	TK0453	0.025	5.7	3	
Arginase	TK0474	0.0029	19	4	
Uncharacterized protein	TK0483	0.029	3.2	3	
Flavin prenyltransferase UbiX	TK0509	0.00066	23	4	
Adenylosuccinate lyase	TK0561	0.029	4.4	4	
Tyrosine--tRNA ligase	TK0568	0.016	9	3	
ABC-type iron(III) transport system, ATPase component	TK0572	0.041	7.5	4	
Metallophosphoesterase, calcineurin superfamily	TK0574	0.024	∞	3	
Uncharacterized protein	TK0592	0.037	∞	3	
Acetate--CoA ligase (ADP-forming)	TK0665	0.018	14	4	
Thermosome subunit alpha	TK0678	0.021	2.8	3	
tRNA(Met) cytidine acetyltransferase TmcA	TK0754	0.014	4.6	4	
Type 2 DNA topoisomerase 6 subunit B	TK0799	0.00096	3.4	4	
Type A flavoprotein	TK0814	0.041	2.8	3	
Aldehyde ferredoxin oxidoreductase	TK0844	0.0049	6.4	5	
Predicted ATPase, AAA superfamily, containing PIN and KH nucleic acid-binding domains	TK0953	0.0028	6.8	5	
Adenylosuccinate synthetase	TK1002	0.00022	6.6	5	
Predicted glutamine amidotransferase, class II	TK1035	0.012	15	4	
Metallophosphoesterase, calcineurin superfamily	TK1037	0.018	14	4	
DNA topoisomerase 1	TK1091	0.025	13	3	
Translation initiation factor 2 subunit alpha	TK1100	0.024	5.2	4	
2-oxoacid:ferredoxin oxidoreductases, alpha subunit	TK1125	0.036	12	3	
Probable translation initiation factor IF-2	TK1305	0.021	3.2	3	
Uncharacterized protein	TK1329	0.013	4.4	4	
Leucine--tRNA ligase	TK1461	0.04	4.8	4	
Cytidylate kinase	TK1514	0.037	∞	2	
Adenylate kinase	TK1517	0.0051	5.8	5	
50S ribosomal protein L2	TK1539	0.032	5.5	4	
Predicetd ATPase	TK1549	0.017	2.8	3	
tRNA uridine(34) acetyltransferase	TK1574	0.036	12	3	
DNA-directed RNA polymerase subunit A''	TK1699	0.022	8.5	4	
Sugar-phosphate nucleotydyltransferase	TK1711	0.011	∞	3	
Vitamin B12-dependent ribonucleotide reductase	TK1736	0.00001	3.3	4	
Predicted transcription regulator, DUF118 helix-turn-helix family	TK1769	0.00059	∞	4	

Predicted hydrolase, metallo-beta-lactamase superfamily	TK1778	0.0011	13	5			
Carboxymuconolactone decarboxylase-related protein	TK1974	0.016	∞	4			
2-oxoisovalerate:ferredoxin oxidoreductase, alpha subunit	TK1980	0.0084	3.3	4			
2-oxoisovalerate:ferredoxin oxidoreductase, beta subunit	TK1981	0.00038	6.4	5			
Hydrogenase expression/formation protein HypE	TK1993	0.037	∞	3			
Carbamoyltransferase	TK1997	0.016	9	3			
Iron-molybdenum cofactor-binding protein	TK2016	0.037	∞	3			
ATPase, RecA superfamily	TK2042	0.011	6.5	4			
Uncharacterized protein	TK2148	0.041	7.5	4			
Tungsten-containing glyceraldehyde-3-phosphate:ferredoxin oxidoreductase	TK2163	0.0031	∞	4			
Aspartate carbamoyltransferase regulatory chain	TK2195	0.024	∞	3			
Aspartate carbamoyltransferase	TK2196	0.018	3.7	3			
Replication factor C small subunit	TK2218	0.0021	7	4			
Non-specific serine/threonine protein kinase	TK2250	0.0014	3.7	4			
Proteasome-activating nucleotidase	TK2252	0.0045	7.2	5			
Deoxycytidylate deaminase	TK2257	0.025	13	4			
Uncharacterized protein	TK2269	0.037	∞	2			
Uncharacterized protein	TK2283	0.036	12	4			
Anaerobic ribonucleoside-triphosphate reductase	TK2298	0.049	4.3	4			
GMP synthase [glutamine-hydrolyzing] subunit A	TK0190				0.0044	2.5	4
Formate-dependent phosphoribosylglycinamide formyltransferase	TK0207				0.0043	8	3
Amidophosphoribosyltransferase	TK0211				0.0037	2	4
Metallophosphoesterase, calcineurin superfamily	TK0547				0.009	3.3	4
DNA/RNA-binding protein Alba	TK0560				0.009	3.3	4
Predicted ATP-dependent endonuclease, OLD family	TK0773				0.00028	3.6	4
N2, N2-dimethylguanosine tRNA methyltransferase	TK0981				0.00001	2	4
Fructose-bisphosphate aldolase class 1	TK0989				0.011	2.8	3
Uncharacterized protein	TK1046				0.0068	4.5	5
Transcription initiation factor IIB 1	TK1280				0.028	3.5	3
50S ribosomal protein L12	TK1415				0.0024	2.7	4
50S ribosomal protein L18e	TK1502				0.044	5	4
Protein translocase subunit SecY	TK1518				0.00001	∞	5
V-type ATP synthase alpha chain	TK1602				0.0046	3.7	4
V-type ATP synthase beta chain	TK1603				0.00038	11	5
Radical_SAM domain-containing protein	TK1766				0.0046	3.7	4
ABC-type dipeptide/oligopeptide transport system, ATPase component	TK1800				0.003	3.2	4
ABC-type dipeptide/oligopeptide transport system, ATPase component	TK1801				0.012	2.4	3

ABC-type dipeptide/oligopeptide transport system, permease component	TK1803				0.011	∞	4
Type II/IV secretion system ATPase	TK1853				0.00001	2.2	4
Membrane bound hydrogenase, 4Fe-4S cluster-binding subunit	TK2093				0.0096	7	5

Table S15: Co-purified protein partners of $FDH\gamma_3$ (TK2077) in sulfur and non-sulfur conditions.

		+S˚			-S˚		
**Co-purifying proteins**	**Gene No.**	**p-value**	**Fold Change**	**Rank**	**p-value**	**Fold Change**	**Rank**
Nucleotidyltransferase, fused to N-terminal DNA-binding domain	TK0063	0.016	2.3	3	0.0027	1.5	4
Glycerol-1-phosphate dehydrogenase [NAD(P)+]	TK0789	0.042	3.5	2	0.021	1.3	3
Rubrerythrin domain-containing protein	TK0826	0.00001	3.8	4	0.00001	∞	5
L-threonine 3-dehydrogenase	TK0916	0.022	1.8	3	0.002	1.4	4
Uncharacterized protein	TK0930	0.00001	8.6	5	0.00001	10	5
Protein disulfide oxidoreductase	TK1085	0.0093	2.5	4	0.00001	1.7	4
Glutamate synthase beta chain-related oxidoreductase	TK2074	0.00038	2.2	4	0.00001	1.8	4
4Fe-4S cluster-binding protein	TK2075	0.00093	2.8	4	0.00013	1.6	4
Probable formate dehydrogenase, alpha subunit	TK2076	0.00001	1.9	4	0.00001	1.3	4
4Fe-4S cluster-binding protein	TK2077	0.00047	1.5	4	0.00001	1.6	4
4Fe-4S cluster-binding protein	TK2078	0.00001	3.8	4	0.00001	1.6	4
Membrane bound hydrogenase, 4Fe-4S cluster-binding subunit	TK2093	0.00001	4.7	5	0.012	1.4	3
Uncharacterized protein	TK0453	0.022	4	4			
Predicted ATPase, AAA superfamily, containing PIN and KH nucleic acid-binding domains	TK0953	0.026	2.2	3			
tRNA (1-methyladenosine) methyltransferase	TK1328	0.0035	9	5			
Proteasome subunit alpha	TK1637	0.016	3.3	3			
ABC-type dipeptide/oligopeptide transport system, permease component	TK1803	0.042	3.5	3			
Ferredoxin 3	TK2012	0.00001	4.7	5			
Iron-molybdenum cofactor-binding protein	TK2016	0.003	∞	5			
Enolase	TK2106	0.015	7	4			
Thermosome subunit beta	TK2303	0.011	1.3	3			
Uncharacterized protein	TK0011				0.00001	1.3	4
Flagellin B1	TK0038				0.00036	9	4
Indolepyruvate oxidoreductase subunit IorA	TK0136				0.00001	1.4	4
Formate-dependent phosphoribosylglycinamide formyltransferase	TK0207				0.02	3	2
Amidophosphoribosyltransferase	TK0211				0.00001	1.9	4
Acetyl-CoA synthetase I (NDP forming), beta subunit	TK0465				0.012	∞	3
Glyceraldehyde 3-phosphate phosphatase	TK0477				0.00001	1.3	4

DNA/RNA-binding protein Alba	TK0560				0.02	2.3	3
Molybdopterin oxidoreductase, molybdopterin-binding subunit	TK0690				0.043	2	2
ABC-type molybdate transport system, permease component	TK0718				0.012	∞	3
Uncharacterized protein	TK0744				0.013	1.5	3
Predicted ATP-dependent endonuclease, OLD family	TK0773				0.00001	3	4
Acetyl-CoA synthetase II (NDP forming), beta subunit	TK0943				0.045	2.5	2
Acetate--CoA ligase (ADP-forming)	TK0944				0.0039	3	4
Ribosome biogenesis protein Nop10	TK1101				0.045	2.5	3
Ribonuclease Z	TK1114				0.019	1.3	3
NAD(P)H sulfur oxidoreductase (CoA-dependent)	TK1299				0.00001	1.4	4
Phosphoenolpyruvate carboxykinase [GTP]	TK1405				0.0089	2.2	4
50S ribosomal protein L12	TK1415				0.00088	2.3	4
V-type ATP synthase subunit I	TK1597				0.012	∞	3
V-type ATP synthase subunit E	TK1599				0.037	∞	2
Methylmalonyl-CoA decarboxylase, alpha subunit	TK1622				0.01	1.4	4
DNA-directed RNA polymerase subunit N	TK1699				0.037	∞	4
Xanthine/guanine phosphoribosyltransferase	TK1737				0.0089	2.7	4
Glycogen synthase	TK1768				0.00001	∞	3
ABC-type dipeptide/oligopeptide transport system, ATPase component	TK1800				0.0017	3.3	4
ABC-type dipeptide/oligopeptide transport system, ATPase component	TK1801				0.0041	2.2	4
ABC-type dipeptide/oligopeptide transport system, permease component	TK1802				0.039	1.7	3
DNA polymerase II large subunit	TK1903				0.00001	1.4	4
Membrane bound hydrogenase, NiFe-hydrogenase small subunit	TK2089				0.037	∞	3

Table S16: Co-purified protein partners of FDHγ_4 (TK2078) in sulfur and non-sulfur conditions.

		+S°			-S°		
**Co-purifying proteins**	**Gene No.**	**p-value**	**Fold Change**	**Rank**	**p-value**	**Fold Change**	**Rank**
Uncharacterized protein	TK0930	0.00001	12	5	0.00001	14	5
Protein disulfide oxidoreductase	TK1085	0.00022	6.3	5	0.00001	1.7	4
NAD(P)H sulfur oxidoreductase (CoA-dependent)	TK1299	0.047	2.6	3	0.00001	1.5	4
4Fe-4S cluster-binding protein	TK2078	0.00001	8.2	5	0.00001	1.7	4
Nucleotidyltransferase, fused to N-terminal DNA-binding domain	TK0063	0.029	3.8	3			
Indolepyruvate oxidoreductase subunit IorA	TK0136	0.0021	7.7	5			
UPF0173 metal-dependent hydrolase TK0141	TK0141	0.00001	4.9	5			
RecJ-like exonuclease, containing OB-fold nucleic acid-binding domains	TK0155	0.009	2.9	4			

Fibrillarin-like rRNA/tRNA 2'-O-methyltransferase	TK0183	0.025	2.7	3
L-aspartate oxidase	TK0297	0.032	4.5	4
TatD-related deoxyribonuclease	TK0317	0.023	11	3
RNA-splicing ligase RtcB	TK0358	0.00025	12	5
Uncharacterized protein	TK0438	0.023	11	4
Uncharacterized protein	TK0453	0.0068	8.5	5
Uncharacterized protein	TK0483	0.00001	5.6	5
Flavin prenyltransferase UbiX	TK0509	0.0063	14	5
Metal-dependent phosphohydrolase, HD superfamily	TK0540	0.0096	6.3	5
Metallophosphoesterase, calcineurin superfamily	TK0574	0.0071	∞	5
Glycerol-1-phosphate dehydrogenase [NAD(P)+]	TK0789	0.01	8	5
Type 2 DNA topoisomerase 6 subunit A	TK0798	0.0052	2.8	4
Type 2 DNA topoisomerase 6 subunit B	TK0799	0.00012	3	4
Digeranylgeranylglycerophospholipid reductase	TK1088	0.039	5	4
Ribonuclease Z	TK1114	0.044	4.2	4
Transcription regulator, PadR-like family	TK1143	0.012	∞	4
Phosphoglycerate kinase	TK1146	0.036	4	4
Predicted AP endonuclease	TK1165	0.00001	4.4	5
Uncharacterized protein	TK1394	0.0041	15	5
Glycerol kinase	TK1396	0.015	12	3
Phosphomannomutase-related protein	TK1404	0.032	6.5	4
50S ribosomal protein L10	TK1416	0.015	7.5	4
1,4-alpha-glucan branching enzyme TK1436	TK1436	0.0026	∞	5
Leucine--tRNA ligase	TK1461	0.0096	6.3	5
50S ribosomal protein L4	TK1541	0.029	3.8	3
50S ribosomal protein L3	TK1542	0.035	3.2	3
Uncharacterized protein	TK1545	0.046	6	4
Predicted metal-dependent hydrolase	TK1611	0.00051	5.1	5
Methylmalonyl-CoA decarboxylase, alpha subunit	TK1622	0.033	3.4	3
Uncharacterized protein	TK1626	0.032	6.5	4
Proteasome subunit alpha	TK1637	0.028	5.3	4
Predicted transcription regulator, DUF118 helix-turn-helix family	TK1769	0.0097	13	5
Predicted hydrolase, metallo-beta-lactamase superfamily	TK1778	0.022	7	4
Probable tRNA/rRNA methyltransferase	TK1785	0.00001	6.8	5
Probable lipoprotein releasing system, ATP-binding protein	TK1859	0.0019	5.8	5
DNA polymerase II large subunit	TK1903	0.00001	3.8	4
2-oxoisovalerate:ferredoxin oxidoreductase, alpha subunit	TK1980	0.026	3.5	3
Iron-molybdenum cofactor-binding protein	TK2016	0.012	∞	4
SAM-dependent methyltransferase, UPF0020 family	TK2045	0.035	10	3

Cytosolic NiFe-hydrogenase, gamma subunit	TK2071	0.0071	∞	5			
Cytosolic NiFe-hydrogenase, beta subunit	TK2072	0.0043	∞	5			
Probable formate dehydrogenase, alpha subunit	TK2076	0.00001	3	4			
4Fe-4S cluster-binding protein	TK2077	0.01	2.4	4			
Enolase	TK2106	0.0026	16	5			
tRNA (cytosine(72)-C(5))-methyltransferase	TK2122	0.00001	∞	4			
Pantoate kinase	TK2141	0.032	6.5	4			
Radical_SAM domain-containing protein	TK2160	0.031	∞	4			
Tungsten-containing glyceraldehyde-3-phosphate:ferredoxin oxidoreductase	TK2163	0.019	∞	4			
Fructose-1,6-bisphosphate aldolase/phosphatase	TK2164	0.0041	15	5			
Zinc-dependent protease, TldD/PmbA family	TK2169	0.035	10	4			
tRNA-splicing endonuclease	TK2215	0.041	2.4	3			
Ribonuclease VapC	TK2228	0.035	10	4			
Deoxycytidylate deaminase	TK2257	0.015	12	4			
ATP-binding protein	TK2280	0.031	∞	4			
Anaerobic ribonucleoside-triphosphate reductase	TK2298	0.022	4	4			
Flagellin B2	TK0039				0.017	4	3
Flagellin B3	TK0040				0.017	∞	3
Indolepyruvate oxidoreductase subunit IorB	TK0135				0.002	1.7	4
Superoxide reductase	TK0525				0.017	∞	4
DNA/RNA-binding protein Alba	TK0560				0.011	2	3
Peroxiredoxin, AhpC/TSA family	TK0815				0.0011	∞	5
Rubrerythrin domain-containing protein	TK0826				0.00001	∞	5
Glucosamine-1-phosphate N-acetyltransferase	TK1188				0.00041	2.5	4
50S ribosomal protein L13	TK1501				0.041	2	3
Cysteine synthase	TK1687				0.0051	5	5
Glycogen synthase	TK1768				0.00001	∞	5

Table S17: Co-purified protein partners of MBS-L (TK1215) in sulfur and non-sulfur conditions.

		+S°			-S°		
**Co-purifying proteins**	**Gene No.**	**p-value**	**Fold Change**	**Rank**	**p-value**	**Fold Change**	**Rank**
Molybdate/tungstate-binding protein WtpA	TK0015	0.022	4.3	4	0.006	3.2	4
Nucleotidyltransferase, fused to N-terminal DNA-binding domain	TK0063	0.007	3	4	0.00001	3.7	4
Amidophosphoribosyltransferase	TK0211	0.0041	2.9	4	0.00001	4	5
Predicted ATP-dependent endonuclease, OLD family	TK0773	0.024	2.5	2	0.00001	8	5
Uncharacterized protein	TK0879	0.044	∞	2	0.0028	∞	5

Uncharacterized protein	TK0930	0.00001	14	5	0.00001	21	5
Membrane bound hydrogenase, NiFe-hydrogenase large subunit 2	TK1215	0.00001	∞	5	0.00001	67	5
Membrane bound hydrogenase, NiFe-hydrogenase large subunit 1	TK1216	0.00001	34	5	0.00001	85	5
Uncharacterized protein	TK1394	0.011	10	2	0.035	2	3
Glycogen synthase	TK1768	0.0037	∞	4	0.00001	∞	5
Ferredoxin 3	TK2012	0.00001	∞	5	0.00001	8.3	5
Membrane bound hydrogenase, NiFe-hydrogenase small subunit	TK2089	0.00001	∞	4	0.00001	7.5	5
Membrane bound hydrogenase, 4Fe-4S cluster-binding subunit	TK2093	0.00001	∞	5	0.00001	8.3	5
Fructose-1,6-bisphosphate aldolase/phosphatase	TK2164	0.0021	13	5	0.02	2.1	3
Flagellin B1	TK0038	0.008	6.5	5			
Predicted ATPase, AAA superfamily, containing PIN and KH nucleic acid-binding domains	TK0953	0.0011	4.3	5			
2-oxoacid:ferredoxin oxidoreductases, beta subunit	TK1129	0.001	∞	5			
tRNA (1-methyladenosine) methyltransferase	TK1328	0.0064	11	5			
tRNA/rRNA cytosine-C5-methylase, NOL1/NOP2/Sun family	TK1935	0.00001	19	5			
UPF0173 metal-dependent hydrolase TK0141	TK0141	0.0074	2.7	4			
Uncharacterized protein	TK0483	0.0019	2.4	4			
Flavin prenyltransferase UbiX	TK0509	0.0021	13	4			
Serine hydroxymethyltransferase	TK0528	0.0082	2.5	4			
Adenylosuccinate lyase	TK0561	0.0081	3.3	4			
ABC-type iron(III)-siderophore transport system, periplasmic component fused to N-terminal uncharacterized domain	TK0706	0.034	5	4			
Glycerol-1-phosphate dehydrogenase [NAD(P)+]	TK0789	0.021	5.5	4			
Type 2 DNA topoisomerase 6 subunit B	TK0799	0.0022	2	4			
S-layer protein	TK0895	0.00084	2	4			
Rubrerythrin-related protein	TK1056	0.024	∞	4			
2-oxoacid:ferredoxin oxidoreductases, alpha subunit	TK1130	0.013	6	4			
Predicted AP endonuclease	TK1165	0.00001	2.5	4			
DNA polymerase II large subunit	TK1903	0.001	2.1	4			
Probable formate transporter	TK2079	0.024	∞	4			
RecJ-like exonuclease, containing OB-fold nucleic acid-binding domains	TK0155	0.016	2.2	3			
Metallophosphoesterase, calcineurin superfamily	TK0574	0.002	∞	3			
Glycine--tRNA ligase	TK0978	0.038	2.8	3			
Translation initiation factor 2 subunit alpha	TK1100	0.013	3.6	3			
Phosphoglycerate kinase	TK1146	0.038	2.8	3			
Uncharacterized protein	TK1186	0.043	3	3			
Probable tRNA pseudouridine synthase B	TK1509	0.017	2.1	3			
V-type ATP synthase beta chain	TK1603	0.032	8	3			
Uncharacterized protein	TK1608	0.044	∞	3			

Translation initiation factor 2 subunit beta	TK1621	0.033	2.4	3			
Sugar-phosphate nucleotydyltransferase	TK1711	0.044	∞	3			
Predicted hydrolase, metallo-beta-lactamase superfamily	TK1778	0.021	5.5	3			
Probable lipoprotein releasing system, ATP-binding protein	TK1859	0.013	3.6	3			
Iron-molybdenum cofactor-binding protein	TK2016	0.024	∞	3			
4Fe-4S cluster-binding protein	TK2078	0.013	3.3	3			
Enolase	TK2106	0.032	3.5	3			
Putative 5-methylcytosine restriction system, catalytic subunit	TK1010	0.044	∞	2			
Ribonuclease Z	TK1114	0.032	3.5	2			
Predicted transcription regulator, DUF118 helix-turn-helix family	TK1769	0.024	∞	2			
Leucine--tRNA ligase	TK1461	0.043	3	1			
50S ribosomal protein L12	TK1415				0.00001	4.3	5
50S ribosomal protein L1	TK1417				0.0097	4	5
50S ribosomal protein L11	TK1418				0.00041	13	5
50S ribosomal protein L18e	TK1502				0.0057	9.3	5
ABC-type dipeptide/oligopeptide transport system, permease component	TK1803				0.008	5.3	5
SAM-dependent methyltransferase, UPF0020 family	TK2045				0.00001	∞	5
Flagellin B2	TK0039				0.0075	∞	4
Flagellin B5	TK0042				0.0075	∞	4
GMP synthase [glutamine-hydrolyzing] subunit A	TK0190				0.0063	2.1	4
Uncharacterized protein	TK0350				0.02	∞	4
CDC48/VCP homolog, AAA superfamily	TK0669				0.0021	2.7	4
Universal stress protein	TK0881				0.02	∞	4
L-threonine 3-dehydrogenase	TK0916				0.00069	2.6	4
Acetate--CoA ligase (ADP-forming)	TK0944				0.013	8	4
Ribosome biogenesis protein Nop10	TK1101				0.031	6.7	4
Phosphoenolpyruvate synthase	TK1292				0.0004	2.6	4
NAD(P)H sulfur oxidoreductase (CoA-dependent)	TK1299				0.00001	2.3	4
Uncharacterized protein	TK1313				0.00013	2.5	4
50S ribosomal protein L10	TK1416				0.00099	3.2	4
50S ribosomal protein L30	TK1520				0.0031	3.5	4
50S ribosomal protein L18	TK1522				0.0016	6.7	4
50S ribosomal protein L6	TK1525				0.00088	3.6	4
50S ribosomal protein L5	TK1528				0.037	4	4
V-type ATP synthase subunit C	TK1600				0.017	4.7	4
tRNA-splicing endonuclease	TK2215				0.00001	2.1	4
Lysine--tRNA ligase	TK2240				0.00001	2.3	4
Uncharacterized protein	TK0033				0.021	2.3	3

ABC-type transport system, probable periplasmic component	TK0657				0.035	2	3
DNA helicase	TK1199				0.039	3.1	3
50S ribosomal protein L14	TK1531				0.012	2.7	3
tRNA (cytosine(72)-C(5))-methyltransferase	TK2122				0.0075	∞	3
Uncharacterized protein	TK2144				0.038	2.2	3
tRNA/rRNA cytosine-C5-methylase, NOL1/NOP2/Sun family, fused to N-terminal NusB regulator domain	TK2304				0.00001	3.7	3
50S ribosomal protein L32e	TK1524				0.039	3.1	2
50S ribosomal protein L29	TK1535				0.02	∞	2

Table S18: Co-purified protein partners of GGR (TK1088) in sulfur and non-sulfur conditions.

		+S°			-S°		
**Co-purifying proteins**	**Gene No.**	**p-value**	**Fold Change**	**Rank**	**p-value**	**Fold Change**	**Rank**
Flagellin B1	TK0038	0.00001	6.8	5	0.00001	22	5
Flagellin B2	TK0039	0.00001	8.6	5	0.00001	∞	5
Flagellin B3	TK0040	0.00001	8.8	5	0.00001	∞	5
Flagellin B5	TK0042	0.00001	∞	4	0.00001	∞	5
Archaeal flagella-related protein D, internal insertion	TK0044	0.00001	5.1	5	0.00063	∞	3
Uncharacterized protein	TK0467	0.00001	4	5	0.00001	∞	5
Rubrerythrin domain-containing protein	TK0826	0.00001	5.3	5	0.00001	∞	5
S-layer protein	TK0895	0.00001	3.5	4	0.00001	2.8	4
Uncharacterized protein	TK0930	0.00001	5.5	5	0.00001	5	4
Ferredoxin 2	TK1087	0.00089	9.3	4	0.04	3	1
Digeranylgeranylglycerophospholipid reductase	TK1088	0.00001	7.6	5	0.00001	17	5
Ribosome biogenesis protein Nop10	TK1101	0.0004	3.7	4	0.0087	2.5	4
Archaeal flagella-related protein I, predicted secretion ATPase	TK0048	0.0018	∞	5			
Hypothetical membrane protein	TK0162	0.0065	∞	5			
Uncharacterized protein	TK0569	0.00001	2.3	4			
Glycerol-1-phosphate dehydrogenase [NAD(P)+]	TK0789	0.00043	2.1	4			
Probable vitamin B12 transport protein	TK0865	0.0011	5.3	5			
Uncharacterized protein	TK0900	0.00001	3.2	4			
ABC-type multidrug transport system, ATPase component	TK0942	0.023	∞	4			
Hypothetical membrane protein	TK1024	0.023	∞	3			
Uncharacterized protein	TK1313	0.00001	2.1	4			
50S ribosomal protein L10	TK1416	0.0014	2.2	4			
50S ribosomal protein L11	TK1418	0.00089	9.3	4			
Uncharacterized protein	TK1463	0.0076	3.1	4			

Probable tRNA pseudouridine synthase B	TK1509	0.00001	2.6	4			
50S ribosomal protein L14e	TK1513	0.016	2.3	3			
Protein-export membrane protein SecF	TK1593	0.0028	8	5			
V-type ATP synthase subunit I	TK1597	0.0011	5.3	5			
V-type ATP synthase subunit E	TK1599	0.025	5.3	4			
V-type ATP synthase subunit F	TK1601	0.023	∞	4			
V-type ATP synthase alpha chain	TK1602	0.00001	3	4			
Methylmalonyl-CoA decarboxylase, alpha subunit	TK1622	0.00011	2.7	4			
Exosome complex component Rrp41	TK1634	0.025	5.3	3			
ABC-type multidrug transport system, ATPase component	TK2161	0.023	∞	3			
LTD domain-containing protein	TK2178	0.0028	8	3			
Proteasome subunit beta 2	TK2207	0.0065	∞	5			
ABC-type iron(III) transport system, periplasmic component	TK0570				0.04	3	2
Peroxiredoxin, AhpC/TSA family	TK0815				0.0028	∞	4
Acetate--CoA ligase (ADP-forming)	TK0944				0.04	3	2
V-type ATP synthase subunit C	TK1600				0.0087	2.5	3
Ferredoxin 3	TK2012				0.00001	12	5
Membrane bound hydrogenase, 4Fe-4S cluster-binding subunit	TK2093				0.00001	12	5

Table S19: Co-purified protein partners of RBR1 (TK0650) in sulfur and non-sulfur conditions.

		+S°			-S°		
**Co-purifying proteins**	**Gene No.**	**p-value**	**Fold Change**	**Rank**	**p-value**	**Fold Change**	**Rank**
Amidophosphoribosyltransferase	TK0211	0.0012	19	5	0.00032	2.5	4
Rubrerythrin-related protein	TK0650	0.00001	∞	5	0.00001	350	5
Rubrerythrin domain-containing protein	TK0826	0.00094	∞	5	0.00088	∞	5
Aldehyde ferredoxin oxidoreductase	TK0844	0.0041	16	5	0.0037	3.2	4
Protein disulfide oxidoreductase	TK1085	0.006	∞	5	0.044	2	3
NAD(P)H sulfur oxidoreductase (CoA-dependent)	TK1299	0.0062	15	5	0.0021	1.9	4
Translation initiation factor 2 subunit beta	TK1621	0.039	∞	4	0.023	6	4
Uncharacterized protein	TK0166	0.00023	∞	5			
Fibrillarin-like rRNA/tRNA 2'-O-methyltransferase	TK0183	0.031	11	4			
snoRNP component, Nop56p/58p homolog	TK0184	0.015	∞	4			
Predicted membrane protease subunit, stomatin/prohibitin homolog	TK0348	0.0096	∞	5			
AMP phosphorylase	TK0352	0.018	2	3			
Uncharacterized protein	TK0442	0.0096	∞	5			
Diadenylate cyclase	TK0510	0.00001	29	5			

Serine hydroxymethyltransferase	TK0528	0.024	∞	4
DNA/RNA-binding protein Alba	TK0560	0.039	∞	4
Thermosome subunit alpha	TK0678	0.0032	4.3	5
Type 2 DNA topoisomerase 6 subunit A	TK0798	0.00023	∞	5
Type 2 DNA topoisomerase 6 subunit B	TK0799	0.00033	13	5
Uncharacterized protein	TK0900	0.039	∞	4
Uncharacterized protein	TK0930	0.00001	∞	5
Sugar-phosphate nucleotidyltransferase	TK0955	0.048	2.8	3
N2, N2-dimethylguanosine tRNA methyltransferase	TK0981	0.0054	6.2	5
Fructose-bisphosphate aldolase class 1	TK0989	0.015	∞	4
Putative 5-methylcytosine restriction system, GTPase subunit	TK1009	0.031	11	4
Uncharacterized protein	TK1025	0.00051	21	5
30S ribosomal protein S7	TK1077	0.021	12	4
Predicted AP endonuclease	TK1165	0.0037	5.8	5
Phosphoenolpyruvate synthase	TK1292	0.039	∞	4
Probable translation initiation factor IF-2	TK1305	0.00094	∞	5
30S ribosomal protein S28e	TK1310	0.045	10	4
DNA primase DnaG	TK1410	0.032	3	3
50S ribosomal protein L12	TK1415	0.039	∞	4
Cleavage and polyadenylation specificity factor subunit homolog	TK1428	0.031	11	4
Probable tRNA pseudouridine synthase B	TK1509	0.014	13	4
50S ribosomal protein L4	TK1541	0.006	∞	5
ABC-type multidrug transport system, ATPase component	TK1579	0.0038	∞	5
V-type ATP synthase alpha chain	TK1602	0.00001	∞	5
V-type ATP synthase beta chain	TK1603	0.006	∞	5
V-type ATP synthase subunit D	TK1604	0.024	∞	4
Predicted metal-dependent hydrolase	TK1611	0.0096	∞	5
Methylmalonyl-CoA decarboxylase, alpha subunit	TK1622	0.00001	∞	5
ABC-type dipeptide/oligopeptide transport system, ATPase component	TK1800	0.006	∞	5
ABC-type dipeptide/oligopeptide transport system, ATPase component	TK1801	0.00015	∞	5
ABC-type dipeptide/oligopeptide transport system, permease component	TK1802	0.0096	∞	5
ABC-type dipeptide/oligopeptide transport system, permease component	TK1803	0.006	∞	5
ABC-type dipeptide/oligopeptide transport system, probable periplasmic component	TK1804	0.0011	11	5
Type II/IV secretion system ATPase	TK1853	0.0056	3.5	4
Probable lipoprotein releasing system, ATP-binding protein	TK1859	0.0093	14	5

DNA polymerase II large subunit	TK1903	0.00001	∞	5			
Ferredoxin 3	TK2012	0.0038	∞	5			
ABC-type multidrug transport system, ATPase component	TK2053	0.039	∞	4			
Probable formate dehydrogenase, alpha subunit	TK2076	0.00017	2.8	4			
4Fe-4S cluster-binding protein	TK2078	0.0024	∞	5			
Membrane bound hydrogenase, NiFe-hydrogenase large subunit 2	TK2091	0.0062	15	5			
Membrane bound hydrogenase, 4Fe-4S cluster-binding subunit	TK2093	0.0038	∞	5			
Radical_SAM domain-containing protein	TK2160	0.039	∞	4			
cobW domain-containing protein	TK2200	0.018	4.1	4			
tRNA-splicing endonuclease	TK2215	0.024	∞	4			
Lysine--tRNA ligase	TK2240	0.015	∞	4			
Flagellin B5	TK0042				0.03	∞	4
Phosphoribosylaminoimidazole-succinocarboxamide synthase	TK0432				0.03	∞	3
Predicted ATP-dependent endonuclease, OLD family	TK0773				0.031	3.5	3
Glycogen synthase	TK1768				0.012	∞	3

Table S20: Co-purified protein partners of RBR2 (TK0826) in sulfur and non-sulfur conditions.

		+S°			-S°		
**Co-purifying proteins**	**Gene No.**	**p-value**	**Fold Change**	**Rank**	**p-value**	**Fold Change**	**Rank**
RecJ-like exonuclease, containing OB-fold nucleic acid-binding domains	TK0155	0.0049	28	5	0.014	4.6	4
Amidophosphoribosyltransferase	TK0211	0.014	9.1	4	0.00001	7.9	5
Metal-dependent phosphohydrolase, HD superfamily	TK0540	0.011	∞	4	0.0017	6.4	5
Predicted phosphohydrolase, DHH family	TK0645	0.044	∞	4	0.0081	∞	5
tRNA(Met) cytidine acetyltransferase TmcA	TK0754	0.0078	∞	5	0.035	4.6	4
Glycerol-1-phosphate dehydrogenase [NAD(P)+]	TK0789	0.031	∞	4	0.022	9.5	4
Type 2 DNA topoisomerase 6 subunit A	TK0798	0.015	10	4	0.0052	5.1	5
Rubrerythrin domain-containing protein	TK0826	0.00001	∞	5	0.00001	∞	5
Aldehyde ferredoxin oxidoreductase	TK0844	0.00001	45	5	0.016	8	4
Ribonuclease Z	TK1114	0.044	∞	4	0.00082	25	5
Phosphoenolpyruvate synthase	TK1292	0.022	∞	4	0.02	7.7	4
NAD(P)H sulfur oxidoreductase (CoA-dependent)	TK1299	0.00035	23	5	0.00019	8	5
Probable translation initiation factor IF-2	TK1305	0.00001	∞	5	0.0044	5	5
Uncharacterized protein	TK1329	0.016	∞	4	0.0027	∞	5
Translation initiation factor 2 subunit beta	TK1621	0.031	∞	4	0.011	17	4
Ferredoxin:NADP oxidoreductase, alpha subunit	TK1684	0.031	∞	4	0.023	6.5	4

Vitamin B12-dependent ribonucleotide reductase	TK1736	0.00021	7.7	5	0.0014	3.4	4
Xanthine/guanine phosphoribosyltransferase	TK1737	0.044	∞	4	0.004	9.7	5
DNA repair and recombination protein RadA	TK1899	0.016	∞	4	0.0081	∞	5
DNA polymerase II large subunit	TK1903	0.00001	19	5	0.0024	3.8	4
tRNA-splicing endonuclease	TK2215	0.00017	∞	5	0.00018	7.1	5
Flagellin B5	TK0042	0.022	∞	4			
Fibrillarin-like rRNA/tRNA 2'-O-methyltransferase	TK0183	0.031	10	4			
snoRNP component, Nop56p/58p homolog	TK0184	0.041	12	4			
Pyridoxal 5'-phosphate synthase subunit PdxS	TK0217	0.0089	12	5			
Quinolinate synthase A	TK0296	0.031	∞	4			
AMP phosphorylase	TK0352	0.0011	2.6	4			
RNA-splicing ligase RtcB	TK0358	0.031	∞	4			
Uncharacterized protein	TK0440	0.03	20	3			
Uncharacterized protein	TK0453	0.0055	∞	5			
Uncharacterized protein	TK0483	0.031	∞	4			
Serine hydroxymethyltransferase	TK0528	0.00077	36	5			
Molybdenum cofactor biosynthesis protein B	TK0544	0.0033	2.4	4			
Proline--tRNA ligase	TK0550	0.022	∞	3			
GTP cyclohydrolase MptA	TK0793	0.031	∞	4			
Type 2 DNA topoisomerase 6 subunit B	TK0799	0.00083	10	5			
Uncharacterized protein	TK0900	0.04	19	4			
Predicted ATPase, AAA superfamily, containing PIN and KH nucleic acid-binding domains	TK0953	0.0078	∞	5			
Glycine--tRNA ligase	TK0978	0.04	19	4			
Fructose-bisphosphate aldolase class 1	TK0989	0.032	13	4			
Putative 5-methylcytosine restriction system, GTPase subunit	TK1009	0.039	9.8	4			
Uncharacterized protein	TK1025	0.04	19	4			
DNA-directed RNA polymerase subunit	TK1076	0.0027	31	5			
DNA-directed RNA polymerase subunit beta	TK1083	0.0014	16	5			
Protein disulfide oxidoreductase	TK1085	0.00098	∞	5			
2-oxoacid:ferredoxin oxidoreductases, alpha subunit	TK1130	0.022	∞	4			
Peptide chain release factor subunit 1	TK1239	0.022	∞	4			
Nucleoside diphosphate kinase	TK1307	0.0039	∞	5			
Ferredoxin:NADP oxidoreductase, beta subunit	TK1326	0.016	∞	4			
Cell division protein FtsZ 1	TK1421	0.0039	∞	4			
Cleavage and polyadenylation specficity factor subunit homolog	TK1428	0.0011	16	5			
Leucine--tRNA ligase	TK1461	0.0039	∞	5			
Isopentenyl-diphosphate delta-isomerase	TK1470	0.022	∞	4			
V-type ATP synthase alpha chain	TK1602	0.012	24	4			

Ribosome maturation protein SDO1 homolog	TK1636	0.0027	19	5			
Ferredoxin:NADP oxidoreductase, beta subunit	TK1685	0.022	∞	4			
ABC-type dipeptide/oligopeptide transport system, ATPase component	TK1800	0.04	19	4			
ABC-type dipeptide/oligopeptide transport system, ATPase component	TK1801	0.04	19	4			
ABC-type dipeptide/oligopeptide transport system, permease component	TK1803	0.022	∞	4			
ABC-type dipeptide/oligopeptide transport system, probable periplasmic component	TK1804	0.04	7.7	4			
Type II/IV secretion system ATPase	TK1853	0.024	5.3	4			
Probable lipoprotein releasing system, ATP-binding protein	TK1859	0.0014	33	5			
Predicted N6-adenine-specific DNA methylase	TK1863	0.044	∞	4			
5-methylthioadenosine/S-adenosylhomocysteine deaminase	TK1891	0.011	∞	4			
Iron-molybdenum cofactor-binding protein	TK2016	0.044	∞	4			
Enolase	TK2106	0.022	21	4			
Uncharacterized protein	TK2148	0.022	∞	3			
Radical_SAM domain-containing protein	TK2160	0.011	∞	4			
Tungsten-containing glyceraldehyde-3-phosphate:ferredoxin oxidoreductase	TK2163	0.022	∞	4			
Lysine--tRNA ligase	TK2240	0.0024	15	5			
Anaerobic ribonucleoside-triphosphate reductase	TK2298	0.00024	∞	5			
Flagellin B3	TK0040				0.0056	∞	5
Sugar-phosphate nucleotidyltransferase	TK0219				0.029	4.8	4
Uncharacterized protein	TK0426				0.036	∞	4
Phosphoribosylaminoimidazole-succinocarboxamide synthase	TK0432				0.00001	∞	5
Predicted GTPase, containing TGS domain	TK0506				0.036	∞	4
Diadenylate cyclase	TK0510				0.035	3.4	3
Predicted ATP-dependent endonuclease, OLD family	TK0773				0.0038	12	5
Uncharacterized protein	TK0930				0.00001	32	5
Sugar-phosphate nucleotidyltransferase	TK0955				0.0033	3.5	4
Adenylosuccinate synthetase	TK1002				0.0091	8.7	5
Translation initiation factor 2 subunit alpha	TK1100				0.0068	11	5
Uncharacterized protein	TK1186				0.018	6.8	4
ATP-dependent DNA helicase Hel308	TK1332				0.025	∞	4
Probable glycine dehydrogenase (decarboxylating) subunit 2	TK1379				0.029	9	4
Uncharacterized protein	TK1394				0.0056	∞	5
Probable tRNA pseudouridine synthase B	TK1509				0.017	4.5	4
Uncharacterized protein	TK1545				0.036	∞	4
Predicted dehydrogenase	TK1557				0.00049	3.7	4
Glycogen synthase	TK1768				0.00001	∞	5

Translation initiation factor 2 subunit gamma	TK1946				0.041	3.6	3
Carboxymuconolactone decarboxylase-related protein	TK1974				0.017	∞	4
Cysteine desulfurase	TK1990				0.017	4.4	4
Ferredoxin 3	TK2012				0.014	4.5	4
Cytosolic NiFe-hydrogenase, gamma subunit	TK2071				0.022	15	4
Cytosolic NiFe-hydrogenase, beta subunit	TK2072				0.0083	18	5
Membrane bound hydrogenase, NiFe-hydrogenase small subunit	TK2089				0.014	4.6	4
Membrane bound hydrogenase, 4Fe-4S cluster-binding subunit	TK2093				0.014	4.5	4
Radical_SAM domain-containing protein	TK2145				0.036	∞	4
Non-specific serine/threonine protein kinase	TK2250				0.02	7.7	4
Cell division protein FtsZ 2	TK2271				0.034	7	4

Table S21: Co-purified protein partners of GAPOR (TK2163) in sulfur and non-sulfur conditions.

		+S˚			-S˚			-P		
**Co-purifying proteins**	**Gene No.**	**p-value**	**Fold Change**	**Rank**	**p-value**	**Fold Change**	**Rank**	**p-value**	**Fold Change**	**Rank**
Tungsten-containing glyceraldehyde-3-phosphate:ferredoxin oxidoreductase	TK2163	0.00001	∞	5	0.00001	∞	5	0.00001	∞	5
Hydrolase, HAD superfamily	TK0110	0.0027	∞	5	0.032	∞	4			
Hypothetical membrane protein, conserved, containing DUF11 domain	TK0493	0.014	3.4	3	0.0029	∞	5			
DNA polymerase II large subunit	TK1903	0.023	3.7	3	0.00053	∞	5			
Uncharacterized protein	TK0426	0.021	∞	4						
ABC-type transport system, permease component	TK0659	0.032	6.7	4						
NAD-dependent protein deacylase	TK0685	0.021	7.3	4						
Zinc-dependent protease, TldD/PmbA family	TK0698	0.016	∞	4						
Proton/glutamate symporter, SDF family	TK0986	0.0097	∞	5						
Archaeal/vacuolar-type H+-ATPase, subunit H	TK1596	0.045	∞	4						
Pyruvate:ferredoxin oxidoreductase, beta subunit	TK1984	0.026	7	4						
Membrane bound hydrogenase, 4Fe-4S cluster-binding subunit	TK2093	0.047	3.2	3						
Replication factor C small subunit	TK2218	0.0093	5.3	5						
ABC-type dipeptide/oligopeptide transport system, ATPase component	TK1800				0.032	7	4	0.017	2	3
Molybdenum cofactor biosynthesis protein B	TK0544				0.044	2.5	3			
ABC-type iron(III)-siderophore transport system, periplasmic component fused to N-terminal uncharacterized domain	TK0706				0.0016	28	5			

Predicted ATP-dependent endonuclease, OLD family	TK0773				0.012	∞	4			
Oligosaccharyl transferase	TK0810				0.048	∞	4			
Predicted ATPase, AAA superfamily, containing PIN and KH nucleic acid-binding domains	TK0953				0.022	18	4			
Putative 5-methylcytosine restriction system, GTPase subunit	TK1009				0.017	19	4			
V-type ATP synthase subunit C	TK1600				0.014	∞	4			
V-type ATP synthase beta chain	TK1603				0.032	7	4			
ABC-type dipeptide/oligopeptide transport system, probable periplasmic component	TK1804				0.0044	7.7	5			
Metallophosphoesterase, calcineurin superfamily	TK0574							0.0056	∞	5
Glutamate dehydrogenase	TK1431							0.032	2.3	3

Table S22: Co-purified protein partners of FNOR1α (TK1325) in sulfur and non-sulfur conditions.

		+S°			-S°		
**Co-purifying proteins**	**Gene No.**	**p-value**	**Fold Change**	**Rank**	**p-value**	**Fold Change**	**Rank**
Ferredoxin:NADP oxidoreductase, alpha subunit	TK1325	0.00001	120	5	0.00001	∞	5
Ferredoxin:NADP oxidoreductase, beta subunit	TK1326	0.00001	∞	5	0.0001	∞	5
Ferredoxin:NADP oxidoreductase, beta subunit	TK1685	0.0003	5	5	0.0017	∞	5
2-oxoacid:ferredoxin oxidoreductases, alpha subunit	TK1125	0.046	5	4			
Phosphomannomutase-related protein	TK1404	0.029	3.5	3			
Archaeal histone A	TK1413	0.0017	5.5	5			
Membrane bound hydrogenase, 4Fe-4S cluster-binding subunit	TK2093	0.028	∞	4			

Table S23: Co-purified protein partners of FNOR1β (TK1326) in sulfur and non-sulfur conditions.

		+S°			-S°		
**Co-purifying proteins**	**Gene No.**	**p-value**	**Fold Change**	**Rank**	**p-value**	**Fold Change**	**Rank**
Ferredoxin:NADP oxidoreductase, beta subunit	TK1326	0.00001	∞	5	0.00001	∞	5
UPF0173 metal-dependent hydrolase TK0141	TK0141	0.001	6.5	5			
RNA-splicing ligase RtcB	TK0358	0.042	∞	3			
Uncharacterized protein	TK0483	0.026	10	3			
Serine hydroxymethyltransferase	TK0528	0.0066	13	4			
Uncharacterized protein	TK0569	0.0002	7.5	5			
Glycerol-1-phosphate dehydrogenase [NAD(P)+]	TK0789	0.042	∞	3			
Type A flavoprotein	TK0814	0.0041	4	5			

S-layer protein	TK0895	0.033	2.3	3			
D-aminopeptidase	TK1022	0.042	∞	3			
2-oxoacid:ferredoxin oxidoreductases, alpha subunit	TK1125	0.001	∞	5			
Predicted AP endonuclease	TK1165	0.00001	4.7	4			
Phosphoenolpyruvate synthase	TK1292	0.0051	∞	4			
Cell division protein FtsZ 1	TK1421	0.025	∞	2			
Cleavage and polyadenylation specficity factor subunit homolog	TK1428	0.0026	7	4			
Phosphopyruvate hydratase	TK1497	0.04	9	3			
Probable tRNA pseudouridine synthase B	TK1509	0.0065	8	4			
V-type ATP synthase alpha chain	TK1602	0.0026	15	4			
V-type ATP synthase beta chain	TK1603	0.026	10	3			
ABC-type dipeptide/oligopeptide transport system, permease component	TK1803	0.042	∞	3			
ABC-type dipeptide/oligopeptide transport system, probable periplasmic component	TK1804	0.00042	6.2	5			
Membrane bound hydrogenase, MbhH subunit	TK2087	0.042	∞	3			
Membrane bound hydrogenase, NiFe-hydrogenase large subunit 2	TK2091	0.0098	7.5	4			
cobW domain-containing protein	TK2200	0.00001	4.2	5			
tRNA-splicing endonuclease	TK2215	0.015	7	3			
ABC-type transport system involved in Fe-S cluster assembly, permease component	TK0730				0.027	4	2
Predicted ATP-dependent endonuclease, OLD family	TK0773				0.047	4.5	3
Type 2 DNA topoisomerase 6 subunit A	TK0798				0.046	2	2
Aldehyde ferredoxin oxidoreductase	TK0844				0.00052	9	3
Uncharacterized protein	TK0930				0.00001	28	4
NAD(P)H sulfur oxidoreductase (CoA-dependent)	TK1299				0.0033	3.4	4
Cytosolic NiFe-hydrogenase, beta subunit	TK2072				0.039	7.5	2

Table S24: Co-purified protein partners of FNOR2α (TK1684) in sulfur and non-sulfur conditions.

		+S°			-S°		
**Co-purifying proteins**	**Gene No.**	**p-value**	**Fold Change**	**Rank**	**p-value**	**Fold Change**	**Rank**
Indolepyruvate oxidoreductase subunit IorA	TK0136	0.015	2.9	3	0.00001	1.8	4
Indolepyruvate oxidoreductase subunit IorA	TK0136	0.00001	1.8	4	0.015	2.9	3
2-oxoacid:ferredoxin oxidoreductases, gamma subunit	TK1123	0.014	6	4	0.019	1.9	3
2-oxoacid:ferredoxin oxidoreductases, gamma subunit	TK1123	0.019	1.9	3	0.014	6	4
V-type ATP synthase beta chain	TK1603	0.024	5.3	4	0.03	2.2	3
V-type ATP synthase beta chain	TK1603	0.03	2.2	3	0.024	5.3	4
Ferredoxin:NADP oxidoreductase, alpha subunit	TK1684	0.00001	27	5	0.00001	22	5

Ferredoxin:NADP oxidoreductase, alpha subunit	TK1684	0.00001	22	5	0.00001	27	5
Ferredoxin:NADP oxidoreductase, beta subunit	TK1685	0.00001	78	5	0.00001	30	5
Ferredoxin:NADP oxidoreductase, beta subunit	TK1685	0.00001	30	5	0.00001	78	5
Molybdate/tungstate-binding protein WtpA	TK0015	0.025	3.3	3			
Flagellin B1	TK0038	0.00001	9	5			
Flagellin B2	TK0039	0.00019	3.1	4			
Flagellin B3	TK0040	0.015	2.9	3			
Flagellin B5	TK0042	0.015	2.9	3			
Archaeal flagella-related protein D, internal insertion	TK0044	0.019	∞	3			
L-threonine 3-dehydrogenase	TK0916	0.0065	2.1	4			
DNA-directed RNA polymerase subunit A''	TK1699	0.014	6	4			
Predicted transcription regulator, DUF118 helix-turn-helix family	TK1769	0.019	∞	4			
Enolase	TK2106	0.024	5.3	4			
Nucleotidyltransferase, fused to N-terminal DNA-binding domain	TK0063				0.00022	2.6	4
Uncharacterized protein	TK0130				0.039	5	4
Amidophosphoribosyltransferase	TK0211				0.0024	2.1	4
Uncharacterized protein	TK0226				0.025	∞	4
Predicted membrane protease subunit, stomatin/prohibitin homolog	TK0348				0.0036	3.7	4
ABC-type iron(III) transport system, ATPase component	TK0572				0.0053	2.6	4
ABC-type molybdate transport system, permease component	TK0718				0.0098	∞	5
tRNA(Met) cytidine acetyltransferase TmcA	TK0754				0.00001	1.8	4
Glyceraldehyde-3-phosphate dehydrogenase	TK0765				0.048	3	3
Predicted ATP-dependent endonuclease, OLD family	TK0773				0.0059	2.3	4
Probable vitamin B12 transport protein	TK0865				0.018	6	4
Uncharacterized protein	TK0869				0.018	6	4
Sulfur transfer protein involved in thiamine biosynthesis	TK1093				0.0039	∞	5
2-oxoacid:ferredoxin oxidoreductases, gamma subunit	TK1126				0.0039	∞	4
Glucosamine-1-phosphate N-acetyltransferase	TK1188				0.00085	4.3	5
DNA helicase	TK1199				0.00041	4.7	5
Phosphoenolpyruvate synthase	TK1292				0.00019	2.2	4
50S ribosomal protein L12	TK1415				0.0032	2.5	4
Signal recognition particle 54 kDa protein	TK1486				0.018	6	4
50S ribosomal protein L14e	TK1513				0.028	2.7	3
Protein translocase subunit SecY	TK1518				0.012	4	4
Archaeal/vacuolar-type H+-ATPase, subunit H	TK1596				0.039	5	4
V-type ATP synthase subunit C	TK1600				0.018	6	4
V-type ATP synthase alpha chain	TK1602				0.0011	2.6	4
Proteasome subunit alpha	TK1637				0.0036	2.3	4

Cysteine synthase	TK1687				0.0081	7	5
ABC-type dipeptide/oligopeptide transport system, ATPase component	TK1800				0.0036	3.7	4
Carboxymuconolactone decarboxylase-related protein	TK1974				0.039	5	4
Membrane bound hydrogenase, MbhA subunit	TK2080				0.025	∞	3
Membrane bound hydrogenase, MbhH subunit	TK2087				0.0098	∞	5
SPASM domain-containing protein	TK2114				0.0039	∞	5
Beta-ribofuranosylaminobenzene 5'-phosphate synthase	TK2242				0.0098	∞	5
Uncharacterized protein	TK2283				0.0012	5.5	5

Table S25: Co-purified protein partners of FNOR2β (TK1685) in sulfur and non-sulfur conditions.

		+S°			-S°		
**Co-purifying proteins**	**Gene No.**	**p-value**	**Fold Change**	**Rank**	**p-value**	**Fold Change**	**Rank**
Predicted ATP-dependent endonuclease, OLD family	TK0773	0.0052	3.9	3	0.00001	4.6	5
Predicted AP endonuclease	TK1165	0.00001	4.4	5	0.00001	2.1	4
Ferredoxin:NADP oxidoreductase, alpha subunit	TK1325	0.00001	98	5	0.00001	11	5
Ferredoxin:NADP oxidoreductase, beta subunit	TK1685	0.00001	96	5	0.00001	15	5
Amidophosphoribosyltransferase	TK0211	0.026	3.2	3	0.00026	2.1	4
Uncharacterized protein	TK0930	0.012	4.1	4	0.00028	3.2	4
Uncharacterized protein	TK2144	0.049	6	4	0.001	3.2	4
Metallophosphoesterase, calcineurin superfamily	TK0547	0.042	5	3	0.0083	3	3
V-type ATP synthase beta chain	TK1603	0.0012	18	5	0.017	2.7	2
Membrane bound hydrogenase, 4Fe-4S cluster-binding subunit	TK2093	0.00001	∞	5	0.018	∞	3
Uncharacterized protein	TK0022	0.012	∞	2			
Flagellin B1	TK0038	0.049	6	3			
UPF0173 metal-dependent hydrolase TK0141	TK0141	0.00029	4.2	5			
RNA-splicing ligase RtcB	TK0358	0.021	5.7	4			
Uncharacterized protein	TK0440	0.033	3.8	3			
PDDEXK_1 domain-containing protein	TK0446	0.02	∞	4			
Uncharacterized protein	TK0453	0.0085	5	5			
Uncharacterized protein	TK0483	0.00001	3.7	4			
Flavin prenyltransferase UbiX	TK0509	0.011	13	4			
Metal-dependent phosphohydrolase, HD superfamily	TK0540	0.0054	4.8	5			
Metallophosphoesterase, calcineurin superfamily	TK0574	0.0076	∞	4			
ABC-type iron(III)-siderophore transport system, periplasmic component fused to N-terminal uncharacterized domain	TK0706	0.049	6	4			
ABC-type molybdate transport system, ATPase component	TK0719	0.034	6.5	3			
tRNA(Met) cytidine acetyltransferase TmcA	TK0754	0.014	3.7	3			

Glycerol-1-phosphate dehydrogenase [NAD(P)+]	TK0789	0.03	5.3	4	
Type 2 DNA topoisomerase 6 subunit B	TK0799	0.0014	2.6	4	
Aldehyde ferredoxin oxidoreductase	TK0844	0.017	3.8	3	
S-layer protein	TK0895	0.01	2.4	4	
L-threonine 3-dehydrogenase	TK0916	0.016	3.4	3	
Predicted ATPase, AAA superfamily, containing PIN and KH nucleic acid-binding domains	TK0953	0.015	4	4	
tRNA (guanine(26)-N(2))-dimethyltransferase	TK0970	0.024	11	3	
Glycine--tRNA ligase	TK0978	0.01	4.5	5	
Putative 5-methylcytosine restriction system, catalytic subunit	TK1010	0.033	∞	3	
Uncharacterized protein	TK1025	0.034	6.5	3	
Predicted glutamine amidotransferase, class II	TK1035	0.0045	15	4	
2-oxoacid:ferredoxin oxidoreductases, gamma subunit	TK1123	0.016	7.5	4	
2-oxoacid:ferredoxin oxidoreductases, alpha subunit	TK1125	0.024	7	4	
2-oxoacid:ferredoxin oxidoreductases, alpha subunit	TK1130	0.034	6.5	3	
Phosphoglycerate kinase	TK1146	0.0021	5.3	5	
TBP-interacting protein	TK1172	0.012	∞	3	
Predicted ATPase, AAA superfamily	TK1314	0.029	3.5	3	
Ferredoxin:NADP oxidoreductase, beta subunit	TK1326	0.033	∞	3	
Uncharacterized protein	TK1394	0.011	13	3	
Glycerol kinase	TK1396	0.016	12	3	
50S ribosomal protein L10	TK1416	0.049	6	4	
Probable tRNA pseudouridine synthase B	TK1509	0.00011	3.8	4	
30S ribosomal protein S8	TK1526	0.012	4.8	4	
50S ribosomal protein L2	TK1539	0.016	4.6	4	
tRNA uridine(34) acetyltransferase	TK1574	0.0012	18	5	
Predicted metal-dependent hydrolase	TK1611	0.0035	4.7	5	
DNA-directed RNA polymerase subunit A''	TK1699	0.034	6.5	4	
Sugar-phosphate nucleotydyltransferase	TK1711	0.012	∞	3	
Vitamin B12-dependent ribonucleotide reductase	TK1736	0.0048	2.1	4	
Predicted transcription regulator, DUF118 helix-turn-helix family	TK1769	0.0004	∞	4	
ABC-type dipeptide/oligopeptide transport system, probable periplasmic component	TK1804	0.0028	3.3	4	
Probable lipoprotein releasing system, ATP-binding protein	TK1859	0.016	4.6	4	
Predicted N6-adenine-specific DNA methylase	TK1863	0.035	3.6	3	
Ferredoxin 3	TK2012	0.00001	∞	5	
Iron-molybdenum cofactor-binding protein	TK2016	0.033	∞	4	
Probable formate dehydrogenase, alpha subunit	TK2076	0.0012	2.1	4	
Membrane bound hydrogenase, NiFe-hydrogenase small subunit	TK2089	0.0047	∞	3	

Membrane bound hydrogenase, NiFe-hydrogenase large subunit 2	TK2091	0.039	3.2	3			
Enolase	TK2106	0.013	5.2	4			
Radical_SAM domain-containing protein	TK2160	0.033	∞	3			
Tungsten-containing glyceraldehyde-3-phosphate:ferredoxin oxidoreductase	TK2163	0.0047	∞	5			
tRNA-splicing endonuclease	TK2215	0.012	2.6	3			
Non-specific serine/threonine protein kinase	TK2250	0.0082	2.7	4			
Lysine--tRNA ligase	TK2240				0.00001	2	4
Uncharacterized protein	TK1046				0.0001	6.5	5
Arginase	TK0240				0.00025	6	5
GMP synthase [glutamine-hydrolyzing] subunit A	TK0190				0.00044	2.2	4
Nucleotidyltransferase, fused to N-terminal DNA-binding domain	TK0063				0.0008	2.2	4
Protein translocase subunit SecY	TK1518				0.00092	∞	5
Fructose-bisphosphate aldolase class 1	TK0989				0.0012	2.8	4
ABC-type dipeptide/oligopeptide transport system, permease component	TK1803				0.0025	∞	4
Flagellin B3	TK0040				0.0068	∞	3
50S ribosomal protein L1	TK1417				0.0069	4	5
V-type ATP synthase alpha chain	TK1602				0.0083	3	3
50S ribosomal protein L14e	TK1513				0.0091	2.5	4
ABC-type dipeptide/oligopeptide transport system, ATPase component	TK1800				0.0091	2.5	4
Transcription initiation factor IIB 1	TK1280				0.015	3.5	3
Uncharacterized protein	TK1609				0.017	2.7	2
Uncharacterized protein	TK0130				0.018	∞	3
XPA-binding protein 1 homolog	TK0951				0.018	∞	2
Archaeal/vacuolar-type H+-ATPase, subunit H	TK1596				0.018	∞	3
DNA polymerase II small subunit	TK1902				0.018	2.2	2
Probable formate transporter	TK2079				0.028	5	4
Putative 5-methylcytosine restriction system, GTPase subunit	TK0795				0.035	2.3	2
